# Collider Bias Assessment in Colombian Indigenous Wiwa and Kogui Populations with Chronic Gastroenteric Disorder of Likely Infectious Etiology Suggests Complex Microbial Interactions Rather Than Clear Assignments of Etiological Relevance

**DOI:** 10.3390/microorganisms12050970

**Published:** 2024-05-11

**Authors:** Hagen Frickmann, Joy Backhaus, Achim Hoerauf, Ralf Matthias Hagen, Simone Kann

**Affiliations:** 1 Department of Medical Microbiology, Virology and Hygiene, University Medicine Rostock, 18057 Rostock, Germany; 2 Department of Microbiology and Hospital Hygiene, Bundeswehr Hospital Hamburg, 20359 Hamburg, Germany; 3 Statistical Consulting, 97074 Wuerzburg, Germany; statistik.backhaus@gmail.com; 4 Institute of Medical Microbiology, Immunology and Parasitology (IMMIP), University Hospital Bonn, 53127 Bonn, Germany; achim.hoerauf@ukb.uni-bonn.de; 5 Department of Microbiology and Hospital Hygiene, Bundeswehr Central Hospital Koblenz, 56070 Koblenz, Germany; ralfmatthiashagen@bundeswehr.org

**Keywords:** gastroenteritis, etiology, tropics, high endemicity, resource limited, bacteria, protozoa, helminths, microsporidia, fungi

## Abstract

Multiple microbial detections in stool samples of indigenous individuals suffering from chronic gastroenteric disorder of a likely infectious origin, characterized by recurring diarrhea of variable intensity, in the rural north-east of Colombia are common findings, making the assignment of etiological relevance to individual pathogens challenging. In a population of 773 indigenous people from either the tribe Wiwa or Kogui, collider bias analysis was conducted comprising 32 assessed microorganisms including 10 bacteria (*Aeromonas* spp., *Campylobacter* spp., enteroaggregative *Escherichia coli* (EAEC), enteropathogenic *Escherichia coli* (EPEC), enterotoxigenic *Escherichia coli* (ETEC), *Salmonella* spp., Shiga toxin-producing *Escherichia coli* (STEC), *Shigella* spp./enteroinvasive *Escherichia coli* (EIEC), *Tropheryma whipplei* and *Yersinia* spp.), 11 protozoa (*Blastocystis* spp., *Cryptosporidium* spp., *Cyclospora* spp., *Dientamoeba fragilis*, *Entamoeba coli*, *Entamoeba bangladeshi*/*dispar*/*histolytica*/*moshkovskii* complex, *Entamoeba histolytica*, *Endolimax nana*, *Giardia duodenalis*, *Iodamoeba buetschlii* and *Pentatrichomonas hominis*), 8 helminths (*Ascaris* spp., *Enterobius vermicularis*, *Hymenolepis* spp., *Necator americanus*, *Schistosoma* spp., *Strongyloides* spp., *Taenia* spp. and *Trichuris* spp.), microsporidia (*Encephalocytozoon* spp.) and fungal elements (microscopically observed conidia and pseudoconidia). The main results indicated that negative associations potentially pointing towards collider bias were infrequent events (n = 14), while positive associations indicating increased likelihood of co-occurrence of microorganisms quantitatively dominated (n = 88). Microorganisms showing the most frequent negative associations were EPEC (n = 6) and *Blastocystis* spp. (n = 3), while positive associations were most common for *Trichuris* spp. (n = 16), *Dientamoeba fragilis* (n = 15), *Shigella* spp./EIEC (n = 12), *Ascaris* spp. (n = 11) and *Blastocystis* spp. (n = 10). Of note, positive associations quantitively dominated for *Blastocystis* spp. In conclusion, collider bias assessment did not allow clear-cut assignment of etiological relevance for detected enteric microorganisms within the assessed Colombian indigenous population. Instead, the results suggested complex microbial interactions with potential summative effects. Future studies applying alternative biostatistical approaches should be considered to further delineate respective interactions.

## 1. Introduction

In remote rural areas of the Sierra Nevada de Santa Marta in the north-east of Colombia, indigenous tribes called Wiwa and Kogui live under very resource-poor conditions, e.g., without hygiene standards (sanitation, clean drinking water, electricity, etc.) known from resource-rich industrialized countries. Instead, they live closely together with their livestock and use top dressing as a fertilizer. Therefore, smear infections, food- and water-borne infections as well as soil-transmitted infections can easily occur. Consequently, infectious gastroenteritis, which is commonly spread by such modes of transmission, is among the most pressing health issues in the local population, as recently shown by our group [1].

In both Wiwa and Kogui populations, surveillance assessments including both stool microscopy and molecular diagnostic approaches have been conducted [1,2,3]. Shortly summarized, the assessed indigenous populations suffered from the medical condition of virtually constant chronic gastroenteric disorders of a likely infectious etiology with only gradual changes in intensity over time, associated with disturbed stool frequency and consistency. As this state of gastroenteric disturbance was considered “normal” in the local population, respective complaints were socially discouraged. Consequently, the indigenous only showed up for medical assessment and therapy if disease symptoms reached unbearable or critical states, e.g., in the case of bloody diarrhea. In line with this socio-culturally maintained state of chronic gastroenteric disorder in the assessed indigenous populations, high rates of enteric pathogens were detected in their stool specimens during the surveillance assessments associated with high rates of multiple detections within the same sample, as previously described [1,2,3].

As previously shown [4,5,6,7], however, multiple pathogen detections in primarily non-sterile compartments like the human gastrointestinal tract make the assignment of etiological relevance for decisions on antimicrobial therapy challenging [4,5,6,7]. The reason is that most pathogens associated with infectious gastroenteritis, with rare exceptions like mycobacteria of the tuberculosis complex, show so-called “facultative pathogenicity”. This means that they can both occur as causative agents of infectious gastroenteritis and as harmless colonizers or elements of the microbiome. The latter can occur primarily or as a residual state following previous, clinically cured, enteric infections. The associated uncertainty regarding the assignment of etiological relevance in the case of diagnostic detections resulted in discouraging the use of highly sensitive diagnostic real-time multiplex PCR panels for the routine management of diarrhea patients in the up-to-date German guideline from November 2023 [8].

Suggestions for the inclusion of quantification or semi-quantification in the estimation of etiological relevance of detected stool pathogens have been made [5,6,7] but did not lead to the definition of broadly accepted interpretation standards so far, partly due to non-consistency of respective observations. Insofar, the definition of reliable parameters for the discrimination of etiological relevance or just co-existing colonization is still an unmet diagnostic need, in particular for high-endemicity settings where multiple pathogen detections are more the standard rather than the exception [4,5,6,7].

Indeed, there is presumably more than just quantity which decides on the etiological relevance of pathogens in the human gut. Epidemiological observations of a reduction in disease severity over time in the case of repeated exposure of an initially susceptible population to gastrointestinal pathogens in resource-poor settings point towards the development of acquired semi-immunity under such circumstances [9]. As early as in 1987, it was shown that in young children living in high-endemicity settings, rapid cycling of pathogen exposition, clearance and re-expositions is associated with waning disease severity of enteric campylobacteriosis, resulting in shifts from infections to carrier states [10,11]. Unfortunately, the specific nature of the associated semi-immunity is not yet entirely understood, although it is already harnessed in preventive medical applications. Oral vaccination against typhoid fever is such an example of induced short-term protection in the human gut. As expected, especially considering the type of application, a recent meta-analysis confirmed an association between the quantity of oral antigen exposure and the strength of associated immune protection after oral typhoid fever vaccination [12]. In the case of chronic parasite infections with enteric helminths, of note, 70% of reported pathogenicity was found to be associated with only 15% of infected individuals, in line with comparably well-characterized immunological adaptation processes, as summarized in a previous review [13].

Apart from semi-immunity mediated by adaptive immune responses, the composition of the gut microbiome is also believed to mitigate an enteric pathogen’s ability to show clinically apparent virulence. Under optimum conditions, even colonization resistance towards enteric pathogens can be mediated by favorable compositions of the gut microbiome, as suggested for both laboratory animals and humans [14,15]. And even below the microbiome level, specific co-occurrence patterns of enteric microorganisms have been suggested to alter the virulence of individual microorganisms in the human gut [16,17].

In spite of the above-mentioned challenges for the assignment of etiological relevance to enteric pathogen detections in stools samples from individuals from high-endemicity settings, tools for such assignments are nevertheless desirable to guide both prioritization of public health measures as well as individual treatment decisions. Collider bias assessment is such a tool for situations in which a defined condition can be “multi-realized” due to the differential influences of various causes leading to the same effect as reported by Tönnies and colleagues [18]. Translated to the here-described situation, chronic gastroenteric disorders of a likely infectious etiology in the Colombian indigenous people, later referred to as chronic gastroenteric disorders, can be caused by a variety of different microbial pathogens. Assuming that there is a single leading pathogen in each case of gastroenteric disorder rather than summative effects of multiple microorganisms, such leading pathogens should be negatively associated with each other within the population of indigenous individuals suffering from chronic gastroenteric disorder, in line with the premises of collider bias [18].

Consequently, we performed a collider bias analysis with the meta-data from previous surveillance studies on enteric microorganisms in Colombian indigenous individuals with chronic gastroenteric disorders [1,2,3]. The aim was the identification of potential leading pathogens based on negative associations pointing towards potential collider bias situations.

## 2. Materials and Methods

### 2.1. Study Design

The assessment was designed as an explorative, hypothesis-forming, retrospective study using meta-data from previous observational and surveillance assessments on microorganisms detected in stool samples of Colombian indigenous Wiwa and Kogui populations suffering from gastroenteric disorders.

### 2.2. Applied Datasets

Composite datasets were created based on the meta-data of historic surveillance assessments on Colombian indigenous Wiwa and Kogui populations from the years 2014, 2018 and 2021, as previously published [1,2,3]. These assessments included diagnostic stool results based on both microscopy and real-time PCR in varying compositions. Also, residual materials from the samples had been included in test evaluation assessments, the results of which were also used for the composite datasets. For the study, no patient-related information was included in the composite datasets derived from the meta-data of the previous assessments [1,2,3]. However, as documented in the previous works [1,2,3], the female percentage of the patients varied from 35.4% to 51.4% and the mean age varied between 24.5 and 26.5 years of the age over the assessed sub-populations.

### 2.3. Assumptions Regarding the Dataset

For the collider bias assessment, a number of premises were considered as guaranteed, thereby accepting associated simplifications. First of all, chronic gastroenteric disorders abundant in all participating individuals [1,2,3] were considered as a unique syndromic entity just showing gradual differences in its clinical manifestation. Second, variance in the diagnostic accuracy of the applied diagnostic approaches, as defined by sensitivity and specificity over the various included surveillance assessments, was not considered, resulting in simple binary coding of the diagnostic results for each parameter (0 = negative, 1 = positive). Available cycle threshold values (Ct-values) of real-time PCR as a means of semi-quantification were recorded in the datasets, but collider bias calculation is a qualitative approach.

Each microorganism that was detected at least once in the stool samples of the assessed Wiwa and Kogui participants was included in the assessment. This resulted in the exclusion of *Ancylostoma* spp., *Clostridioides difficile* and *Vibrio* spp. as parameters that were included in the real-time PCR-based screening scheme but never detected in any of the assessed individuals. Recorded microorganisms were included irrespective of whether likely etiological relevance was considered or not. This strategy resulted in the inclusion of a total of 32 parameters in the analysis, comprising 10 bacterial pathotypes, species or genera (*Aeromonas* spp., *Campylobacter* spp., enteroaggregative *Escherichia coli* (EAEC), enteropathogenic *Escherichia coli* (EPEC), enterotoxigenic *Escherichia coli* (ETEC), *Salmonella* spp., Shiga toxin-producing *Escherichia coli* (STEC), *Shigella* spp./enteroinvasive *Escherichia coli* (EIEC, not further discriminable based on the *ipaH* gene detected in real-time PCR), *Tropheryma whipplei* and *Yersinia* spp.), 11 protozoa (*Blastocystis* spp., *Cryptosporidium* spp., *Cyclospora* spp., *Dientamoeba fragilis*, *Entamoeba coli*, *Entamoeba bangladeshi*/*dispar*/*histolytica*/*moshkovskii* complex, *Entamoeba histolytica*, *Endolimax nana*, *Giardia duodenalis*, *Iodamoeba buetschlii* and *Pentatrichomonas hominis*), 8 helminths (*Ascaris* spp., *Enterobius vermicularis*, *Hymenolepis* spp., *Necator americanus*, *Schistosoma* spp., *Strongyloides* spp., *Taenia* spp. and *Trichuris* spp.) and the microsporidial genus *Encephalocytozoon* spp. as well as conidia and pseudoconidia as fungal elements.

Considering the assessed bacteria, only real-time PCR results were available from the surveillance assessments used for the composition of the datasets [1,2,3]. For technical reasons associated with the used real-time PCR assays (*eae* gene used for EPEC screening occurring in STEC as well), mono-infections with STEC were technically indistinguishable from double infections with STEC and EPEC. Considering the very high overall EPEC proportions in the study population, the double infection hypothesis was favored. In detail, from a range between 197 and 210 EPEC detections depending on the true proportion of double infections, the higher assumption of 210 EPEC detections was chosen (please also see Table 2 from the results chapter).

For the above-mentioned protozoa and helminths, either microscopic detections only or, if available, real-time PCR-based detection and/or confirmation were considered as positive results. One exemption was the discrimination of *Entamoeba histolytica* and *E. bangladeshi*/*dispar*/*histolytica*/*moshkovskii* complex, which are microscopically indistinguishable based on the morphology of their cysts. Accordingly, the diagnosis *E. histolytica* was considered in case of positive *E. histolytica*-specific real-time PCR only. As *E. dispar*-specific real-time PCR was not conducted, cyst detections matching positive *E. histolytica* real-time PCR results might have been derived from either the abundance of *E. histolytica* alone or the combination of *E. histolytica* and other species of the *E. bangladeshi*/*dispar*/*histolytica*/*moshkovskii* complex. Considering consistently very high cycle threshold (Ct) values in *E. histolytica*-specific real-time PCR indicative of very low pathogen densities below the microscopic detection threshold as well as high proportions of cyst detections but only very poor matching with *E. histolytica*-specific real-time PCR signals, cyst-detections were generally assigned to the *E. bangladeshi*/*dispar*/*histolytica*/*moshkovskii* complex, while potential co-infection including *E. histolytica* was assumed in a minority of 14 instances of microscopic cyst detections combined with positive *E. histolytica* PCR. Considering this overall low number of events of cyst detections matching with positive *E. histolytica*-specific real-time PCR (please also see Table 2 from the results chapter for details), the quantitative relevance of potentially associated failure was considered as negligible. Further noteworthy, hookworm eggs were generally attributed to *Necator americanus*. The reason is that no trace of abundance of *Ancylostoma* spp. DNA was detected in any of the samples of the assessed indigenous individuals. In two individual instances, in which atypically shaped hookworm eggs had been characterized as “*Unicinaria* spp.-like” by microscopists in combination with negative *Necator americanus*-specific real-time PCR signals, these findings were excluded as uncertain from further calculations. Similarly, three atypically shaped nematode eggs characterized as “*Tricocephalus* spp.-like” by microscopists and thus using the outdated term for “*Trichuris*” were excluded as uncertain findings due to negative *Trichuris* spp.-specific real-time PCR results in the same samples.

As no microsporidia-specific staining techniques had been used during the surveillance assessments [1,2,3], *Encephalocytozoon* spp. results were only considered in cases in which specific real-time PCR had been performed. Finally, an abundance or absence of fungal elements like conidia and pseudoconidia had only been recorded for a minority of samples from the datasets and so calculations were restricted to this minority.

### 2.4. Statistical Assumptions

The assignment of causal relationships in observational studies is affected by the problem of causal interference. Confounding—i.e., the interference of a “confounder” with both exposure and outcome of an observational study—can at least partially be corrected by applying various statistical methods, as detailed elsewhere [19,20,21]. Correction for confounding, however, can result in so-called “collider bias”, if “colliding” of at least two factors affects the outcome [18]. Directed acyclic graphs (DAGs), i.e., simple graphical representations of causal associations, are frequently applied to identify potential sources of collider bias without a need for a deeper understanding of the underlying calculations and to easier distinguish colliding from classic confounding [22,23]. For the particular example of gastroenteric pathogens leading to the outcome gastroenteric disorder, it is visualized in the DAG presented in Table 1 how a presumed confounder “pathogen 2” can contribute to the collider “combined pathogen effect” in individuals with the outcome “gastroenteric disorder” due to exposure to “pathogen 1”.

If, however, both “pathogen 1” and “pathogen 2” are by themselves causally associated with the outcome “gastroenteric disorder”, it is assumed that the outcome will be more frequently associated with the exposure of either one or the other than with a combination of both of them. This is because more permissions need to be fulfilled to achieve the latter, more complex situation of combined occurrence. Accordingly, multivariate statistical approaches such as (longitudinal) mediation, moderation and suppression analysis, as well as their complex interplay might be considered. The latter, as well as collider bias assessment, have hardly been statistically addressed in gastrointestinal research, for which reason the assessment of collider bias by odds ratio is considered an important starting point: a negative association between “pathogen 1” and “pathogen 2” should be assumed if they indeed are elements of a colliding. For the study presented here, this negative association was assessed by performing odds ratio analysis.

Generally, individually lacking diagnostic parameters were no criteria for the exclusion of a sample from analysis. For the odds ratio calculations for the various microorganisms, only samples that had indeed been assessed for both microorganisms were included, resulting in varying total numbers of assessed samples for the various odds ratio calculations. Odds ratios with two-sided *p*-values and 95% confidence intervals using Fisher’s exact test with Yates’ continuity correction and the approximation of Woolf were calculated applying the software GraphPad InStat version 3.06 (GraphPad Software, Inc., San Diego, CA, USA). Significance was accepted in the case of *p* < 0.05. Correction for multiple testing with techniques like the Bonferroni–Holmes approach [24] was not conducted in this hypothesis-forming explorative assessment. In line with the assumptions detailed above, potential collider bias [18] was assumed in the case of negative odds ratio observed for two different microbial parameters within the assessed population of Colombian indigenous individuals with chronic gastroenteric disorders.

### 2.5. Ethics

An ethical clearance for this statistical assessment was not required in line with German national law, because the assessments were not based on original research data involving humans or human samples but just on meta-data from previously published studies.

## 3. Results

### 3.1. Microbial Detections Derived from the Assessed Datasets

A total of 773 completely anonymized datasets with diagnostic results obtained with different stool samples from Colombian indigenous individuals with chronic gastroenteric disorders were analyzed. Proportions of detected bacteria within the datasets ranged from 0.6% to 37.2%. In proportional order of detection, EPEC were most frequently recorded, followed by *Campylobacter* spp., EAEC, ETEC, Shigella spp./EIEC, STEC, *Tropheryma whipplei*, *Salmonella* spp., *Aeromonas* spp. and *Yersinia* spp. In declining order of frequency, the protozoa *Giardia duodenalis*, *Blastocystis* spp., *Entamoeba coli*, *Endolimax nana*, *E. bangladeshi*/*dispar*/*histolytica*/*moshkovskii* complex, *Dientamoeba fragilis*, *Cyclospora* spp., *Entamoeba histolytica*, *Iodamoeba buetschlii*, *Cryptosporidium* spp. and *Pentatrichomonas hominis*, as well as the helminths *Trichuris* spp., *Ascaris* spp., *Hymenolepis* spp., *Necator americanus*, *Strongyloides* spp., *Taenia* spp., *Hymenolepis* spp. and *Schistosoma* spp., were found. Only a few microsporidia were observed, and from the microscopically detected fungal elements, conidia were slightly more frequent than pseudoconidia. Details are provided in Table 2, including descriptive semi-quantification based on cycle threshold (Ct) values of real-time PCR. Shortly summarized, intermediate target DNA quantities corresponding to Ct values between 25 and 35 were recorded for most of the real-time PCR-assessed parameters. However, for *Aeromonas* spp., *Cyclospora* spp., *Entamoeba histolytica* and *Encephalocytozoon* spp., only traces of specific pathogen-DNA were observed in the stool samples, as indicated by Ct values > 35.

**Table 2:** Summarized presentation of microorganism-specific datasets obtained from the chosen studies and included in the calculations.

Microorganism	Detections n/n (%)	Number n of Microscopy-Based Detections	Number n of Molecular Confirmed Detections	Mean Value of Recorded Cycle Threshold (*Ct*) Values of Real-Time PCR (±Standard Deviation), (Number of Recorded *Ct* Values)
Bacteria				
*Aeromonas* spp.	5/287 (1.7%)	n.a.	5	43.2 (±1.6), (5)
*Campylobacter* spp.	231/708 (32.6%)	n.a.	231	30.4 (±5.5), (174)
Enteroaggregative *Escherichia coli* (EAEC)	177/565 (31.3%)	n.a.	177	27.8 (±5.5), (177)
Enteropathogenic *Escherichia coli* (EPEC)	210/565 (37.2%)	n.a.	210	27.6% (±5.5), (208)
Enterotoxigenic *Escherichia coli* (ETEC)	137/565 (24.2%)	n.a.	137	27.0 (±6.1), (137)
*Salmonella* spp.	15/714 (2.1%)	n.a.	15	31.0 (±5.7), (10)
Shiga toxin-producing *Escherichia coli* (STEC)	20/128 (15.6%)	n.a.	20	23.6 (±2.1), (20)
*Shigella* spp./enteroinvasive *Escherichia coli*	120/708 (16.9%)	n.a.	120	30.0 (±6.0), (98)
*Tropheryma whipplei*	28/389 (7.2%)	n.a.	28	30.6 (±3.0), (28)
*Yersinia* spp.	4/708 (0.6%)	n.a.	4	n.d. (n.a.), (0)
Protozoa				
*Blastocystis* spp.	391/773 (50.6%)	128	263	30.5 (±4.3), (263)
*Cryptosporidium* spp.	17/773 (2.2%)	0	17	34.6 (±4.6), (12)
*Cyclospora* spp.	72/773 (9.3%)	0	72	36.0 (±5.6), (57)
*Dientamoeba fragilis*	75/773 (9.7%)	0	75	29.5 (7.1), (75)
*Entamoeba coli*	227/729 (31.1%)	227	0	n.a.
*Entamoeba bangladeshi*/*7íspar*/*histolytica*/*moshkovskii* complex	118/730 (16.2%)	118	0	n.a.
*Entamoeba histolytica*	56/707 (7.9%)	n.a.	56	40.0 (±5.0), (47)
*Endolimax nana*	216/730 (29.6%)	216	0	n.a.
*Giardia duodenalis*	402/773 (52.0%)	54	348	30.0 (±5.1), (218)
*Iodamoeba buetschlii*	55/730 (7.5%)	55	0	n.a.
*Pentatrichomonas hominis*	3/729 (0.4%)	3	0	n.a.
Helminths				
*Ascaris* spp.	130/773 (16.8%)	23	107	32.0 (±4.0), (84)
*Enterobius vermicularis*	31/773 (4.0%)	14	17	32.0 (±3.5), (17)
*Hymenolepis* spp.	126/773 (16.3%)	10	116	30.0 (±4.6), (116)
*Necator americanus*	86/773 (11.1%)	4	82	34.0 (±3.3), (57)
*Schistosoma* spp.	4/730 (0.5)	0	4	35.0 (±1.4), (4)
*Strongyloides* spp.	39/773 (5.0%)	0	39	35.0 (±4.0), (34)
*Taenia* spp.	33/773 (4.3%)	0	33	30.8 (±4.1), (33)
*Trichuris* spp.	158/773 (20.4%)	14	144	29.4 (±3.3), (144)
Microsporidia				
*Encephalocytozoon* spp.	7/287 (2.4%)	0	7	36.1 (±1.8), (7)
Fungi				
Conidia	41/245 (16.7%)	41	0	n.a.
Pseudoconidia	27/245 (11.0%)	27	0	n.a.

n.a. = not applicable, n.d. = not documented.

### 3.2. Proportions and Patterns of Positive and Negative Associations Observed for the Assessed Microorganisms

As visualized in Table 3, Table 4, Table 5 and Table 6 below, positive associations between assessed microorganisms, as indicated by statistically significant odds ratios of >1 (n = 88), were much more frequent than negative associations, potentially indicating collider bias events, as suggested by statistically significant odds ratios of <1 (n = 14). Details of the odds ratio calculations are presented in Appendix A (Tables A1–A470). Of note, odds ratio calculation attempts leading to non-conclusive results due to more than one zero per row or column are not shown in Appendix A.

Focusing on the few negative associations, enteroaggregative *E. coli* showed the most respective signals (n = 6), followed by *Blastocystis* spp. (n = 3) (Table 3, Table 4 and Table 6). For all other assessed microorganisms, between 0 and 2 negative associations were recorded. Even more and as detailed in Table 4, the most negative associations comprised microorganisms that are commonly considered as enteric commensals rather than pathogens.

As detailed in Table 3 and Table 5 and additionally visualized in Table 6, positive associations were much more commonly observed. Two-digit numbers of positive associations within the study population were recorded for no less than 5 assessed microorganisms, comprising *Trichuris* spp. (n = 16), *D. fragilis* (n = 15), *Shigella* spp./EIEC (n = 12), *Ascaris* spp. (n = 11) and *Blastocystis* spp. (n = 10) in descending order of frequency. With the exemption of *Shigella* spp./EIEC, all genera and species with such high numbers of recorded positive associations were protozoan or helminth parasites.

## 4. Discussion

This study was conducted as a hypothesis-forming explorative assessment with the aim of identifying potential etiological relevance of detected microorganisms in stool samples from Colombian indigenous individuals with chronic gastroenteric disorders of a likely infectious etiology by applying collider bias calculation. The performed analyses led to a number of results.

First of all, statistically significant odds ratios of <1, indicating negative associations potentially pointing towards collider bias within the study population, were rare events compared to observed positive associations. Even beyond quantitative rarity, the observations are difficult to interpret because microorganisms with unlikely relevance as an independent cause of infectious gastroenteritis were primarily affected. In our assessment, enteroaggregative *E. coli* (EAEC) was most frequently negatively associated with other recorded microorganisms. As known from previous studies in resource-limited high-endemicity settings like tropical Tanzania [7], EAEC is similarly frequent in patients with diarrhea, such as in asymptomatic individuals. And even for international travelers, in whom semi-immunity induced by frequent re-exposure to the same pathogen is quite unlikely, EAEC was not found to be significantly associated with infectious gastroenteritis as a primary pathogen in a recent study [25]. Insofar, it seems unlikely that the observed negative associations truly prove independent etiological relevance of the EAEC detections.

*Blastocystis* spp. was the second microorganism most frequently associated with significant odds ratios of <1 in the present assessment. Although some authors postulate a potential etiological relevance of *Blastocystis* spp. in human gastroenteritis depending on factors like microbiome composition and immunological situation [26,27,28,29], this opinion is not generally accepted in the scientific world. Also, *Blastocystis* spp. was found to be more frequently involved in positive than in negative associations, and so one might speculate that previous assumptions on hypothetic etiological relevance of this microorganism in the human gut might in fact be due to co-occurring other infections.

At least two negative associations were found for *Shigella* spp./EIEC detections. Even in resource-limited high-endemicity settings, several previous studies provided epidemiological evidence of the relevance of *Shigella* spp./EIEC as a causative agent of infectious gastroenteritis in such areas [5,6,7,16,30]. In one of those previous assessments, even a negative association with norovirus as a potential hint for collider bias was suggested [25]. In the study presented here, however, negative associations affected the protozoa *Entamoeba coli* and *Iodamoeba buetschlii*, which are usually considered as harmless colonizers indicating poor hygiene conditions rather than relevant pathogens. Also, identical quantities of negative associations were recorded for other apathogenic protozoa such as *Endolimax nana* and *Entamoeba coli* and even for detected fungal conidia, suggesting that the relevance of the observation should not be overestimated. This is even more considerable keeping in mind the high number of positive associations observed for *Shigella* spp./EIEC in the here-presented study as well. Insofar, the two negative associations found for *Shigella* spp./EIEC detections poorly confirm any independent etiological relevance of this microorganism in the assessed Colombian indigenous individuals.

Apart from *Shigella* spp./EIEC, positive associations were most frequently observed for enteric helminths like *Trichuris* spp. and *Ascaris* spp., as well as for protozoa of questionable etiological relevance like *Blastocystis* spp. and *D. fragilis*. The latter, in particular, is well in line with a previous modelling with samples from a smaller number of Colombian indigenous individuals suggesting mediating effects of microbial clusters containing these microorganisms on the likelihood of abundance of other enteric microorganisms, as reported by our group [31]. Such assessments, however, would be beyond the scope of the here-presented collider-bias assessment and might be considered subjects for future studies. Although the observed positive associations from this study might point towards growth-facilitating effects of one microorganism on another, alternative explanations are nevertheless not ruled out. One similarly likely alternative explanation could be shared modes of transmission due to common habits of subgroups within the assessed study population. The here-presented study, based on calculations with anonymized datasets without inclusion of patient parameters, was not designed to decide on this question.

To summarize, this study did not indicate collider bias pointing towards a likely pronounced etiological relevance of specific single microorganisms as an explanation for the chronic gastroenteric disorders shared by the assessed indigenous individuals. Instead, there were numerous hints for positive associations, suggesting mutual growth support of various microorganisms detected within the assessed stool samples, which is in line with previous observations [16,17]. Insofar, we feel justified to postulate based on our results that collider bias assessment, at least on an individual pathogen level, is a too-simple approach to conclude on complex associations like identification of causal relationships between pathogen exposure and gastroenteric disorders in individuals threatened by constant exposure in high-endemicity settings for gastroenteric pathogens. Screening for these more complex associations based on other biostatistical approaches, as recently performed [31], should be considered for future analyses.

This study has a few limitations. First of all, and as stated in the methods chapter, its assessment has a number of premises which all need to hold true if the results of the assessment shall be considered conclusive. However, even the main assumption that all study participants basically shared the same syndromic entity of chronic gastroenteric disorders with only gradual differences in symptom expression is associated with uncertainty. In fact, the restricted logistic options during the studies providing the data [1,2,3] make the definition of the disease entity vague and only syndromically describable. Second, differences in diagnostic accuracy of applied diagnostic strategies over the included surveillance assessments [1,2,3] were considered negligible for this analysis. However, this is not necessarily the case, especially in situations in which results from highly sensitive real-time PCR and investigator-dependent microscopy were merged for the analyses. Third, the analyses included considerable numbers of parameters, but not necessarily all pathogens potentially occurring in the human gut with a particular neglect of viruses and also not other factors like chronic inflammatory or functional gastrointestinal disorders. Only parameters previously assessed in the quoted surveillance studies [1,2,3] could be used for the calculations. Fourth, the retrospective design and the associated need to work with previously obtained diagnostic data did not allow a case number calculation-based assessment. Accordingly, the study was considered as a hypothesis-forming analysis only.

## 5. Conclusions

In spite of the limitations stated above, the study results suggested that single specific pathogens did not dominate the etiology of the medical condition of chronic gastroenteric disorder, as observed in the previously assessed Colombian indigenous populations [1,2,3]. Instead, and in line with previous observations with the same individuals [31], complex interactions seem to have played a role, potentially including whole microbial clusters and summative effects of co-occurring microorganisms. Future assessments including other biostatistical algorithms should be considered to verify or falsify this hypothesis. In particular, the terms “confounder” as well as “collider” correspond to the statistical concepts of moderation and mediation [18]. Using statistical multivariate modeling techniques associated with these concepts, associations observed in this study may be mathematically elaborated, thus paving the way for individually tailored therapeutic interventions. Such approaches, however, would be beyond the scope of the here-presented hypothesis-forming analysis and should be considered for future follow-up assessments.

## Figures and Tables

**Table 1:** Directed acyclic graph visualizing how exposure to pathogen 1 and apparent “confounding” by pathogen 2 might indeed result in “colliding” into the factor combined pathogen effect in Colombian indigenous individuals with gastroenteric disorder.

Gastroenteric pathogen 1 (exposure)			Gastroenteric pathogen 2 (confounder)	
↓	↓		↓	↓
↓	Combined effect of pathogen 1 and 2 (collider)			↓
↓	↓			↓
Gastroenteric disorder (outcome)				

**Table 3:** Numbers of observed positive associations (as indicated by statistically significant odds ratios of >1) and negative associations (as indicated by significant odds ratios of <1, suggesting potential collider bias) for the various included microorganisms (in alphabetical order).

Total Number (n)	Positive Associations Observed for the Respective Microorganisms	Negative Associations Observed for the Respective Microorganisms
0	*Encephalocytozoon* spp., *Pentatrichomonas hominis*, *Tropheryma whipplei*, *Schistosoma* spp.	*Aeromonas* spp., *Ascaris* spp., *Campylobacter* spp., *Cryptosporidium* spp., *Cyclospora* spp., *Encephalocytozoon* spp., *Entamoeba histolytica*, *Enterobius vermicularis*, ETEC, *Necator americanus*, *Pentatrichomonas hominis*, pseudoconidia, *Schistosoma* spp., STEC, *Strongyloides* spp, *Taenia* spp., *Trichuris* spp., *Tropheryma whipplei*, *Yersinia* spp.
1	Aeromonas spp., STEC, *Yersinia* spp.	*Dientamoeba fragilis*, *Giardia duodenalis*, *Hymenolepis* spp., *Salmonella* spp.
2	Conidia, pseudoconidia	Conidia, *Endolimax nana*, *Entamoeba coli*, *E. bangladeshi*/*dispar*/*histolytica*/*moshkovskii* complex, EPEC, *Iodamoeba buetschlii*, *Shigella* spp./EIEC
3	*Salmonella* spp., *Enterobius vermicularis*, *Hymenolepis* spp.	*Blastocystis* spp.
4	*Cryptosporidium* spp., EAEC, *Iodamoeba buetschlii*	-
5	EPEC	-
6	*Endolimax nana*, *Entamoeba histolytica*, *Giardia duodenalis*, *Necator americanus*	EAEC
7	*Cyclospora* spp., ETEC, *Taenia* spp.	-
8	*Entamoeba bangladeshi*/*dispar*/*histolytica*/*moshkovskii* complex, *Strongyloides* spp.	-
9	*Campylobacter* spp., *Entamoeba coli*,	-
10	*Blastocystis* spp.	-
11	*Ascaris* spp.	-
12	*Shigella* spp./EIEC	-
13	-	-
14	-	-
15	*Dientamoeba fragilis*	-
16	*Trichuris* spp.	-

EAEC = enteroaggregative *Escherichia coli*, EIEC = enteroinvasive *Escherichia coli*, EPEC = enteropathogenic *Escherichia coli*, ETEC = enterotoxigenic *Escherichia coli*, STEC = Shiga toxin-producing *Escherichia coli*.

**Table 4:** Associations of microorganisms characterized by statistically significant odds ratios of <1 (indicative of collider bias).

Microorganism	Associations with Odds Ratios < 1
Bacteria	
*Aeromonas* spp.	-
*Campylobacter* spp.	-
Enteroaggregative *Escherichia coli* (EAEC)	*Blastocystis* spp., *Dientamoeba fragilis*, *Entamoeba coli*, *E. bangladeshi*/*dispar*/*histolytica*/*moshkovskii* complex, *Endolimax nana*, *Iodamoeba buetschlii*
Enteropathogenic *Escherichia coli* (EPEC)	*Campylobacter* spp., *Giardia duodenalis*
Enterotoxigenic *Escherichia coli* (ETEC)	-
*Salmonella* spp.	*Endolimax nana*
Shiga toxin-producing *Escherichia coli* (STEC)	-
*Shigella* spp./enteroinvasive *Escherichia coli* (EIEC)	*Entamoeba coli*, *Iodamoeba buetschlii*
*Tropheryma whipplei*	-
*Yersinia* spp.	-
Protozoa	
*Blastocystis* spp.	*Hymenolepis* spp., EAEC, conidia
*Cryptosporidium* spp.	-
*Cyclospora* spp.	-
*Dientamoeba fragilis*	EAEC
*Endolimax nana*	*Salmonella* spp., EAEC
*Entamoeba coli*	*Shigella* spp., EAEC
*Entamoeba bangladeshi*/*dispar*/*histolytica*/*moshkovskii* complex	EAEC, conidia
*Entamoeba histolytica*	-
*Giardia duodenalis*	EPEC
*Iodamoeba buetschlii*	*Shigella* spp., EAEC
*Pentatrichomonas hominis*	-
Helminths	
*Ascaris* spp.	-
*Enterobius vermicularis*	-
*Hymenolepis* spp.	*Blastocystis* spp.
*Necator americanus*	-
*Schistosoma* spp.	-
*Strongyloides* spp.	-
*Taenia* spp.	-
*Trichuris* spp.	-
Microsporidia	
*Encephalocytozoon* spp.	-
Fungi	
Conidia	*Blastocystis* spp., *Entamoeba bangladeshi*/*dispar*/*histolytica*/*moshkovskii* complex
Pseudoconidia	-

**Table 5:** Associations of microorganisms characterized by statistically significant odds ratios of >1.

Microorganism	Associations with Odds Ratios > 1
Bacteria	
*Aeromonas* spp.	*Cyclospora* spp.
*Campylobacter* spp.	*Shigella* spp., ETEC, *Blastocystis* spp., *Dientamoeba fragilis, Giardia duodenalis*, *Ascaris* spp., *Hymenolepis* spp., *Taenia* spp., *Trichuris* spp.
Enteroaggregative *Escherichia coli* (EAEC)	*Salmonella* spp., *Shigella* spp., EPEC, ETEC
Enteropathogenic *Escherichia coli* (EPEC)	*Salmonella* spp., ETEC, EAEC, STEC, *Dientamoeba fragilis*
Enterotoxigenic *Escherichia coli* (ETEC)	*Salmonella* spp., *Shigella* spp., *Campylobacter* spp., *Necator americanus*, *Trichuris* spp., EPEC, EAEC
*Salmonella* spp.	EPEC, ETEC, EAEC
Shiga toxin-producing *Escherichia coli* (STEC)	EPEC
*Shigella* spp./enteroinvasive *Escherichia coli* (EIEC)	*Campylobacter* spp., ETEC, EAEC, *Yersinia* spp., *Cryptosporidium* spp., *Dientamoeba fragilis*, *Entamoeba histolytica, Ascaris* spp., *Necator americanus*, *Strongyloides* spp., *Taenia* spp., *Trichuris* spp.
*Tropheryma whipplei*	-
*Yersinia* spp.	*Shigella* spp.
Protozoa	
*Blastocystis* spp.	*Campylobacter* spp., *Giardia duodenalis*, *Ascaris* spp., *Strongyloides* spp., *Trichuris* spp., *Dientamoeba fragilis*, *Entamoeba coli*, *E. bangladeshi*/*dispar*/*histolytica*/*moshkovskii* complex, *Endolimax nana*, *Iodamoeba buetschlii*
*Cryptosporidium* spp.	*Shigella* spp., *Entamoeba histolytica*, *Dientamoeba fragilis*, *Trichuris* spp.
*Cyclospora* spp.	*Giardia duodenalis*, *Aeromonas* spp., *Dientamoeba fragilis*, *Entamoeba coli*, *Endolimax nana*, *Ascaris* spp., *Trichuris* spp.
*Dientamoeba fragilis*	*Shigella* spp., *Campylobacter* spp., *Giardia duodenalis*, *Entamoeba histolytica*, *Cyclospora* spp., *Cryptosporidium* spp., *Ascaris* spp., *Necator americanus*, *Strongyloides* spp., *Taenia* spp., EPEC, *Blastocystis* spp., *Entamoeba coli*, *Entamoeba bangladeshi*/*dispar*/*histolytica*/*moshkovskii* complex, *Endolimax nana*
*Endolimax nana*	*Cyclospora* spp., *Dientamoeba fragilis*, *Blastocystis* spp., *Entamoeba coli*, *Entamoeba bangladeshi*/*dispar*/*histolytica*/*moshkovskii* complex, *Iodamoeba buetschlii*
*Entamoeba coli*	*Cyclospora* spp., *Strongyloides* spp., *Taenia* spp., *Trichuris* spp., *Dientamoeba fragilis*, *Blastocystis* spp., *Endolimax nana*, *Entamoeba bangladeshi*/*dispar*/*histolytica*/*moshkovskii* complex, *Iodamoeba buetschlii*
*Entamoeba bangladeshi*/*dispar*/*histolytica*/*moshkovskii* complex	*Ascaris* spp., *Strongyloides* spp., *Trichuris* spp., *Dientamoeba fragilis*, *Blastocystis* spp., *Endolimax nana*, *Entamoeba coli*, *Iodamoeba buetschlii*
*Entamoeba histolytica*	*Shigella* spp., *Cryptosporidium* spp., *Dientamoeba fragilis, Ascaris* spp., *Strongyloides* spp., *Trichuris* spp.
*Giardia duodenalis*	*Campylobacter* spp., *Blastocystis* spp., *Cyclospora* spp., *Dientamoeba fragilis*, *Hymenolepis* spp., *Trichuris* spp.
*Iodamoeba buetschlii*	*Blastocystis* spp., *Endolimax nana*, *Entamoeba coli*, *Entamoeba bangladeshi*/*dispar*/*histolytica*/*moshkovskii* complex
*Pentatrichomonas hominis*	-
Helminths	
*Ascaris* spp.	*Shigella* spp., *Campylobacter* spp., *Entamoeba histolytica*, *Cyclospora* spp., *Necator americanus*, *Strongyloides* spp., *Trichuris* spp., *Enterobius vermicularis*, *Dientamoeba fragilis*, *Blastocystis* spp., *Entamoeba bangladeshi*/*dispar*/*histolytica*/*moshkovskii* complex
*Enterobius vermicularis*	*Ascaris* spp., *Trichuris* spp., conidia
*Hymenolepis* spp.	*Campylobacter* spp., *Giardia duodenalis*, *Trichuris* spp.
*Necator americanus*	*Shigella* spp., *Ascaris* spp., *Strongyloides* spp., *Trichuris* spp., ETEC, *Dientamoeba fragilis*
*Schistosoma* spp.	-
*Strongyloides* spp.	*Shigella* spp., *Ascaris* spp., *Necator americanus*, *Blastocystis* spp., *Dientamoeba fragilis*, *Entamoeba coli*, *Entamoeba bangladeshi*/*dispar*/*histolytica*/*moshkovskii* complex, *Trichuris* spp.
*Taenia* spp.	*Shigella* spp., *Campylobacter* spp., *Entamoeba histolytica*, *Dientamoeba fragilis*, *Entamoeba coli*, *Trichuris* spp., pseudoconidia
*Trichuris* spp.	*Shigella* spp., *Campylobacter* spp., *Giardia duodenalis*, *Entamoeba histolytica*, *Cyclospora* spp., *Cryptosporidium* spp., *Ascaris* spp., *Necator americanus*, *Strongyloides* spp., *Taenia* spp., *Hymenolepis* spp., ETEC, *Blastocystis* spp., *Dientamoeba fragilis*, *Entamoeba bangladeshi*/*dispar*/*histolytica*/*moshkovskii* complex, *Enterobius vermicularis*
Microsporidia	
*Encephalocytozoon* spp.	-
Fungi	
Conidia	*Enterobius vermicularis*, pseudoconidia
Pseudoconidia	*Taenia* spp., conidia

**Table 6:** Cross-tabulation of the assessed parameters coded by numbers to visualize associations as indicated by odds ratios. Color code green = significant odds ratio of >1, indicating a positive association. Color code red = significant odds ratio of <1, indicating a negative association and thus, a potential collider bias. Color code blue = lacking significance. Color code black = odds ratio not estimable based on available data. X- and Y-axis are labeled with the coded microorganisms assessed.

	01	02	03	04	05	06	07	08	09	10	11	12	13	14	15	16	17	18	19	20	21	22	23	24	25	26	27	28	29	30	31
02																															
03																															
04																															
05																															
06																															
07																															
08																															
09																															
10																															
11																															
12																															
13																															
14																															
15																															
16																															
17																															
18																															
19																															
20																															
21																															
22																															
23																															
24																															
25																															
26																															
27																															
28																															
29																															
30																															
31																															
32																															

01 = *Salmonella* spp., 02 = *Shigella* spp./enteroinvasive *Escherichia coli*, 03 = *Campylobacter* spp., 04 = *Yersinia* spp., 05 = *Entamoeba histolytica*, 06 = *Giardia duodenalis*, 07 = *Cyclospora cayetanensis*, 08 = *Cryptosporidium* spp., 09 = *Ascaris* spp., 10 = *Necator americanus*, 11 = *Strongyloides* spp., 12 = *Taenia* spp., 13 = *Hymenolepis* spp., 14 = *Trichuris* spp., 15 = *Schistosoma* spp., 16 = *Enterobius vermicularis*, 17 = *Tropheryma whipplei*, 18 = enteropathogenic *Escherichia coli*, 19 = enterotoxigenic *Escherichia coli*, 20 = enteroaggregative *Escherichia coli*, 21 = Shiga toxin-producing *Escherichia coli*, 22 = *Dientamoeba fragilis*, 23 = *Blastocystis* spp., 24 = *Endolimax nana*, 25 = *Entamoeba coli*, 26 = *Entamoeba bangladeshi*/*dispar*/*histolytica*/*moshkovskii* complex, 27 = *Iodamoeba buetschlii*, 28 = *Pentatrichomonas hominis*, 29 = *Encephalocytozoon* spp., 30 = *Aeromonas* spp., 31 = conidia, 32 = pseudoconidia.

## Data Availability

All relevant information is included in the manuscript or its appendix tables. The raw data supporting the conclusions of this article will be made available by the authors on request.

## Appendix A

Odds ratio calculations for the individual parameters are shown in Tables A1–A470. Tables for which odds ratio was not estimable due to more than one zero per line or column are not provided. As a simplification, *Entamoeba bangladeshi*/*dispar*/*histolytica*/*moshkovskii* complex (indicating samples with suspicious cysts but without PCR-based confirmation of *E. histolytica*) is summarized as *Entamoeba dispar* in the following.

**Table A1:** Odds ratio for the association of *Salmonella* spp. and *Shigella* spp.

	*Salmonella* spp.	Non-*Salmonella* spp.
*Shigella* spp.	6	114
Non-*Shigella* spp.	11	579
Odds ratio	95% confidence interval	Significance level P
2.770	1.004, 7.645	0.0513, not significant

**Table A2:** Odds ratio for the association of *Salmonella* spp. and *Campylobacter* spp.

	*Salmonella* spp.	Non-*Salmonella* spp.
*Campylobacter* spp.	3	228
Non-*Campylobacter* spp.	12	465
Odds ratio	95% confidence interval	Significance level P
0.5099	0.1424, 1.825	0.4074, not significant

**Table A3:** Odds ratio for the association of *Salmonella* spp. and *Yersinia* spp.

	*Salmonella* spp.	Non-*Salmonella* spp.
*Yersinia* spp.	0	4
Non-*Yersinia* spp.	15	689
Odds ratio	95% confidence interval	Significance level P
4.943	0.2547, 95.912	1.000, not significant

**Table A4:** Odds ratio for the association of *Salmonella* spp. and *Entamoeba histolytica*.

	*Salmonella* spp.	Non-*Salmonella* spp.
*Entamoeba histolytica*	2	54
Non-*Entamoeba histolytica*	13	638
Odds ratio	95% confidence interval	Significance level P
1.818	0.3996, 8.267	0.3362, not significant

**Table A5:** Odds ratio for the association of *Salmonella* spp. and *Giardia duodenalis*.

	*Salmonella* spp.	Non-*Salmonella* spp.
*Giardia duodenalis*	7	387
Non-*Giardia duodenalis*	8	311
Odds ratio	95% confidence interval	Significance level P
0.7032	0.2522, 1.961	0.6025, not significant

**Table A6:** Odds ratio for the association of *Salmonella* spp. and *Cyclospora* spp.

	*Salmonella* spp.	Non-*Salmonella* spp.
*Cyclospora* spp.	2	70
Non-*Cyclospora* spp.	13	629
Odds ratio	95% confidence interval	Significance level P
1.382	0.3056, 6.254	0.6566, not significant

**Table A7:** Odds ratio for the association of *Salmonella* spp. and *Cryptosporidium* spp.

	*Salmonella* spp.	Non-*Salmonella* spp.
*Cryptosporidium* spp.	0	17
Non-*Cryptosporidium* spp.	15	682
Odds ratio	95% confidence interval	Significance level P
1.258	0.07230, 21.892	1.0000, not significant

**Table A8:** Odds ratio for the association of *Salmonella* spp. and *Ascaris* spp.

	*Salmonella* spp.	Non-*Salmonella* spp.
*Ascaris* spp.	4	125
Non-*Ascaris* spp.	11	574
Odds ratio	95% confidence interval	Significance level P
1.670	0.5230, 5.331	0.3281, not significant

**Table A9:** Odds ratio for the association of *Salmonella* spp. and *Necator americanus*.

	*Salmonella* spp.	Non-*Salmonella* spp.
*Necator americanus*	1	85
Non-*Necator americanus*	14	614
Odds ratio	95% confidence interval	Significance level P
0.5160	0.06696, 3.976	1.0000, not significant

**Table A10:** Odds ratio for the association of *Salmonella* spp. and *Strongyloides* spp.

	*Salmonella* spp.	Non-*Salmonella* spp.
*Strongyloides* spp.	1	38
Non-*Strongyloides* spp.	14	661
Odds ratio	95% confidence interval	Significance level P
1.242	0.1591, 9.704	0.5731, not significant

**Table A11:** Odds ratio for the association of *Salmonella* spp. and *Taenia* spp.

	*Salmonella* spp.	Non-*Salmonella* spp.
*Taenia* spp.	1	32
Non-*Taenia* spp.	14	667
Odds ratio	95% confidence interval	Significance level P
1.489	0.1898, 11.682	0.5118, not significant

**Table A12:** Odds ratio for the association of *Salmonella* spp. and *Hymenolepis* spp.

	*Salmonella* spp.	Non-*Salmonella* spp.
*Hymenolepis* spp.	2	114
Non-*Hymenolepis* spp.	13	585
Odds ratio	95% confidence interval	Significance level P
0.7895	0.1757, 3.547	1.0000, not significant

**Table A13:** Odds ratio for the association of *Salmonella* spp. and *Trichuris* spp.

	*Salmonella* spp.	Non-*Salmonella* spp.
*Trichuris* spp.	1	156
Non-*Trichuris* spp.	14	543
Odds ratio	95% confidence interval	Significance level P
0.2486	0.03242, 1.906	0.2114, not significant

**Table A14:** Odds ratio for the association of *Salmonella* spp. and *Schistosoma* spp.

	*Salmonella* spp.	Non-*Salmonella* spp.
*Schistosoma* spp.	0	3
Non-*Schistosoma* spp.	15	654
Odds ratio	95% confidence interval	Significance level P
6.032	0.2984, 121.94	1.0000, not significant

**Table A15:** Odds ratio for the association of *Salmonella* spp. and *Enterobius vermicularis*.

	*Salmonella* spp.	Non-*Salmonella* spp.
*Enterobius vermicularis*	0	29
Non-*Enterobius vermicularis*	15	670
Odds ratio	95% confidence interval	Significance level P
0.7332	0.04280, 12.560	1.0000, not significant

**Table A16:** Odds ratio for the association of *Salmonella* spp. and *Tropheryma whipplei*.

	*Salmonella* spp.	Non-*Salmonella* spp.
*Tropheryma whipplei*	0	21
Non-*Tropheryma whipplei*	6	324
Odds ratio	95% confidence interval	Significance level P
1.161	0.06324, 21.314	1.0000, not significant

**Table A17:** Odds ratio for the association of *Salmonella* spp. and enteropathogenic *Escherichia coli*.

	*Salmonella* spp.	Non-*Salmonella* spp.
Enteropathogenic *Escherichia coli*	7	184
Non-enteropathogenic *Escherichia coli*	2	334
Odds ratio	95% confidence interval	Significance level P
6.353	1.306, 30.919	0.0132, significant

**Table A18:** Odds ratio for the association of *Salmonella* spp. and enterotoxigenic *Escherichia coli*.

	*Salmonella* spp.	Non-*Salmonella* spp.
Enterotoxigenic *Escherichia coli*	6	124
Non-enterotoxigenic *Escherichia coli*	3	394
Odds ratio	95% confidence interval	Significance level P
6.355	1.566, 25.793	0.0088, significant

**Table A19:** Odds ratio for the association of *Salmonella* spp. and enteroaggregative *Escherichia coli*.

	*Salmonella* spp.	Non-*Salmonella* spp.
Enteroaggregative *Escherichia coli*	7	162
Non-enteroaggregative *Escherichia coli*	2	356
Odds ratio	95% confidence interval	Significance level P
7.691	1.580, 37.446	0.0061, significant

**Table A20:** Odds ratio for the association of *Salmonella* spp. and Shiga toxin-producing *Escherichia coli*.

	*Salmonella* spp.	Non-*Salmonella* spp.
Shiga toxin-producing *Escherichia coli*	2	18
Non-Shiga toxin-producing *Escherichia coli*	4	104
Odds ratio	95% confidence interval	Significance level P
2.889	0.4921, 16.961	0.2361, not significant

**Table A21:** Odds ratio for the association of *Salmonella* spp. and *Dientamoeba fragilis*.

	*Salmonella* spp.	Non-*Salmonella* spp.
*Dientamoeba fragilis*	0	75
Non-*Dientamoeba fragilis*	15	624
Odds ratio	95% confidence interval	Significance level P
0.2668	0.01579, 4.508	0.3883, not significant

**Table A22:** Odds ratio for the association of *Salmonella* spp. and *Blastocystis* spp.

	*Salmonella* spp.	Non-*Salmonella* spp.
*Blastocystis* spp.	5	366
Non-*Blastocystis* spp.	10	333
Odds ratio	95% confidence interval	Significance level P
0.4549	0.1539, 1.345	0.1925, not significant

**Table A23:** Odds ratio for the association of *Salmonella* spp. and *Endolimax nana*.

	*Salmonella* spp.	Non-*Salmonella* spp.
*Endolimax nana*	1	198
Non-*Endolimax nana*	14	458
Odds ratio	95% confidence interval	Significance level P
0.1652	0.02157, 1.266	0.0491, significant

**Table A24:** Odds ratio for the association of *Salmonella* spp. and *Entamoeba coli*.

	*Salmonella* spp.	Non-*Salmonella* spp.
*Entamoeba coli*	4	203
Non-*Entamoeba coli*	11	452
Odds ratio	95% confidence interval	Significance level P
0.8097	0.2547, 2.574	1.0000, not significant

**Table A25:** Odds ratio for the association of *Salmonella* spp. and *Entamoeba dispar*.

	*Salmonella* spp.	Non-*Salmonella* spp.
*Entamoeba dispar*	0	111
Non-*Entamoeba dispar*	15	545
Odds ratio	95% confidence interval	Significance level P
0.1578	0.009367, 2.659	0.1494, not significant

**Table A26:** Odds ratio for the association of *Salmonella* spp. and *Iodamoeba buetschlii*.

	*Salmonella* spp.	Non-*Salmonella* spp.
*Iodamoeba buetschlii*	1	47
Non-*Iodamoeba buetschlii*	14	609
Odds ratio	95% confidence interval	Significance level P
0.9255	0.1190, 7.196	1.0000, not significant

**Table A27:** Odds ratio for the association of *Salmonella* spp. and *Pentatrichomonas hominis*.

	*Salmonella* spp.	Non-*Salmonella* spp.
*Pentatrichomonas hominis*	0	3
Non-*Pentatrichomonas hominis*	15	652
Odds ratio	95% confidence interval	Significance level P
6.014	0.2975, 121.57	1.0000, not significant

**Table A28:** Odds ratio for the association of *Salmonella* spp. and *Encephalocytozoon* spp.

	*Salmonella* spp.	Non-*Salmonella* spp.
*Encephalocytozoon* spp.	0	7
Non-*Encephalocytozoon* spp.	4	276
Odds ratio	95% confidence interval	Significance level P
4.096	0.2017, 83.201	1.0000, not significant

**Table A29:** Odds ratio for the association of *Salmonella* spp. and *Aeromonas* spp.

	*Salmonella* spp.	Non-*Salmonella* spp.
*Aeromonas* spp.	1	4
Non-*Aeromonas* spp.	3	279
Odds ratio	95% confidence interval	Significance level P
23.250	1.967, 274.77	0.0682, not significant

**Table A30:** Odds ratio for the association of *Salmonella* spp. and conidia.

	*Salmonella* spp.	Non-*Salmonella* spp.
Conidia	0	41
Non-conidia	4	200
Odds ratio	95% confidence interval	Significance level P
0.5368	0.02834, 10.170	1.0000, not significant

**Table A31:** Odds ratio for the association of *Salmonella* spp. and pseudoconidia.

	*Salmonella* spp.	Non-*Salmonella* spp.
Pseudoconidia	0	27
Non-pseudoconidia	4	214
Odds ratio	95% confidence interval	Significance level P
0.8667	0.04539, 16.547	1.0000, not significant

**Table A32:** Odds ratio for the association of *Shigella* spp. and *Campylobacter* spp.

	*Shigella* spp.	Non-*Shigella* spp.
*Campylobacter* spp.	62	169
Non-*Campylobacter* spp.	58	419
Odds ratio	95% confidence interval	Significance level P
2.650	1.776, 3.955	<0.0001, significant

**Table A33:** Odds ratio for the association of *Shigella* spp. and *Yersina* spp.

	*Shigella* spp.	Non-*Shigella* spp.
*Yersinia* spp.	3	1
Non-*Yersinia* spp.	117	587
Odds ratio	95% confidence interval	Significance level P
15.051	1.551, 146.04	0.0167, significant

**Table A34:** Odds ratio for the association of *Shigella* spp. and *Entamoeba histolytica*.

	*Shigella* spp.	Non-*Shigella* spp.
*Entamoeba histolytica*	17	39
Non-*Entamoeba histolytica*	103	548
Odds ratio	95% confidence interval	Significance level P
2.319	1.263, 4.257	0.0088, significant

**Table A35:** Odds ratio for the association of *Shigella* spp. and *Giardia duodenalis*.

	*Shigella* spp.	Non-*Shigella* spp.
*Giardia duodenalis*	64	331
Non-*Giardia duodenalis*	56	257
Odds ratio	95% confidence interval	Significance level P
0.8874	0.5984, 1.316	0.6142, not significant

**Table A36:** Odds ratio for the association of *Shigella* spp. and *Cyclospora* spp.

	*Shigella* spp.	Non-*Shigella* spp.
*Cyclospora* spp.	15	57
Non-*Cyclospora* spp.	105	531
Odds ratio	95% confidence interval	Significance level P
1.331	0.7259, 2.440	0.4062, not significant

**Table A37:** Odds ratio for the association of *Shigella* spp. and *Cryptosporidium* spp.

	*Shigella* spp.	Non-*Shigella* spp.
*Cryptosporidium* spp.	8	9
Non-*Cryptosporidium* spp.	112	579
Odds ratio	95% confidence interval	Significance level P
4.595	1.735, 12.169	0.0034, significant

**Table A38:** Odds ratio for the association of *Shigella* spp. and *Ascaris* spp.

	*Shigella* spp.	Non-*Shigella* spp.
*Ascaris* spp.	43	85
Non-*Ascaris* spp.	77	503
Odds ratio	95% confidence interval	Significance level P
3.305	2.132, 5.123	<0.0001, significant

**Table A39:** Odds ratio for the association of *Shigella* spp. and *Necator* spp.

	*Shigella* spp.	Non-*Shigella* spp.
*Necator* spp.	29	57
Non-*Necator* spp.	91	531
Odds ratio	95% confidence interval	Significance level P
2.969	1.802, 4.892	<0.0001, significant

**Table A40:** Odds ratio for the association of *Shigella* spp. and *Strongyloides* spp.

	*Shigella* spp.	Non-*Shigella* spp.
*Strongyloides* spp.	12	27
Non-*Strongyloides* spp.	108	561
Odds ratio	95% confidence interval	Significance level P
2.309	1.134, 4.699	0.0264, significant

**Table A41:** Odds ratio for the association of *Shigella* spp. and *Taenia* spp.

	*Shigella* spp.	Non-*Shigella* spp.
*Taenia* spp.	11	22
Non-*Taenia* spp.	109	566
Odds ratio	95% confidence interval	Significance level P
2.596	1.223, 5.510	0.0163, significant

**Table A42:** Odds ratio for the association of *Shigella* spp. and *Hymenolepis* spp.

	*Shigella* spp.	Non-*Shigella* spp.
*Hymenolepis* spp.	23	93
Non-*Hymenolepis* spp.	97	495
Odds ratio	95% confidence interval	Significance level P
1.262	0.7611, 2.093	0.4164, not significant

**Table A43:** Odds ratio for the association of *Shigella* spp. and *Trichuris* spp.

	*Shigella* spp.	Non-*Shigella* spp.
*Trichuris* spp.	67	87
Non-*Trichuris* spp.	53	501
Odds ratio	95% confidence interval	Significance level P
7.280	4.753, 11.149	<0.0001, significant

**Table A44:** Odds ratio for the association of *Shigella* spp. and *Schistosoma* spp.

	*Shigella* spp.	Non-*Shigella* spp.
*Schistosoma* spp.	1	2
Non-*Schistosoma* spp.	116	547
Odds ratio	95% confidence interval	Significance level P
2.358	0.2119, 26.235	0.4404, not significant

**Table A45:** Odds ratio for the association of *Shigella* spp. and *Enterobius vermicularis*.

	*Shigella* spp.	Non-*Shigella* spp.
*Enterobius vermicularis*	8	20
Non-*Enterobius vermicularis*	112	568
Odds ratio	95% confidence interval	Significance level P
2.029	0.8716, 4.721	0.1185, not significant

**Table A46:** Odds ratio for the association of *Shigella* spp. and *Tropheryma whipplei.*

	*Shigella* spp.	Non-*Shigella* spp.
*Tropheryma whipplei*	3	18
Non-*Tropheryma whipplei*	34	290
Odds ratio	95% confidence interval	Significance level P
1.422	0.3980, 5.078	0.4817, not significant

**Table A47:** Odds ratio for the association of *Shigella* spp. and enteropathogenic *Escherichia coli*.

	*Shigella* spp.	Non-*Shigella* spp.
Enteropathogenic *Escherichia coli*	39	149
Non-enteropathogenic *Escherichia coli*	54	279
Odds ratio	95% confidence interval	Significance level P
1.352	0.8559, 2.137	0.2334, not significant

**Table A48:** Odds ratio for the association of *Shigella* spp. and enterotoxigenic *Escherichia coli*.

	*Shigella* spp.	Non-*Shigella* spp.
Enterotoxigenic *Escherichia coli*	34	95
Non-enterotoxigenic *Escherichia coli*	59	333
Odds ratio	95% confidence interval	Significance level P
2.020	1.250, 3.264	0.0052, significant

**Table A49:** Odds ratio for the association of *Shigella* spp. and enteroaggregative *Escherichia coli*.

	*Shigella* spp.	Non-*Shigella* spp.
Enteroaggregative *Escherichia coli*	42	125
Non-enteroaggregative *Escherichia coli*	51	303
Odds ratio	95% confidence interval	Significance level P
1.996	1.262, 3.158	0.0046, significant

**Table A50:** Odds ratio for the association of *Shigella* spp. and Shiga toxin-producing *Escherichia coli*.

	*Shigella* spp.	Non-*Shigella* spp.
Shiga toxin-producing *Escherichia coli*	5	15
Non-Shiga toxin-producing *Escherichia coli*	28	80
Odds ratio	95% confidence interval	Significance level P
0.9524	0.3170, 2.861	1.0000, not significant

**Table A51:** Odds ratio for the association of *Shigella* spp. and *Dientamoeba fragilis*.

	*Shigella* spp.	Non-*Shigella* spp.
*Dientamoeba fragilis*	23	52
Non-*Dientamoeba fragilis*	97	536
Odds ratio	95% confidence interval	Significance level P
2.444	1.429, 4.179	0.0017, significant

**Table A52:** Odds ratio for the association of *Shigella* spp. and *Blastocystis* spp.

	*Shigella* spp.	Non-*Shigella* spp.
*Blastocystis* spp.	58	311
Non-*Blastocystis* spp.	62	277
Odds ratio	95% confidence interval	Significance level P
0.8332	0.5624, 1.234	0.3688, not significant

**Table A53:** Odds ratio for the association of *Shigella* spp. and *Endolimax nana*.

	*Shigella* spp.	Non-*Shigella* spp.
*Endolimax nana*	29	169
Non-*Endolimax nana*	87	380
Odds ratio	95% confidence interval	Significance level P
0.7495	0.4742, 1.185	0.2636, not significant

**Table A54:** Odds ratio for the association of *Shigella* spp. and *Endamoeba coli*.

	*Shigella* spp.	Non-*Shigella* spp.
*Endamoeba coli*	14	190
Non-*Endamoeba coli*	92	368
Odds ratio	95% confidence interval	Significance level P
0.2947	0.1635, 0.5312	<0.0001, significant

**Table A55:** Odds ratio for the association of *Shigella* spp. and *Endamoeba dispar.*

	*Shigella* spp.	Non-*Shigella* spp.
*Endamoeba dispar* spp.	17	94
Non-*Endamoeba dispar*.	99	455
Odds ratio	95% confidence interval	Significance level P
0.8312	0.4745, 1.456	0.5850, not significant

**Table A56:** Odds ratio for the association of *Shigella* spp. and *Iodamoeba buetschlii*.

	*Shigella* spp.	Non-*Shigella* spp.
*Iodamoeba buetschlii*	3	43
Non-*Iodamoeba buetschlii*	113	506
Odds ratio	95% confidence interval	Significance level P
0.3124	0.09520, 1.025	0.0436, significant

**Table A57:** Odds ratio for the association of *Shigella* spp. and *Pentatrichomonas hominis*.

	*Shigella* spp.	Non-*Shigella* spp.
*Pentatrichomonas hominis*	0	3
Non-*Pentatrichomonas hominis*	115	546
Odds ratio	95% confidence interval	Significance level P
0.6759	0.03465, 13.185	1.0000, not significant

**Table A58:** Odds ratio for the association of *Shigella* spp. and *Encephalocytozoon* spp.

	*Shigella* spp.	Non-*Shigella* spp.
*Encephalocytozoon* spp.	3	4
Non-*Encephalocytozoon* spp.	61	219
Odds ratio	95% confidence interval	Significance level P
2.693	0.5866, 12.360	0.1875, not significant

**Table A59:** Odds ratio for the association of *Shigella* spp. and *Aeromonas* spp.

	*Shigella* spp.	Non-*Shigella* spp.
*Aeromonas* spp.	3	2
Non-*Aeromonas* spp.	61	221
Odds ratio	95% confidence interval	Significance level P
5.434	0.8876, 33.272	0.0754, not significant

**Table A60:** Odds ratio for the association of *Shigella* spp. and conidia.

	*Shigella* spp.	Non-*Shigella* spp.
Conidia	9	32
Non-conidia	52	152
Odds ratio	95% confidence interval	Significance level P
0.8221	0.3679, 1.837	0.6969, not significant

**Table A61:** Odds ratio for the association of *Shigella* spp. and pseudoconidia.

	*Shigella* spp.	Non-*Shigella* spp.
Pseudoconidia	4	23
Non-pseudoconidia	57	161
Odds ratio	95% confidence interval	Significance level P
0.4912	0.1628, 1.482	0.2440, not significant

**Table A62:** Odds ratio for the association of *Campylobacter* spp. and *Yersinia* spp.

	*Campylobacter* spp.	Non-*Campylobacter* spp.
*Yersinia* spp.	2	2
Non-*Yersinia* spp.	229	475
Odds ratio	95% confidence interval	Significance level P
2.074	0.2902, 14.826	0.6000, not significant

**Table A63:** Odds ratio for the association of *Campylobacter* spp. and *Entamoeba histolytica.*

	*Campylobacter* spp.	Non-*Campylobacter* spp.
*Entamoeba histolytica*	23	33
Non-*Entamoeba histolytica*	208	443
Odds ratio	95% confidence interval	Significance level P
1.484	0.8501, 2.592	0.1818, not significant

**Table A64:** Odds ratio for the association of *Campylobacter* spp. and *Giardia duodenalis*.

	*Campylobacter* spp.	Non-*Campylobacter* spp.
*Giardia duodenalis*	164	231
Non-*Giardia duodenalis*	67	246
Odds ratio	95% confidence interval	Significance level P
2.607	1.862, 3.649	<0.0001, significant

**Table A65:** Odds ratio for the association of *Campylobacter* spp. and *Cyclospora* spp.

	*Campylobacter* spp.	Non-*Campylobacter* spp.
*Cyclospora* spp.	30	42
Non-*Cyclospora* spp.	201	435
Odds ratio	95% confidence interval	Significance level P
1.546	0.9399, 2.542	0.0865, not significant

**Table A66:** Odds ratio for the association of *Campylobacter* spp. and *Cryptosporidium* spp.

	*Campylobacter* spp.	Non-*Campylobacter* spp.
*Cryptosporidium* spp.	6	11
Non-*Cryptosporidium* spp.	225	466
Odds ratio	95% confidence interval	Significance level P
1.130	0.4124, 3.094	0.7978, not significant

**Table A67:** Odds ratio for the association of *Campylobacter* spp. and *Ascaris* spp.

	*Campylobacter* spp.	Non-*Campylobacter* spp.
*Ascaris* spp.	54	74
Non-*Ascaris* spp.	177	403
Odds ratio	95% confidence interval	Significance level P
1.661	1.122, 2.461	0.0125, significant

**Table A68:** Odds ratio for the association of *Campylobacter* spp. and *Necator americanus*.

	*Campylobacter* spp.	Non-*Campylobacter* spp.
*Necator americanus*	35	51
Non-*Necator americanus*	196	426
Odds ratio	95% confidence interval	Significance level P
1.492	0.9394, 2.368	0.1101, not significant

**Table A69:** Odds ratio for the association of *Campylobacter* spp. and *Strongyloides* spp.

	*Campylobacter* spp.	Non-*Campylobacter* spp.
*Strongyloides* spp.	14	25
Non-*Strongyloides* spp.	217	452
Odds ratio	95% confidence interval	Significance level P
1.116	0.5944, 2.289	0.7256, not significant

**Table A70:** Odds ratio for the association of *Campylobacter* spp. and *Taenia* spp.

	*Campylobacter* spp.	Non-*Campylobacter* spp.
*Taenia* spp.	18	15
Non-*Taenia* spp.	213	462
Odds ratio	95% confidence interval	Significance level P
2.603	1.287, 5.264	0.0078, significant

**Table A71:** Odds ratio for the association of *Campylobacter* spp. and *Hymenolepis* spp.

	*Campylobacter* spp.	Non-*Campylobacter* spp.
*Hymenolepis* spp.	52	64
Non-*Hymenolepis* spp.	179	413
Odds ratio	95% confidence interval	Significance level P
1.875	1.249, 2.813	0.0033, significant

**Table A72:** Odds ratio for the association of *Campylobacter* spp. and *Trichuris* spp.

	*Campylobacter* spp.	Non-*Campylobacter* spp.
*Trichuris* spp.	93	61
Non-*Trichuris* spp.	138	416
Odds ratio	95% confidence interval	Significance level P
4.596	3.155, 6.694	<0.0001, significant

**Table A73:** Odds ratio for the association of *Campylobacter* spp. and *Schistosoma* spp.

	*Campylobacter* spp.	Non-*Campylobacter* spp.
*Schistosoma* spp.	1	2
Non-*Schistosoma* spp.	212	450
Odds ratio	95% confidence interval	Significance level P
2.123	0.2968, 15.179	0.5973, not significant

**Table A74:** Odds ratio for the association of *Campylobacter* spp. and *Enterobius vermicularis*.

	*Campylobacter* spp.	Non-*Campylobacter* spp.
*Enterobius vermicularis*	9	19
Non-*Enterobius vermicularis*	222	458
Odds ratio	95% confidence interval	Significance level P
0.9772	0.4350, 2.195	1.0000, not significant

**Table A75:** Odds ratio for the association of *Campylobacter* spp. and *Tropheryma whipplei*.

	*Campylobacter* spp.	Non-*Campylobacter* spp.
*Tropheryma whipplei*	5	16
Non-*Tropheryma whipplei*	82	242
Odds ratio	95% confidence interval	Significance level P
0.9223	0.3275, 2.597	1.000, not significant

**Table A76:** Odds ratio for the association of *Campylobacter* spp. and enteropathogenic *Escherichia coli*.

	*Campylobacter* spp.	Non-*Campylobacter* spp.
Enteropathogenic *Escherichia coli*	54	134
Non-enteropathogenic *Escherichia coli*	127	206
Odds ratio	95% confidence interval	Significance level P
0.6537	0.4445, 0.9613	0.0350, significant

**Table A77:** Odds ratio for the association of *Campylobacter* spp. and enterotoxigenic *Escherichia coli*.

	*Campylobacter* spp.	Non-*Campylobacter* spp.
Enterotoxigenic *Escherichia coli*	82	47
Non-enterotoxigenic *Escherichia coli*	133	259
Odds ratio	95% confidence interval	Significance level P
3.398	2.243, 5.146	<0.0001, significant

**Table A78:** Odds ratio for the association of *Campylobacter* spp. and enteroaggregative *Escherichia coli*.

	*Campylobacter* spp.	Non-*Campylobacter* spp.
Enteroaggregative *Escherichia coli*	60	107
Non-enteroaggregative *Escherichia coli*	121	233
Odds ratio	95% confidence interval	Significance level P
1.080	0.7347, 1.587	0.6946, not significant

**Table A79:** Odds ratio for the association of *Campylobacter* spp. and Shiga toxin-producing *Escherichia coli*.

	*Campylobacter* spp.	Non-*Campylobacter* spp.
Shiga toxin-producing *Escherichia coli*	3	17
Non-Shiga toxin-producing *Escherichia coli*	38	70
Odds ratio	95% confidence interval	Significance level P
0.3251	0.08951, 1.181	0.1158, not significant

**Table A80:** Odds ratio for the association of *Campylobacter* spp. and *Dientamoeba fragilis*.

	*Campylobacter* spp.	Non-*Campylobacter* spp.
*Dientamoeba fragilis*	38	37
Non-*Dientamoeba fragilis*	193	440
Odds ratio	95% confidence interval	Significance level P
2.341	1.444, 3.797	0.0007, significant

**Table A81:** Odds ratio for the association of *Campylobacter* spp. and *Blastocystis hominis*.

	*Campylobacter* spp.	Non-*Campylobacter* spp.
*Blastocystis hominis*	144	225
Non-*Blastocystis hominis*	87	252
Odds ratio	95% confidence interval	Significance level P
1.854	1.344, 2.556	0.0002, significant

**Table A82:** Odds ratio for the association of *Campylobacter* spp. and *Endolimax nana*.

	*Campylobacter* spp.	Non-*Campylobacter* spp.
*Endolimax nana*	71	127
Non-*Endolimax nana*	141	326
Odds ratio	95% confidence interval	Significance level P
1.293	0.9096, 1.837	0.1722, not significant

**Table A83:** Odds ratio for the association of *Campylobacter* spp. and *Entamoeba coli*.

	*Campylobacter* spp.	Non-*Campylobacter* spp.
*Entamoeba coli*	60	144
Non-*Entamoeba coli*	152	308
Odds ratio	95% confidence interval	Significance level P
0.8443	0.5899, 1.208	0.3683, not significant

**Table A84:** Odds ratio for the association of *Campylobacter* spp. and *Entamoeba dispar*.

	*Campylobacter* spp.	Non-*Campylobacter* spp.
*Entamoeba dispar*	36	75
Non-*Entamoeba dispar*	176	378
Odds ratio	95% confidence interval	Significance level P
1.031	0.6667, 1.594	0.9113, not significant

**Table A85:** Odds ratio for the association of *Campylobacter* spp. and *Iodamoeba buetschlii*.

	*Campylobacter* spp.	Non-*Campylobacter* spp.
*Iodamoeba buetschlii*	10	36
Non-*Iodamoeba buetschlii*	202	417
Odds ratio	95% confidence interval	Significance level P
0.5734	0.2789, 1.179	0.1419, not significant

**Table A86:** Odds ratio for the association of *Campylobacter* spp. and *Pentatrichomonas hominis*.

	*Campylobacter* spp.	Non-*Campylobacter* spp.
*Pentatrichomonas hominis*	1	2
Non-*Pentatrichomonas hominis*	210	451
Odds ratio	95% confidence interval	Significance level P
1.074	0.09677, 11.916	1.0000, not significant

**Table A87:** Odds ratio for the association of *Campylobacter* spp. and *Encephalocytozoon* spp.

	*Campylobacter* spp.	Non-*Campylobacter* spp.
*Encephalocytozoon* spp.	1	6
Non-*Encephalocytozoon* spp.	131	149
Odds ratio	95% confidence interval	Significance level P
0.1896	0.02252, 1.596	0.1289, not significant

**Table A88:** Odds ratio for the association of *Campylobacter* spp. and *Aeromonas* spp.

	*Campylobacter* spp.	Non-*Campylobacter* spp.
*Aeromonas* spp.	3	2
Non-*Aeromonas* spp.	129	153
Odds ratio	95% confidence interval	Significance level P
1.779	0.2926, 10.816	0.6641, not significant

**Table A89:** Odds ratio for the association of *Campylobacter* spp. and conidia.

	*Campylobacter* spp.	Non-*Campylobacter* spp.
Conidia	21	20
Non-conidia	93	111
Odds ratio	95% confidence interval	Significance level P
0.6072	0.6402, 2.453	0.6072

**Table A90:** Odds ratio for the association of *Campylobacter* spp. and pseudoconidia.

	*Campylobacter* spp.	Non-*Campylobacter* spp.
Pseudoconidia	13	14
Non-pseudoconidia	101	117
Odds ratio	95% confidence interval	Significance level P
1.076	0.4830, 2.396	1.0000, not significant

**Table A91:** Odds ratio for the association of *Yersinia* spp. and *Entamoeba histolytica*.

	*Yersinia* spp.	Non-*Yersinia* spp.
*Entamoeba histolytica*	0	56
Non-*Entamoeba histolytica*	4	647
Odds ratio	95% confidence interval	Significance level P
1.273	0.06765, 23.969	1.0000, not significant

**Table A92:** Odds ratio for the association of *Yersinia* spp. and *Giardia duodenalis*.

	*Yersinia* spp.	Non-*Yersinia* spp.
*Giardia duodenalis*	1	395
Non-*Giardia duodenalis*	3	309
Odds ratio	95% confidence interval	Significance level P
0.2608	0.02698, 2.521	0.3258, not significant

**Table A93:** Odds ratio for the association of *Yersinia* spp. and *Cyclospora* spp.

	*Yersinia* spp.	Non-*Yersinia* spp.
*Cyclospora* spp.	0	72
Non-*Cyclospora* spp.	4	632
Odds ratio	95% confidence interval	Significance level P
0.9693	0.05163, 18.200	1.0000, not significant

**Table A94:** Odds ratio for the association of *Yersinia* spp. and *Cryptosporidium* spp.

	*Yersinia* spp.	Non-*Yersinia* spp.
*Cryptosporidium* spp.	0	17
Non-*Cryptosporidium* spp.	4	687
Odds ratio	95% confidence interval	Significance level P
4.365	0.2260, 84.300	1.0000, not significant

**Table A95:** Odds ratio for the association of *Yersinia* spp. and *Ascaris* spp.

	*Yersinia* spp.	Non-*Yersinia* spp.
*Ascaris* spp.	0	128
Non-*Ascaris* spp.	4	576
Odds ratio	95% confidence interval	Significance level P
0.4985	0.02665, 9.323	1.0000, not significant

**Table A96:** Odds ratio for the association of *Yersinia* spp. and *Necator americanus*.

	*Yersinia* spp.	Non-*Yersinia* spp.
*Necator americanus*	0	86
Non-*Necator americanus*	4	618
Odds ratio	95% confidence interval	Significance level P
0.7945	0.04238, 14.895	1.0000, not significant

**Table A97:** Odds ratio for the association of *Yersinia* spp. and *Strongyloides* spp.

	*Yersinia* spp.	Non-*Yersinia* spp.
*Strongyloides* spp.	0	39
Non-*Strongyloides* spp.	4	665
Odds ratio	95% confidence interval	Significance level P
1.872	0.09896, 35.412	1.0000, not significant

**Table A98:** Odds ratio for the association of *Yersinia* spp. and *Taenia* spp.

	*Yersinia* spp.	Non-*Yersinia* spp.
*Taenia* spp.	0	33
Non-*Taenia* spp.	4	671
Odds ratio	95% confidence interval	Significance level P
2.227	0.1174, 42.256	1.000, not significant

**Table A99:** Odds ratio for the association of *Yersinia* spp. and *Hymenolepis* spp.

	*Yersinia* spp.	Non-*Yersinia* spp.
*Hymenolepis* spp.	0	116
Non-*Hymenolepis* spp.	4	588
Odds ratio	95% confidence interval	Significance level P
0.5613	0.02999, 10.503	1.0000, not significant

**Table A100:** Odds ratio for the association of *Yersinia* spp. and *Trichuris* spp.

	*Yersinia* spp.	Non-*Yersinia* spp.
*Trichuris* spp.	0	154
Non-*Trichuris* spp.	4	550
Odds ratio	95% confidence interval	Significance level P
0.3959	0.02118, 7.399	0.5819, not significant

**Table A101:** Odds ratio for the association of *Yersinia* spp. and *Schistosoma* spp.

	*Yersinia* spp.	Non-*Yersinia* spp.
*Schistosoma* spp.	0	3
Non-*Schistosoma* spp.	4	659
Odds ratio	95% confidence interval	Significance level P
20.937	0.9379, 467.36	1.0000, not significant

**Table A102:** Odds ratio for the association of *Yersinia* spp. and *Enterobius vermicularis*.

	*Yersinia* spp.	Non-*Yersinia* spp.
*Enterobius vermicularis*	0	28
Non-*Enterobius vermicularis*	4	676
Odds ratio	95% confidence interval	Significance level P
2.637	0.1385, 50.210	1.0000, not significant

**Table A103:** Odds ratio for the association of *Yersinia* spp. and *Dientamoeba fragilis*.

	*Yersinia* spp.	Non-*Yersinia* spp.
*Dientamoeba fragilis*	0	75
Non-*Dientamoeba fragilis*	4	629
Odds ratio	95% confidence interval	Significance level P
0.9264	0.4936, 17.388	1.0000, not significant

**Table A104:** Odds ratio for the association of *Yersinia* spp. and *Blastocystis* spp.

	*Yersinia* spp.	Non-*Yersinia* spp.
*Blastocystis* spp.	0	369
Non-*Blastocystis* spp.	4	335
Odds ratio	95% confidence interval	Significance level P
0.1009	0.005408, 1.882	0.0521, not significant

**Table A105:** Odds ratio for the association of *Yersinia* spp. and *Endolimax nana*.

	*Yersinia* spp.	Non-*Yersinia* spp.
*Endolimax nana*	1	197
Non-*Endolimax nana*	2	465
Odds ratio	95% confidence interval	Significance level P
1.180	0.1063, 13.099	1.0000, not significant

**Table A106:** Odds ratio for the association of *Yersinia* spp. and *Entamoeba coli*.

	*Yersinia* spp.	Non-*Yersinia* spp.
*Entamoeba coli*	0	204
Non-*Entamoeba coli*	4	456
Odds ratio	95% confidence interval	Significance level P
0.2480	0.01328, 4.632	0.3180, not significant

**Table A107:** Odds ratio for the association of *Yersinia* spp. and *Entamoeba dispar*.

	*Yersinia* spp.	Non-*Yersinia* spp.
*Entamoeba dispar*	0	111
Non-*Entamoeba dispar*	4	550
Odds ratio	95% confidence interval	Significance level P
0.5486	0.02931, 10.269	1.0000, not significant

**Table A108:** Odds ratio for the association of *Yersinia* spp. and *Iodamoeba buetschlii*.

	*Yersinia* spp.	Non-*Yersinia* spp.
*Iodamoeba buetschlii*	0	46
Non-*Iodamoeba buetschlii*	4	615
Odds ratio	95% confidence interval	Significance level P
1.471	0.07794, 27.754	1.0000, not significant

**Table A109:** Odds ratio for the association of *Yersinia* spp. and *Pentatrichomonas hominis*.

	*Salmonella* spp.	Non-*Salmonella* spp.
*Pentatrichomonas hominis*	0	3
Non-*Pentatrichomonas hominis*	4	657
Odds ratio	95% confidence interval	Significance level P
20.873	0.9351, 465.94	1.0000, not significant

**Table A110:** Odds ratio for the association of *Entamoeba histolytica* and *Giardia duodenalis*.

	*Entamoeba histolytica*	Non-*Entamoeba histolytica*
*Giardia duodenalis*	35	360
Non-*Giardia duodenalis*	21	291
Odds ratio	95% confidence interval	Significance level P
1.347	0.7674, 2.365	0.3283, not significant

**Table A111:** Odds ratio for the association of *Entamoeba histolytica* and *Cyclospora* spp.

	*Entamoeba histolytica*	Non-*Entamoeba histolytica*
*Cyclospora* spp.	10	62
Non-*Cyclospora* spp.	46	589
Odds ratio	95% confidence interval	Significance level P
2.065	0.9929, 4.296	0.0623, not significant

**Table A112:** Odds ratio for the association of *Entamoeba histolytica* and *Cryptosporidium* spp.

	*Entamoeba histolytica*	Non-*Entamoeba histolytica*
*Cryptosporidium* spp.	4	13
Non-*Cryptosporidium* spp.	52	683
Odds ratio	95% confidence interval	Significance level P
3.775	1.188, 11.995	0.0386, significant

**Table A113:** Odds ratio for the association of *Entamoeba histolytica* and *Ascaris* spp.

	*Entamoeba histolytica*	Non-*Entamoeba histolytica*
*Ascaris* spp.	18	110
Non-*Ascaris* spp.	38	541
Odds ratio	95% confidence interval	Significance level P
2.330	1.282, 4.233	0.0101, significant

**Table A114:** Odds ratio for the association of *Entamoeba histolytica* and *Necator americanus*.

	*Entamoeba histolytica*	Non-*Entamoeba histolytica*
*Necator americanus*	7	79
Non-*Necator americanus*	49	572
Odds ratio	95% confidence interval	Significance level P
1.034	0.4527, 2.363	0.8342, not significant

**Table A115:** Odds ratio for the association of *Entamoeba histolytica* and *Strongyloides* spp.

	*Entamoeba histolytica*	Non-*Entamoeba histolytica*
*Strongyloides* spp.	11	28
Non-*Strongyloides* spp.	45	623
Odds ratio	95% confidence interval	Significance level P
5.439	2.543, 11.635	<0.0001, significant

**Table A116:** Odds ratio for the association of *Entamoeba histolytica* and *Taenia* spp.

	*Entamoeba histolytica*	Non-*Entamoeba histolytica*
*Taenia* spp.	4	29
Non-*Taenia* spp.	52	622
Odds ratio	95% confidence interval	Significance level P
1.650	0.5585, 4.874	0.3222, not significant

**Table A117:** Odds ratio for the association of *Entamoeba histolytica* and *Hymenolepis* spp.

	*Entamoeba histolytica*	Non-*Entamoeba histolytica*
*Hymenolepis* spp.	35	360
Non-*Hymenolepis* spp.	21	291
Odds ratio	95% confidence interval	Significance level P
1.347	0.7674, 2.365	0.3283, not significant

**Table A118:** Odds ratio for the association of *Entamoeba histolytica* and *Trichuris* spp.

	*Entamoeba histolytica*	Non-*Entamoeba histolytica*
*Trichuris* spp.	23	131
Non-*Trichuris* spp.	33	520
Odds ratio	95% confidence interval	Significance level P
2.767	1.571, 4.872	0.0006, significant

**Table A119:** Odds ratio for the association of *Entamoeba histolytica* and *Enterobius vermicularis*.

	*Entamoeba histolytica*	Non-*Entamoeba histolytica*
*Enterobius vermicularis*	3	25
Non-*Enterobius vermicularis*	53	626
Odds ratio	95% confidence interval	Significance level P
1.417	0.4142, 4.850	0.4798, not significant

**Table A120:** Odds ratio for the association of *Entamoeba histolytica* and *Schistosoma* spp.

	*Entamoeba histolytica*	Non-*Entamoeba histolytica*
*Schistosoma* spp.	1	2
Non-*Schistosoma* spp.	55	607
Odds ratio	95% confidence interval	Significance level P
5.518	0.4922, 61.863	0.2323, not significant

**Table A121:** Odds ratio for the association of *Entamoeba histolytica* and *Tropheryma whipplei*.

	*Entamoeba histolytica*	Non-*Entamoeba histolytica*
*Tropheryma whipplei*	0	21
Non-*Tropheryma whipplei*	14	310
Odds ratio	95% confidence interval	Significance level P
0.4980	0.02870, 8.640	1.0000, not significant

**Table A122:** Odds ratio for the association of *Entamoeba histolytica* and enteropathogenic *Escherichia coli*.

	*Entamoeba histolytica*	Non-*Entamoeba histolytica*
Enteropathogenic *Escherichia coli*	18	170
Non-enteropathogenic *Escherichia coli*	34	299
Odds ratio	95% confidence interval	Significance level P
0.9311	0.5102, 1.700	0.8798, not significant

**Table A123:** Odds ratio for the association of *Entamoeba histolytica* and enterotoxigenic *Escherichia coli*.

	*Entamoeba histolytica*	Non-*Entamoeba histolytica*
Enterotoxigenic *Escherichia coli*	12	117
Non-enterotoxigenic *Escherichia coli*	40	352
Odds ratio	95% confidence interval	Significance level P
0.9026	0.4580, 1.779	0.8664, not significant

**Table A124:** Odds ratio for the association of *Entamoeba histolytica* and enteroaggregative *Escherichia coli*.

	*Entamoeba histolytica*	Non-*Entamoeba histolytica*
Enteroaggregative *Escherichia coli*	18	149
Non-enteroaggregative *Escherichia coli*	33	321
Odds ratio	95% confidence interval	Significance level P
1.175	0.6408, 2.155	0.6365, not significant

**Table A125:** Odds ratio for the association of *Entamoeba histolytica* and Shiga toxin-producing *Escherichia coli*.

	*Entamoeba histolytica*	Non-*Entamoeba histolytica*
Shiga toxin-producing *Escherichia coli*	1	19
Non-Shiga toxin-producing *Escherichia coli*	8	100
Odds ratio	95% confidence interval	Significance level P
0.6579	0.07768, 5.572	1.0000, not significant

**Table A126:** Odds ratio for the association of *Entamoeba histolytica* and *Dientamoeba fragilis*.

	*Entamoeba histolytica*	Non-*Entamoeba histolytica*
*Dientamoeba fragilis*	15	60
Non-*Dientamoeba fragilis*	41	591
Odds ratio	95% confidence interval	Significance level P
3.604	1.884, 6.892	0.0003, significant

**Table A127:** Odds ratio for the association of *Entamoeba histolytica* and *Blastocystis* spp.

	*Entamoeba histolytica*	Non-*Entamoeba histolytica*
*Blastocystis* spp.	34	355
Non-*Blastocystis* spp.	22	296
Odds ratio	95% confidence interval	Significance level P
1.289	0.7374, 2.252	0.4034, not significant

**Table A128:** Odds ratio for the association of *Entamoeba histolytica* and *Endolimax nana*.

	*Entamoeba histolytica*	Non-*Entamoeba histolytica*
*Endolimax nana*	20	178
Non-*Endolimax nana*	35	431
Odds ratio	95% confidence interval	Significance level P
1.384	0.7773, 2.463	0.2828, not significant

**Table A129:** Odds ratio for the association of *Entamoeba histolytica* and *Entamoeba coli*.

	*Entamoeba histolytica*	Non-*Entamoeba histolytica*
*Entamoeba coli*	21	183
Non-*Entamoeba coli*	34	425
Odds ratio	95% confidence interval	Significance level P
1.434	0.8104, 2.539	0.2241, not significant

**Table A130:** Odds ratio for the association of *Entamoeba histolytica* and *Entamoeba dispar*.

	*Entamoeba histolytica*	Non-*Entamoeba histolytica*
*Entamoeba dispar*	14	97
Non-*Entamoeba dispar*	41	512
Odds ratio	95% confidence interval	Significance level P
1.802	0.9461, 3.434	0.0874, not significant

**Table A131:** Odds ratio for the association of *Entamoeba histolytica* and *Iodamoeba buetschlii*.

	*Entamoeba histolytica*	Non-*Entamoeba histolytica*
*Iodamoeba buetschlii*	2	43
Non-*Iodamoeba buetschlii*	53	566
Odds ratio	95% confidence interval	Significance level P
0.4967	0.1170, 2.109	0.5715, not significant

**Table A132:** Odds ratio for the association of *Entamoeba histolytica* and *Encephalocytozoon* spp.

	*Entamoeba histolytica*	Non-*Entamoeba histolytica*
*Encephalocytozoon* spp.	0	7
Non-*Encephalocytozoon* spp.	37	243
Odds ratio	95% confidence interval	Significance level P
0.4329	0.02420, 7.742	0.6005, not significant

**Table A133:** Odds ratio for the association of *Entamoeba histolytica* and *Aeromonas* spp.

	*Entamoeba histolytica*	Non-*Entamoeba histolytica*
*Aeromonas* spp.	0	5
Non-*Aeromonas* spp.	37	245
Odds ratio	95% confidence interval	Significance level P
0.5952	0.03222, 10.992	1.0000, not significant

**Table A134:** Odds ratio for the association of *Entamoeba histolytica* and conidia.

	*Entamoeba histolytica*	Non-*Entamoeba histolytica*
conidia	8	33
Non-conidia	29	175
Odds ratio	95% confidence interval	Significance level P
1.463	0.6148, 3.481	0.4721, not significant

**Table A135:** *Entamoeba histolytica* and pseudoconidia.

	*Entamoeba histolytica*	Non-*Entamoeba histolytica*
Pseudoconidia	5	22
Non-pseudoconidia	32	186
Odds ratio	95% confidence interval	Significance level P
1.321	0.4664, 3.742	0.5733, not significant

**Table A136:** Odds ratio for the association of *Giardia duodenalis* and *Cyclospora* spp.

	*Giardia duodenalis*	Non-*Giardia duodenalis*
*Cyclospora* spp.	48	24
Non-*Cyclospora* spp.	354	347
Odds ratio	95% confidence interval	Significance level P
1.960	1.175, 3.271	0.0093, significant

**Table A137:** Odds ratio for the association of *Giardia duodenalis* and *Cryptosporidium* spp.

	*Giardia duodenalis*	Non-*Giardia duodenalis*
*Cryptosporidium* spp.	11	6
Non-*Cryptosporidium* spp.	391	365
Odds ratio	95% confidence interval	Significance level P
1.711	0.6264, 4.676	0.3337, not significant

**Table A138:** Odds ratio for the association of *Giardia duodenalis* and *Ascaris* spp.

	*Giardia duodenalis*	Non-*Giardia duodenalis*
*Ascaris* spp.	75	55
Non-*Ascaris* spp.	327	316
Odds ratio	95% confidence interval	Significance level P
1.318	0.9004, 1.929	0.1778, not significant

**Table A139:** Odds ratio for the association of *Giardia duodenalis* and *Necator americanus*.

	*Giardia duodenalis*	Non-*Giardia duodenalis*
*Necator americanus*	48	38
Non-*Necator americanus*	354	333
Odds ratio	95% confidence interval	Significance level P
1.188	0.7567, 1.866	0.4930, not significant

**Table A140:** Odds ratio for the association of *Giardia duodenalis* and *Strongyloides* spp.

	*Giardia duodenalis*	Non-*Giardia duodenalis*
*Strongyloides* spp.	19	20
Non-*Strongyloides* spp.	378	356
Odds ratio	95% confidence interval	Significance level P
0.8947	0.4696, 1.704	0.7455, not significant

**Table A141:** Odds ratio for the association of *Giardia duodenalis* and *Taenia* spp.

	*Giardia duodenalis*	Non-*Giardia duodenalis*
*Taenia* spp.	20	13
Non-*Taenia* spp.	382	358
Odds ratio	95% confidence interval	Significance level P
1.442	0.7066, 2.942	0.3745, not significant

**Table A142:** Odds ratio for the association of *Giardia duodenalis* and *Hymenolepis* spp.

	*Giardia duodenalis*	Non-*Giardia duodenalis*
*Hymenolepis* spp.	83	43
Non-*Hymenolepis* spp.	319	328
Odds ratio	95% confidence interval	Significance level P
1.985	1.331, 2.959	0.0006, significant

**Table A143:** Odds ratio for the association of *Giardia duodenalis* and *Trichuris* spp.

	*Giardia duodenalis*	Non-*Giardia duodenalis*
*Trichuris* spp.	102	56
Non-*Trichuris* spp.	300	315
Odds ratio	95% confidence interval	Significance level P
1.913	1.331, 2.748	0.0005, significant

**Table A144:** Odds ratio for the association of *Giardia duodenalis* and *Schistosoma* spp.

	*Giardia duodenalis*	Non-*Giardia duodenalis*
*Schistosoma* spp.	2	2
Non-*Schistosoma* spp.	384	343
Odds ratio	95% confidence interval	Significance level P
0.8932	0.1251, 6.379	1.0000, not significant

**Table A145:** Odds ratio for the association of *Giardia duodenalis* and *Enterobius vermicularis*.

	*Giardia duodenalis*	Non-*Giardia duodenalis*
*Enterobius vermicularis*	15	16
Non-*Enterobius vermicularis*	387	355
Odds ratio	95% confidence interval	Significance level P
0.8600	0.4189, 1.765	0.7166, not significant

**Table A146:** Odds ratio for the association of *Giardia duodenalis* and *Tropheryma whipplei*.

	*Giardia duodenalis*	Non-*Giardia duodenalis*
*Tropheryma whipplei*	11	17
Non-*Tropheryma whipplei*	190	171
Odds ratio	95% confidence interval	Significance level P
0.5824	0.2653, 1.278	0.2385, not significant

**Table A147:** Odds ratio for the association of *Giardia duodenalis* and enteropathogenic *Escherichia coli*.

	*Giardia duodenalis*	Non-*Giardia duodenalis*
Enteropathogenic *Escherichia coli*	107	103
Non-enteropathogenic *Escherichia coli*	220	135
Odds ratio	95% confidence interval	Significance level P
0.6375	0.4514, 0.9003	0.0108, significant

**Table A148:** Odds ratio for the association of *Giardia duodenalis* and enterotoxigenic *Escherichia coli*.

	*Giardia duodenalis*	Non-*Giardia duodenalis*
Enterotoxigenic *Escherichia coli*	87	50
Non-enterotoxigenic *Escherichia coli*	240	188
Odds ratio	95% confidence interval	Significance level P
1.363	0.9165, 2.027	0.1365, not significant

**Table A149:** Odds ratio for the association of *Giardia duodenalis* and enteroaggregative *Escherichia coli*.

	*Giardia duodenalis*	Non-*Giardia duodenalis*
Enteroaggregative *Escherichia coli*	92	85
Non-enteroaggregative *Escherichia coli*	235	153
Odds ratio	95% confidence interval	Significance level P
0.7047	0.4924, 1.008	0.0661, not significant

**Table A150:** Odds ratio for the association of *Giardia duodenalis* and Shiga toxin-producing *Escherichia coli*.

	*Giardia duodenalis*	Non-*Giardia duodenalis*
Shiga-toxin-producing *Escherichia coli*	10	10
Non-Shiga toxin-producing *Escherichia coli*	54	54
Odds ratio	95% confidence interval	Significance level P
1.0000	0.3850, 2.597	1.1917, not significant

**Table A151:** Odds ratio for the association of *Giardia duodenalis* and *Dientamoeba fragilis*.

	*Giardia duodenalis*	Non-*Giardia duodenalis*
*Dientamoeba fragilis*	49	26
Non-*Dientamoeba fragilis*	353	345
Odds ratio	95% confidence interval	Significance level P
1.842	1.119, 3.032	0.0153, significant

**Table A152:** Odds ratio for the association of *Giardia duodenalis* and *Blastocystis* spp.

	*Giardia duodenalis*	Non-*Giardia duodenalis*
*Blastocystis* spp.	234	157
Non-*Blastocystis* spp.	168	214
Odds ratio	95% confidence interval	Significance level P
1.899	1.426, 2.527	<0.0001, significant

**Table A153:** Odds ratio for the association of *Giardia duodenalis* and *Endolimax nana*.

	*Giardia duodenalis*	Non-*Giardia duodenalis*
*Endolimax nana*	125	91
Non-*Endolimax nana*	260	254
Odds ratio	95% confidence interval	Significance level P
1.342	0.9737, 1.849	0.0745, not significant

**Table A154:** Odds ratio for the association of *Giardia duodenalis* and *Entamoeba coli*.

	*Giardia duodenalis*	Non-*Giardia duodenalis*
*Entamoeba coli*	128	99
Non-*Entamoeba coli*	256	247
Odds ratio	95% confidence interval	Significance level P
1.242	0.9063, 1.703	0.2000, not significant

**Table A155:** Odds ratio for the association of *Giardia duodenalis* and *Entamoeba dispar*.

	*Giardia duodenalis*	Non-*Giardia duodenalis*
*Entamoeba dispar*	72	46
Non-*Entamoeba dispar*	313	299
Odds ratio	95% confidence interval	Significance level P
1.495	0.9997, 2.236	0.0557, not significant

**Table A156:** Odds ratio for the association of *Giardia duodenalis* and *Iodamoeba buetschlii*.

	*Giardia duodenalis*	Non-*Giardia duodenalis*
*Iodamoeba buetschlii*	29	26
Non-*Iodamoeba buetschlii*	350	325
Odds ratio	95% confidence interval	Significance level P
1.036	0.5972, 1.796	1.0000, not significant

**Table A157:** Odds ratio for the association of *Giardia duodenalis* and *Pentatrichomonas hominis*.

	*Giardia duodenalis*	Non-*Giardia duodenalis*
*Pentatrichomonas hominis*	3	0
Non-*Pentatrichomonas hominis*	381	345
Odds ratio	95% confidence interval	Significance level P
6.339	0.3261, 123.26	0.2511, not significant

**Table A158:** Odds ratio for the association of *Giardia duodenalis* and *Encephalocytozoon* spp.

	*Giardia duodenalis*	Non-*Giardia duodenalis*
*Encephalocytozoon* spp.	5	2
Non-*Encephalocytozoon* spp.	176	104
Odds ratio	95% confidence interval	Significance level P
1.477	0.2814, 7.754	1.0000, not significant

**Table A159:** Odds ratio for the association of *Giardia duodenalis* and *Aeromonas* spp.

	*Giardia duodenalis*	Non-*Giardia duodenalis*
*Aeromonas* spp.	4	1
Non-*Aeromonas* spp.	107	175
Odds ratio	95% confidence interval	Significance level P
6.542	0.7213, 59.339	0.0755, not significant

**Table A160:** Odds ratio for the association of *Giardia duodenalis* and conidia.

	*Giardia duodenalis*	Non-*Giardia duodenalis*
Conidia	30	11
Non-conidia	135	69
Odds ratio	95% confidence interval	Significance level P
1.394	0.6588, 2.949	0.4668, not significant

**Table A161:** Odds ratio for the association of *Giardia duodenalis* and pseudoconidia.

	*Giardia duodenalis*	Non-*Giardia duodenalis*
Pseudoconidia	18	9
Non-pseudoconidia	147	71
Odds ratio	95% confidence interval	Significance level P
0.9660	0.4133, 2.258	1.0000, not significant

**Table A162:** Odds ratio for the association of *Cyclospora* spp. and *Cryptosporidium* spp.

	*Cyclospora* spp.	Non-*Cyclospora* spp.
*Cryptosporidium* spp.	1	16
Non-*Cryptosporidium* spp.	71	685
Odds ratio	95% confidence interval	Significance level P
0.6030	0.07876, 4.617	1.0000, not significant

**Table A163:** Odds ratio for the association of *Cyclospora* spp. and *Ascaris* spp.

	*Cyclospora* spp.	Non-*Cyclospora* spp.
*Ascaris* spp.	20	110
Non-*Ascaris* spp.	52	591
Odds ratio	95% confidence interval	Significance level P
2.066	1.187, 3.598	0.0128, significant

**Table A164:** Odds ratio for the association of *Cyclospora* spp. and *Necator americanus*.

	*Cyclospora* spp.	Non-*Cyclospora* spp.
*Necator americanus*	12	74
Non-*Necator americanus*	60	627
Odds ratio	95% confidence interval	Significance level P
1.695	0.8714, 3.296	0.1177, not significant

**Table A165:** Odds ratio for the association of *Cyclospora* spp. and *Strongyloides* spp.

	*Cyclospora* spp.	Non-*Cyclospora* spp.
*Strongyloides* spp.	4	35
Non-*Strongyloides* spp.	68	666
Odds ratio	95% confidence interval	Significance level P
1.119	0.3861, 3.245	0.7773, not significant

**Table A166:** Odds ratio for the association of *Cyclospora* spp. and *Taenia* spp.

	*Cyclospora* spp.	Non-*Cyclospora* spp.
*Taenia* spp.	5	28
Non-*Taenia* spp.	67	673
Odds ratio	95% confidence interval	Significance level P
1.794	0.6703, 4.800	0.2221, not significant

**Table A167:** Odds ratio for the association of *Cyclospora* spp. and *Hymenolepis* spp.

	*Cyclospora* spp.	Non-*Cyclospora* spp.
*Hymenolepis* spp.	15	111
Non-*Hymenolepis* spp.	57	590
Odds ratio	95% confidence interval	Significance level P
1.399	0.7646, 2.559	0.3133, not significant

**Table A168:** Odds ratio for the association of *Cyclospora* spp. and *Trichuris* spp.

	*Cyclospora* spp.	Non-*Cyclospora* spp.
*Trichuris* spp.	25	133
Non-*Trichuris* spp.	47	568
Odds ratio	95% confidence interval	Significance level P
2.272	1.350, 3.824	0.0032, significant

**Table A169:** Odds ratio for the association of *c*.

	*Cyclospora* spp.	Non-*Cyclospora* spp.
*Schistosoma* spp.	0	4
Non-*Schistosoma* spp.	72	655
Odds ratio	95% confidence interval	Significance level P
1.005	0.05351, 18.861	1.0000, not significant

**Table A170:** Odds ratio for the association of *Cyclospora* spp. and *Enterobius vermicularis*.

	*Cyclospora* spp.	Non-*Cyclospora* spp.
*Enterobius vermicularis*	5	26
Non-*Enterobius vermicularis*	67	675
Odds ratio	95% confidence interval	Significance level P
1.937	0.7201, 5.213	0.1983, not significant

**Table A171:** Odds ratio for the association of *Cyclospora* spp. and *Tropheryma whipplei*.

	*Cyclospora* spp.	Non-*Cyclospora* spp.
*Tropheryma whipplei*	0	28
Non-*Tropheryma whipplei*	29	332
Odds ratio	95% confidence interval	Significance level P
0.1977	0.01176, 3.324	0.2502, not significant

**Table A172:** Odds ratio for the association of *Cyclospora* spp. and enteropathogenic *Escherichia coli*.

	*Cyclospora* spp.	Non-*Cyclospora* spp.
Enteropathogenic *Escherichia coli*	24	185
Non-enteropathogenic *Escherichia coli*	46	310
Odds ratio	95% confidence interval	Significance level P
0.8743	0.5166, 1.480	0.6921, not significant

**Table A173:** Odds ratio for the association of *Cyclospora* spp. and enterotoxigenic *Escherichia coli*.

	*Cyclospora* spp.	Non-*Cyclospora* spp.
Enterotoxigenic *Escherichia coli*	24	113
Non-enterotoxigenic *Escherichia coli*	46	382
Odds ratio	95% confidence interval	Significance level P
1.764	1.031, 3.016	0.0516, not significant

**Table A174:** Odds ratio for the association of *Cyclospora* spp. and enteroaggregative *Escherichia coli*.

	*Cyclospora* spp.	Non-*Cyclospora* spp.
Enteroaggregative *Escherichia coli*	20	157
Non-enteroaggregative *Escherichia coli*	50	338
Odds ratio	95% confidence interval	Significance level P
0.8611	0.4958, 1.496	0.6802, not significant

**Table A175:** Odds ratio for the association of *Cyclospora* spp. and Shiga toxin-producing *Escherichia coli*.

	*Cyclospora* spp.	Non-*Cyclospora* spp.
Shiga toxin-producing *Escherichia coli*	1	19
Non-Shiga toxin-producing *Escherichia coli*	15	93
Odds ratio	95% confidence interval	Significance level P
0.3263	0.04060, 2.623	0.4641, not significant

**Table A176:** Odds ratio for the association of *Cyclospora* spp. and *Dientamoeba fragilis*.

	*Cyclospora* spp.	Non-*Cyclospora* spp.
*Dientamoeba fragilis*	14	61
Non-*Dientamoeba fragilis*	58	640
Odds ratio	95% confidence interval	Significance level P
2.533	1.335, 4.804	0.0101, significant

**Table A177:** Odds ratio for the association of *Cyclospora* spp. and *Blastocystis* spp.

	*Cyclospora* spp.	Non-*Cyclospora* spp.
*Blastocystis* spp.	39	352
Non-*Blastocystis* spp.	33	349
Odds ratio	95% confidence interval	Significance level P
1.172	0.7202, 1.906	0.5385, not significant

**Table A178:** Odds ratio for the association of *Cyclospora* spp. and *Endolimax nana*.

	*Cyclospora* spp.	Non-*Cyclospora* spp.
*Endolimax nana*	33	183
Non-*Endolimax nana*	39	475
Odds ratio	95% confidence interval	Significance level P
2.196	1.340, 3.600	0.0025, significant

**Table A179:** Odds ratio for the association of *Cyclospora* spp. and *Entamoeba coli*.

	*Cyclospora* spp.	Non-*Cyclospora* spp.
*Entamoeba coli*	33	194
Non-*Entamoeba coli*	39	463
Odds ratio	95% confidence interval	Significance level P
2.0189	1.233, 3.307	0.0069, significant

**Table A180:** Odds ratio for the association of *Cyclospora* spp. and *Entamoeba dispar*.

	*Cyclospora* spp.	Non-*Cyclospora* spp.
*Entamoeba dispar*	15	103
Non-*Entamoeba dispar*	57	555
Odds ratio	95% confidence interval	Significance level P
1.418	0.7731, 2.601	0.2425, not significant

**Table A181:** Odds ratio for the association of *Cyclospora* spp. and *Iodamoeba buetschlii*.

	*Cyclospora* spp.	Non-*Cyclospora* spp.
*Iodamoeba buetschlii*	7	48
Non-*Iodamoeba buetschlii*	65	610
Odds ratio	95% confidence interval	Significance level P
1.369	0.5947, 3.149	0.4778, not significant

**Table A182:** Odds ratio for the association of *Cyclospora* spp. and *Pentatrichomonas hominis*.

	*Cyclospora* spp.	Non-*Cyclospora* spp.
*Pentatrichomonas hominis*	1	2
Non-*Pentatrichomonas hominis*	71	655
Odds ratio	95% confidence interval	Significance level P
4.613	0.4128, 51.538	0.2683, not significant

**Table A183:** Odds ratio for the association of *Cyclospora* spp. and *Encephalocytozoon* spp.

	*Cyclospora* spp.	Non-*Cyclospora* spp.
*Encephalocytozoon* spp.	0	7
Non-*Encephalocytozoon* spp.	41	239
Odds ratio	95% confidence interval	Significance level P
0.3847	0.02155, 6.870	0.5985, not significant

**Table A184:** Odds ratio for the association of *Cyclospora* spp. and *Aeromonas* spp.

	*Cyclospora* spp.	Non-*Cyclospora* spp.
*Aeromonas* spp.	3	2
Non-*Aeromonas* spp.	38	244
Odds ratio	95% confidence interval	Significance level P
9.632	1.557, 59.564	0.0221, significant

**Table A185:** Odds ratio for the association of *Cyclospora* spp. and conidia.

	*Cyclospora* spp.	Non-*Cyclospora* spp.
Conidia	6	35
Non-conidia	35	169
Odds ratio	95% confidence interval	Significance level P
0.8278	0.3235, 2.118	0.8209, not significant

**Table A186:** Odds ratio for the association of *Cyclospora* spp. and pseudoconidia.

	*Cyclospora* spp.	Non-*Cyclospora* spp.
Pseudoconidia	6	21
Non-pseudoconidia	35	183
Odds ratio	95% confidence interval	Significance level P
1.494	0.5624, 3.968	0.4161, not significant

**Table A187:** Odds ratio for the association of *Cryptosporidium* spp. and *Ascaris* spp.

	*Cryptosporidium* spp.	Non-*Cryptosporidium* spp.
*Ascaris* spp.	4	126
Non-*Ascaris* spp.	13	630
Odds ratio	95% confidence interval	Significance level P
1.538	0.4934, 4.797	0.5074, not significant

**Table A188:** Odds ratio for the association of *Cryptosporidium* spp. and *Necator americanus*.

	*Cryptosporidium* spp.	Non-*Cryptosporidium* spp.
*Necator americanus*	1	85
Non-*Necator americanus*	16	671
Odds ratio	95% confidence interval	Significance level P
0.4934	0.06458, 3.769	0.7097, not significant

**Table A189:** Odds ratio for the association of *Cryptosporidium* spp. and *Strongyloides* spp.

	*Cryptosporidium* spp.	Non-*Cryptosporidium* spp.
*Strongyloides* spp.	2	37
Non-*Strongyloides* spp.	15	719
Odds ratio	95% confidence interval	Significance level P
2.591	0.5710, 11.756	0.2098, not significant

**Table A190:** Odds ratio for the association of *Cryptosporidium* spp. and *Taenia* spp.

	*Cryptosporidium* spp.	Non-*Cryptosporidium* spp.
*Taenia* spp.	1	32
Non-*Taenia* spp.	16	724
Odds ratio	95% confidence interval	Significance level P
1.414	0.1818, 11.002	0.5275, not significant

**Table A191:** Odds ratio for the association of *Cryptosporidium* spp. and *Hymenolepis* spp.

	*Cryptosporidium* spp.	Non-*Cryptosporidium* spp.
*Hymenolepis* spp.	3	123
Non-*Hymenolepis* spp.	14	633
Odds ratio	95% confidence interval	Significance level P
1.103	0.3121, 3.896	0.7481, not significant

**Table A192:** Odds ratio for the association of *Cryptosporidium* spp. and *Trichuris* spp.

	*Cryptosporidium* spp.	Non-*Cryptosporidium* spp.
*Trichuris* spp.	6	52
Non-*Trichuris* spp.	11	704
Odds ratio	95% confidence interval	Significance level P
7.385	2.626, 20.769	0.0009, significant

**Table A193:** Odds ratio for the association of *Cryptosporidium* spp. and *Schistosoma* spp.

	*Cryptosporidium* spp.	Non-*Cryptosporidium* spp.
*Schistosoma* spp.	1	3
Non-*Schistosoma* spp.	16	711
Odds ratio	95% confidence interval	Significance level P
14.813	1.460, 150.33	0.0900, not significant

**Table A194:** Odds ratio for the association of *Cryptosporidium* spp. and *Enterobius vermicularis.*

	*Cryptosporidium* spp.	Non-*Cryptosporidium* spp.
*Enterobius vermicularis*	0	31
Non-*Enterobius vermicularis*	17	725
Odds ratio	95% confidence interval	Significance level P
0.6580	0.03866, 11.200	1.0000, not significant

**Table A195:** Odds ratio for the association of *Cryptosporidium* spp. and *Tropheryma whipplei*.

	*Cryptosporidium* spp.	Non-*Cryptosporidium* spp.
*Tropheryma whipplei*	1	27
Non-*Tropheryma whipplei*	2	359
Odds ratio	95% confidence interval	Significance level P
6.648	0.5837, 75.721	0.2012, not significant

**Table A196:** Odds ratio for the association of *Cryptosporidium* spp. and enteropathogenic *Escherichia coli*.

	*Cryptosporidium* spp.	Non-*Cryptosporidium* spp.
Enteropathogenic *Escherichia coli*	4	206
Non-enteropathogenic *Escherichia coli*	9	346
Odds ratio	95% confidence interval	Significance level P
0.7465	0.2270, 2.455	0.7756, not significant

**Table A197:** Odds ratio for the association of *Cryptosporidium* spp. and enterotoxigenic *Escherichia coli*.

	*Cryptosporidium* spp.	Non-*Cryptosporidium* spp.
Enterotoxigenic *Escherichia coli*	6	131
Non-enterotoxigenic *Escherichia coli*	7	421
Odds ratio	95% confidence interval	Significance level P
2.755	0.9095, 8.343	0.0941, not significant

**Table A198:** Odds ratio for the association of *Cryptosporidium* spp. and enteroaggregative *Escherichia coli*.

	*Cryptosporidium* spp.	Non-*Cryptosporidium* spp.
Enteroaggregative *Escherichia coli*	5	172
Non-enteroaggregative *Escherichia coli*	8	380
Odds ratio	95% confidence interval	Significance level P
1.381	0.4451, 4.283	0.5572, not significant

**Table A199:** Odds ratio for the association of *Cryptosporidium* spp. and Shiga toxin-producing *Escherichia coli*.

	*Cryptosporidium* spp.	Non-*Cryptosporidium* spp.
Shiga toxin-producing *Escherichia coli*	0	20
Non-Shiga toxin-producing *Escherichia coli*	1	107
Odds ratio	95% confidence interval	Significance level P
1.748	0.06873, 44.457	1.0000, not significant

**Table A200:** Odds ratio for the association of *Cryptosporidium* spp. and *Dientamoeba fragilis*.

	*Cryptosporidium* spp.	Non-*Cryptosporidium* spp.
*Dientamoeba fragilis*	6	69
Non-*Dientamoeba fragilis*	11	687
Odds ratio	95% confidence interval	Significance level P
5.431	1.948, 15.141	0.0036, significant

**Table A201:** Odds ratio for the association of *Cryptosporidium* spp. and *Blastocystis* spp.

	*Cryptosporidium* spp.	Non-*Cryptosporidium* spp.
*Blastocystis* spp.	11	380
Non-*Blastocystis* spp.	6	376
Odds ratio	95% confidence interval	Significance level P
1.814	0.6639, 4.956	0.3274, not significant

**Table A202:** Odds ratio for the association of *Cryptosporidium* spp. and *Endolimax nana*.

	*Cryptosporidium* spp.	Non-*Cryptosporidium* spp.
*Endolimax nana*	4	212
Non-*Endolimax nana*	13	501
Odds ratio	95% confidence interval	Significance level P
0.7271	0.2343, 2.256	0.7891, not significant

**Table A203:** Odds ratio for the association of *Cryptosporidium* spp. and *Entamoeba coli*.

	*Cryptosporidium* spp.	Non-*Cryptosporidium* spp.
*Entamoeba coli*	4	223
Non-*Entamoeba coli*	13	489
Odds ratio	95% confidence interval	Significance level P
0.6747	0.2175, 2.093	0.6043, not significant

**Table A204:** Odds ratio for the association of *Cryptosporidium* spp. and *Entamoeba dispar*.

	*Cryptosporidium* spp.	Non-*Cryptosporidium* spp.
*Entamoeba dispar*	3	115
Non-*Entamoeba dispar*	14	598
Odds ratio	95% confidence interval	Significance level P
1.114	0.3151, 3.941	0.7460, not significant

**Table A205:** Odds ratio for the association of *Cryptosporidium* spp. and *Iodamoeba buetschlii*.

	*Cryptosporidium* spp.	Non-*Cryptosporidium* spp.
*Iodamoeba buetschlii*	0	55
Non-*Iodamoeba buetschlii*	17	658
Odds ratio	95% confidence interval	Significance level P
0.3390	0.2010, 5.717	0.6311, not significant

**Table A206:** Odds ratio for the association of *Cryptosporidium* spp. and *Pentatrichomonas hominis*.

	*Cryptosporidium* spp.	Non-*Cryptosporidium* spp.
*Pentatrichomonas hominis*	0	3
Non-*Pentatrichomonas hominis*	17	709
Odds ratio	95% confidence interval	Significance level P
5.792	0.2879, 116.52	1.0000, not significant

**Table A207:** Odds ratio for the association of *Cryptosporidium* spp. and *Encephalocytozoon* spp.

	*Cryptosporidium* spp.	Non-*Cryptosporidium* spp.
*Encephalocytozoon* spp.	0	7
Non-*Encephalocytozoon* spp.	11	269
Odds ratio	95% confidence interval	Significance level P
1.562	0.08396, 29.073	1.0000, not significant

**Table A208:** Odds ratio for the association of *Cryptosporidium* spp. and *Aeromonas* spp.

	*Cryptosporidium* spp.	Non-*Cryptosporidium* spp.
*Aeromonas* spp.	0	5
Non-*Aeromonas* spp.	11	271
Odds ratio	95% confidence interval	Significance level P
2.146	0.1117, 41.225	1.0000, not significant

**Table A209:** Odds ratio for the association of *Cryptosporidium* spp. and conidia.

	*Cryptosporidium* spp.	Non-*Cryptosporidium* spp.
Conidia	0	41
Non-conidia	11	193
Odds ratio	95% confidence interval	Significance level P
0.2027	0.01170, 3.511	0.2193, not significant

**Table A210:** Odds ratio for the association of *Cryptosporidium* spp. and pseudoconidia.

	*Cryptosporidium* spp.	Non-*Cryptosporidium* spp.
Pseudoconidia	1	26
Non-pseudoconidia	10	208
Odds ratio	95% confidence interval	Significance level P
0.8000	0.09834, 6.508	1.0000, not significant

**Table A211:** Odds ratio for the association of *Ascaris* spp. and *Necator americanus*.

	*Ascaris* spp.	Non-*Ascaris* spp.
*Necator americanus*	37	49
Non-*Necator americanus*	93	594
Odds ratio	95% confidence interval	Significance level P
4.823	2.985, 7.792	<0.0001, not significant

**Table A212:** Odds ratio for the association of *Ascaris* spp. and *Strongyloides* spp.

	*Ascaris* spp.	Non-*Ascaris* spp.
*Strongyloides* spp.	26	13
Non-*Strongyloides* spp.	104	630
Odds ratio	95% confidence interval	Significance level P
12.115	6.031, 24.337	<0.0001, significant

**Table A213:** Odds ratio for the association of *Ascaris* spp. and *Taenia* spp.

	*Ascaris* spp.	Non-*Ascaris* spp.
*Taenia* spp.	10	23
Non-*Taenia* spp.	120	620
Odds ratio	95% confidence interval	Significance level P
2.246	1.042, 4.841	0.0526, not significant

**Table A214:** Odds ratio for the association of *Ascaris* spp. and *Hymenolepis* spp.

	*Ascaris* spp.	Non-*Ascaris* spp.
*Hymenolepis* spp.	18	108
Non-*Hymenolepis* spp.	112	535
Odds ratio	95% confidence interval	Significance level P
0.7961	0.4644, 1.365	0.4381, not significant

**Table A215:** Odds ratio for the association of *Ascaris* spp. and *Trichuris* spp.

	*Ascaris* spp.	Non-*Ascaris* spp.
*Trichuris* spp.	76	82
Non-*Trichuris* spp.	54	561
Odds ratio	95% confidence interval	Significance level P
9.629	6.334, 14.638	<0.0001, significant

**Table A216:** Odds ratio for the association of *Ascaris* spp. and *Schistosoma* spp.

	*Ascaris* spp.	Non-*Ascaris* spp.
*Schistosoma* spp.	1	3
Non-*Schistosoma* spp.	126	601
Odds ratio	95% confidence interval	Significance level P
1.590	0.1640, 15.418	0.5347, not significant

**Table A217:** Odds ratio for the association of *Ascaris* spp. and *Enterobius vermicularis*.

	*Ascaris* spp.	Non-*Ascaris* spp.
*Enterobius vermicularis*	11	20
Non-*Enterobius vermicularis*	119	623
Odds ratio	95% confidence interval	Significance level P
2.879	1.344, 6.167	0.0111, significant

**Table A218:** Odds ratio for the association of *Ascaris* spp. and *Tropheryma whipplei*.

	*Ascaris* spp.	Non-*Ascaris* spp.
*Tropheryma whipplei*	2	26
Non-*Tropheryma whipplei*	44	317
Odds ratio	95% confidence interval	Significance level P
0.5542	0.1271, 2.417	0.5564, not significant

**Table A219:** Odds ratio for the association of *Ascaris* spp. and enteropathogenic *Escherichia coli*.

	*Ascaris* spp.	Non-*Ascaris* spp.
Enteropathogenic *Escherichia coli*	45	165
Non-enteropathogenic *Escherichia coli*	72	283
Odds ratio	95% confidence interval	Significance level P
0.7483	0.7050, 1.630	0.7483, not significant

**Table A220:** Odds ratio for the association of *Ascaris* spp. and enterotoxigenic *Escherichia coli*.

	*Ascaris* spp.	Non-*Ascaris* spp.
Enterotoxigenic *Escherichia coli*	34	103
Non-enterotoxigenic *Escherichia coli*	83	345
Odds ratio	95% confidence interval	Significance level P
1.372	0.8698, 2.164	0.1835, not significant

**Table A221:** Odds ratio for the association of *Ascaris* spp. and enteroaggregative *Escherichia coli*.

	*Ascaris* spp.	Non-*Ascaris* spp.
Enteroaggregative *Escherichia coli*	27	150
Non-enteroaggregative *Escherichia coli*	80	308
Odds ratio	95% confidence interval	Significance level P
0.6930	0.4297, 1.118	0.1645, not significant

**Table A222:** Odds ratio for the association of *Ascaris* spp. and Shiga toxin-producing *Escherichia coli*.

	*Ascaris* spp.	Non-*Ascaris* spp.
Shiga toxin-producing *Escherichia coli*	2	18
Non-Shiga toxin-producing *Escherichia coli*	17	91
Odds ratio	95% confidence interval	Significance level P
0.5948	0.1262, 2.803	0.7357, not significant

**Table A223:** Odds ratio for the association of *Ascaris* spp. and *Dientamoeba fragilis*.

	*Ascaris* spp.	Non-*Ascaris* spp.
*Dientamoeba fragilis*	37	38
Non-*Dientamoeba fragilis*	91	607
Odds ratio	95% confidence interval	Significance level P
6.495	3.925, 10.746	<0.0001, significant

**Table A224:** Odds ratio for the association of *Ascaris* spp. and *Blastocystis* spp.

	*Ascaris* spp.	Non-*Ascaris* spp.
*Blastocystis* spp.	80	311
Non-*Blastocystis* spp.	50	332
Odds ratio	95% confidence interval	Significance level P
1.708	1.161, 2.512	0.0070, significant

**Table A225:** Odds ratio for the association of *Ascaris* spp. and *Endolimax nana*.

	*Ascaris* spp.	Non-*Ascaris* spp.
*Endolimax nana*	43	173
Non-*Endolimax nana*	84	430
Odds ratio	95% confidence interval	Significance level P
1.272	0.8463, 1.913	0.2847, not significant

**Table A226:** Odds ratio for the association of *Ascaris* spp. and *Entamoeba coli*.

	*Ascaris* spp.	Non-*Ascaris* spp.
*Entamoeba coli*	45	182
Non-*Entamoeba coli*	82	420
Odds ratio	95% confidence interval	Significance level P
1.266	0.8462, 1.895	0.2483, not significant

**Table A227:** Odds ratio for the association of *Ascaris* spp. and *Entamoeba dispar*.

	*Ascaris* spp.	Non-*Ascaris* spp.
*Entamoeba dispar*	36	82
Non-*Entamoeba dispar*	91	521
Odds ratio	95% confidence interval	Significance level P
2.514	1.601, 3.945	0.0001, significant

**Table A228:** Odds ratio for the association of *Ascaris* spp. and *Iodamoeba buetschlii*.

	*Ascaris* spp.	Non-*Ascaris* spp.
*Iodamoeba buetschlii*	9	46
Non-*Iodamoeba buetschlii*	118	557
Odds ratio	95% confidence interval	Significance level P
0.9235	0.4399, 1.939	1.0000, not significant

**Table A229:** Odds ratio for the association of *Ascaris* spp. and *Pentatrichomonas hominis*.

	*Ascaris* spp.	Non-*Ascaris* spp.
*Pentatrichomonas hominis*	0	3
Non-*Pentatrichomonas hominis*	126	600
Odds ratio	95% confidence interval	Significance level P
0.6781	0.03479, 13.220	1.0000, not significant

**Table A230:** Odds ratio for the association of *Ascaris* spp. and *Encephalocytozoon* spp.

	*Ascaris* spp.	Non-*Ascaris* spp.
*Encephalocytozoon* spp.	1	6
Non-*Encephalocytozoon* spp.	76	204
Odds ratio	95% confidence interval	Significance level P
0.4474	0.05296, 3.779	0.6789, not significant

**Table A231:** Odds ratio for the association of *Ascaris* spp. and *Aeromonas* spp.

	*Ascaris* spp.	Non-*Ascaris* spp.
*Aeromonas* spp.	2	3
Non-*Aeromonas* spp.	75	207
Odds ratio	95% confidence interval	Significance level P
1.840	0.3014, 11.232	0.6130, not significant

**Table A232:** Odds ratio for the association of *Ascaris* spp. and conidia.

	*Ascaris* spp.	Non-*Ascaris* spp.
Conidia	12	29
Non-conidia	62	142
Odds ratio	95% confidence interval	Significance level P
0.9477	0.4539, 1.979	1.0000, not significant

**Table A233:** Odds ratio for the association of *Ascaris* spp. and pseudoconidia.

	*Ascaris* spp.	Non-*Ascaris* spp.
Pseudoconidia	9	18
Non-pseudoconidia	65	153
Odds ratio	95% confidence interval	Significance level P
1.177	0.5024, 2.757	0.8244, not significant

**Table A234:** Odds ratio for the association of *Necator americanus* and *Strongyloides* spp.

	*Necator americanus*	Non-*Necator americanus*
*Strongyloides* spp.	10	29
Non-*Strongyloides* spp.	76	658
Odds ratio	95% confidence interval	Significance level P
2.985	1.400, 6.366	0.0072, significant

**Table A235:** Odds ratio for the association of *Necator americanus* and *Taenia* spp.

	*Necator americanus*	Non-*Necator americanus*
*Taenia* spp.	5	28
Non-*Taenia* spp.	81	659
Odds ratio	95% confidence interval	Significance level P
1.453	0.5456, 3.869	0.4008, not significant

**Table A236:** Odds ratio for the association of *Necator americanus* and *Hymenolepis* spp.

	*Necator americanus*	Non-*Necator americanus*
*Hymenolepis* spp.	18	108
Non-*Hymenolepis* spp.	68	579
Odds ratio	95% confidence interval	Significance level P
1.419	0.8116, 2.481	0.2172, not significant

**Table A237:** Odds ratio for the association of *Necator americanus* and *Trichuris* spp.

	*Necator americanus*	Non-*Necator americanus*
*Trichuris* spp.	40	118
Non-*Trichuris* spp.	46	569
Odds ratio	95% confidence interval	Significance level P
4.193	2.626, 6.695	<0.0001, significant

**Table A238:** Odds ratio for the association of *Necator americanus* and *Schistosoma* spp.

	*Necator americanus*	Non-*Necator americanus*
*Schistosoma* spp.	1	3
Non-*Schistosoma* spp.	83	644
Odds ratio	95% confidence interval	Significance level P
2.586	0.2658, 25.166	0.3870, not significant

**Table A239:** Odds ratio for the association of *Necator americanus* and *Enterobius vermicularis*.

	*Necator americanus*	Non-*Necator americanus*
*Enterobius vermicularis*	5	26
Non-*Enterobius vermicularis*	81	661
Odds ratio	95% confidence interval	Significance level P
1.569	0.5862, 4.201	0.3766, not significant

**Table A240:** Odds ratio for the association of *Necator americanus* and *Tropheryma whipplei*.

	*Necator americanus*	Non-*Necator americanus*
*Tropheryma whipplei*	2	26
Non-*Tropheryma whipplei*	50	311
Odds ratio	95% confidence interval	Significance level P
0.4785	0.1101, 2.079	0.5611, not significant

**Table A241:** Odds ratio for the association of *Necator americanus* and enteropathogenic *Escherichia coli*.

	*Necator americanus*	Non-*Necator americanus*
Enteropathogenic *Escherichia coli*	32	178
Non-enteropathogenic *Escherichia coli*	49	306
Odds ratio	95% confidence interval	Significance level P
1.123	0.6930, 1.819	0.7069, not significant

**Table A242:** Odds ratio for the association of *Necator americanus* and enterotoxigenic *Escherichia coli*.

	*Necator americanus*	Non-*Necator americanus*
Enterotoxigenic *Escherichia coli*	28	109
Non-enterotoxigenic *Escherichia coli*	53	375
Odds ratio	95% confidence interval	Significance level P
1.818	1.097, 3.012	0.0246, significant

**Table A243:** Odds ratio for the association of *Necator americanus* and enteroaggregative *Escherichia coli*.

	*Necator americanus*	Non-*Necator americanus*
Enteroaggregative *Escherichia coli*	33	144
Non-enteroaggregative *Escherichia coli*	48	340
Odds ratio	95% confidence interval	Significance level P
1.623	1.000, 2.635	0.0528, not significant

**Table A244:** Odds ratio for the association of *Necator americanus* and Shiga toxin-producing *Escherichia coli*.

	*Necator americanus*	Non-*Necator americanus*
Shiga toxin-producing *Escherichia coli*	4	16
Non-Shiga toxin-producing *Escherichia coli*	25	83
Odds ratio	95% confidence interval	Significance level P
0.8300	0.2541, 2.711	1.0000, not significant

**Table A245:** Odds ratio for the association of *Necator americanus* and *Dientamoeba fragilis*.

	*Necator americanus*	Non-*Necator americanus*
*Dientamoeba fragilis*	*15*	*60*
Non-*Dientamoeba fragilis*	*71*	*627*
Odds ratio	95% confidence interval	Significance level P
2.208	1.191, 4.091	0.0184, significant

**Table A246:** Odds ratio for the association of *Necator americanus* and *Blastocystis* spp.

	*Necator americanus*	Non-*Necator americanus*
*Blastocystis* spp.	40	351
Non-*Blastocystis* spp.	46	336
Odds ratio	95% confidence interval	Significance level P
0.8324	0.5310, 1.305	0.4264, not significant

**Table A247:** Odds ratio for the association of *Necator americanus* and *Endolimax nana*.

	*Necator americanus*	Non-*Necator americanus*
*Endolimax nana*	26	190
Non-*Endolimax nana*	58	456
Odds ratio	95% confidence interval	Significance level P
1.076	0.6573, 1.761	0.7997, not significant

**Table A248:** Odds ratio for the association of *Necator americanus* and *Entamoeba coli*.

	*Necator americanus*	Non-*Necator americanus*
*Entamoeba coli*	21	206
Non-*Entamoeba coli*	63	439
Odds ratio	95% confidence interval	Significance level P
0.7104	0.4219, 1.196	0.2124, not significant

**Table A249:** Odds ratio for the association of *Necator americanus* and *Entamoeba dispar*.

	*Necator americanus*	Non-*Necator americanus*
*Entamoeba dispar*	14	104
Non-*Entamoeba dispar*	70	542
Odds ratio	95% confidence interval	Significance level P
1.042	0.5657, 1.920	0.8753, not significant

**Table A250:** Odds ratio for the association of *Necator americanus* and *Iodamoeba buetschlii*.

	*Necator americanus*	Non-*Necator americanus*
*Iodamoeba buetschlii*	4	51
Non-*Iodamoeba buetschlii*	80	595
Odds ratio	95% confidence interval	Significance level P
0.5833	0.2053, 1.658	0.3842, not significant

**Table A251:** Odds ratio for the association of *Necator americanus* and *Pentatrichomonas hominis*.

	*Necator americanus*	Non-*Necator americanus*
*Pentatrichomonas hominis*	0	3
Non-*Pentatrichomonas hominis*	83	643
Odds ratio	95% confidence interval	Significance level P
1.101	0.05633, 21.517	1.0000, not significant

**Table A252:** Odds ratio for the association of *Necator americanus* and *Encephalocytozoon* spp.

	*Necator americanus*	Non-*Necator americanus*
*Encephalocytozoon* spp.	0	7
Non-*Encephalocytozoon* spp.	32	248
Odds ratio	95% confidence interval	Significance level P
0.5097	0.02842, 9.142	1.0000, not significant

**Table A253:** Odds ratio for the association of *Necator americanus* and *Aeromonas* spp.

	*Necator americanus*	Non-*Necator americanus*
*Aeromonas* spp.	0	5
Non-*Aeromonas* spp.	32	250
Odds ratio	95% confidence interval	Significance level P
0.7007	0.03784, 12.976	1.0000, not significant

**Table A254:** Odds ratio for the association of *Necator americanus* and conidia.

	*Necator americanus*	Non-*Necator americanus*
Conidia	4	37
Non-conidia	26	178
Odds ratio	95% confidence interval	Significance level P
0.7401	0.2437, 2.248	0.7950, not significant

**Table A255:** Odds ratio for the association of *Necator americanus* and pseudoconidia.

	*Necator americanus*	Non-*Necator americanus*
Pseudoconidia	2	25
Non-pseudoconidia	28	190
Odds ratio	95% confidence interval	Significance level P
0.5429	0.1218, 2.419	0.5466, not significant

**Table A256:** Odds ratio for the association of *Strongyloides* spp. and *Taenia* spp.

	*Strongyloides* spp.	Non-*Strongyloides* spp.
*Taenia* spp.	3	30
Non-*Taenia* spp.	36	704
Odds ratio	95% confidence interval	Significance level P
1.956	0.5695, 6.714	0.2289, not significant

**Table A257:** Odds ratio for the association of *Strongyloides* spp. and *Hymenolepis* spp.

	*Strongyloides* spp.	Non-*Strongyloides* spp.
*Hymenolepis* spp.	6	120
Non-*Hymenolepis* spp.	33	614
Odds ratio	95% confidence interval	Significance level P
0.9303	0.3813, 2.269	1.0000, not significant

**Table A258:** Odds ratio for the association of *Strongyloides* spp. and *Trichuris* spp.

	*Strongyloides* spp.	Non-*Strongyloides* spp.
*Trichuris* spp.	27	131
Non-*Trichuris* spp.	12	603
Odds ratio	95% confidence interval	Significance level P
10.357	5.113, 20.980	<0.0001, significant

**Table A259:** Odds ratio for the association of *Strongyloides* spp. and *Schistosoma* spp.

	*Strongyloides* spp.	Non-*Strongyloides* spp.
*Schistosoma* spp.	1	3
Non-*Schistosoma* spp.	37	690
Odds ratio	95% confidence interval	Significance level P
6.216	0.6309, 61.248	0.1926, not significant

**Table A260:** Odds ratio for the association of *Strongyloides* spp. and *Enterobius vermicularis*.

	*Strongyloides* spp.	Non-*Strongyloides* spp.
*Enterobius vermicularis*	4	27
Non-*Enterobius vermicularis*	35	707
Odds ratio	95% confidence interval	Significance level P
2.993	0.9923, 9.025	0.0650, not significant

**Table A261:** Odds ratio for the association of *Strongyloides* spp. and *Tropheryma whipplei*.

	*Strongyloides* spp.	Non-*Strongyloides* spp.
*Tropheryma whipplei*	0	28
Non-*Tropheryma whipplei*	9	352
Odds ratio	95% confidence interval	Significance level P
0.6510	0.03691, 11.481	1.0000, not significant

**Table A262:** Odds ratio for the association of *Strongyloides* spp. and enteropathogenic *Escherichia coli*.

	*Strongyloides* spp.	Non-*Strongyloides* spp.
Enteropathogenic *Escherichia coli*	16	194
Non-enteropathogenic *Escherichia coli*.	22	333
Odds ratio	95% confidence interval	Significance level P
1.248	0.6401, 2.435	0.6025, not significant

**Table A263:** Odds ratio for the association of *Strongyloides* spp. and enterotoxigenic *Escherichia coli*.

	*Strongyloides* spp.	Non-*Strongyloides* spp.
Enterotoxigenic *Escherichia coli*	11	126
Non-enterotoxigenic *Escherichia coli*	27	401
Odds ratio	95% confidence interval	Significance level P
1.297	0.6253, 2.689	0.5562, not significant

**Table A264:** Odds ratio for the association of *Strongyloides* spp. and enteroaggregative *Escherichia coli*.

	*Strongyloides* spp.	Non-*Strongyloides* spp.
Enteroaggregative *Escherichia coli*	14	163
Non-enteroaggregative *Escherichia coli*	24	364
Odds ratio	95% confidence interval	Significance level P
1.303	0.6569, 2.583	0.4709, not significant

**Table A265:** Odds ratio for the association of *Strongyloides* spp. and Shiga toxin-producing *Escherichia coli*.

	*Strongyloides* spp.	Non-*Strongyloides* spp.
Shiga toxin-producing *Escherichia coli*	0	4
Non-Shiga toxin-producing *Escherichia coli*	20	104
Odds ratio	95% confidence interval	Significance level P
0.5664	0.02933, 10.937	1.0000, not significant

**Table A266:** Odds ratio for the association of *Strongyloides* spp. and *Dientamoeba fragilis*.

	*Strongyloides* spp.	Non-*Strongyloides* spp.
*Dientamoeba fragilis*	18	57
Non-*Dientamoeba fragilis*	21	677
Odds ratio	95% confidence interval	Significance level P
10.180	5.130, 20.202	<0.0001, significant

**Table A267:** Odds ratio for the association of *Strongyloides* spp. and *Blastocystis* spp.

	*Strongyloides* spp.	Non-*Strongyloides* spp.
*Blastocystis* spp.	29	362
Non-*Blastocystis* spp.	10	372
Odds ratio	95% confidence interval	Significance level P
2.980	1.431, 6.205	0.0027, significant

**Table A268:** Odds ratio for the association of *Strongyloides* spp. and *Endolimax nana*.

	*Strongyloides* spp.	Non-*Strongyloides* spp.
*Endolimax nana*	16	200
Non-*Endolimax nana*	22	492
Odds ratio	95% confidence interval	Significance level P
1.789	0.9203, 3.478	0.0996, not significant

**Table A269:** Odds ratio for the association of *Strongyloides* spp. and *Entamoeba coli*.

	*Strongyloides* spp.	Non-*Strongyloides* spp.
*Entamoeba coli*	18	209
Non-*Entamoeba coli*	20	482
Odds ratio	95% confidence interval	Significance level P
2.076	1.076, 4.005	0.0312, significant

**Table A270:** Odds ratio for the association of *Strongyloides* spp. and *Entamoeba dispar*.

	*Strongyloides* spp.	Non-*Strongyloides* spp.
*Entamoeba dispar*	15	103
Non-*Entamoeba dispar*	23	589
Odds ratio	95% confidence interval	Significance level P
3.729	1.883, 7.387	0.0003, significant

**Table A271:** Odds ratio for the association of *Strongyloides* spp. and *Iodamoeba buetschlii*.

	*Strongyloides* spp.	Non-*Strongyloides* spp.
*Iodamoeba buetschlii*	2	53
Non-*Iodamoeba buetschlii*	38	637
Odds ratio	95% confidence interval	Significance level P
0.6326	0.1484, 2.696	0.7605, not significant

**Table A272:** Odds ratio for the association of *Strongyloides* spp. and *Pentatrichomonas hominis*.

	*Strongyloides* spp.	Non-*Strongyloides* spp.
*Pentatrichomonas hominis*	0	3
Non-*Pentatrichomonas hominis*	38	688
Odds ratio	95% confidence interval	Significance level P
2.555	0.1295, 50.380	1.0000, not significant

**Table A273:** Odds ratio for the association of *Strongyloides* spp. and *Encephalocytozoon* spp.

	*Strongyloides* spp.	Non-*Strongyloides* spp.
*Encephalocytozoon* spp.	0	7
Non-*Encephalocytozoon* spp..	30	250
Odds ratio	95% confidence interval	Significance level P
0.5475	0.03049, 9.832	1.0000, not significant

**Table A274:** Odds ratio for the association of *Strongyloides* spp. and *Aeromonas* spp.

	*Strongyloides* spp.	Non-*Strongyloides* spp.
*Aeromonas* spp.	0	5
Non-*Aeromonas* spp.	30	252
Odds ratio	95% confidence interval	Significance level P
0.7526	0.04059, 13.955	1.0000, not significant

**Table A275:** Odds ratio for the association of *Strongyloides* spp. and conidia.

	*Strongyloides* spp.	Non-*Strongyloides* spp.
Conidia	3	38
Non-conidia	26	178
Odds ratio	95% confidence interval	Significance level P
0.5405	0.1555, 1.878	0.4326, not significant

**Table A276:** Odds ratio for the association of *Strongyloides* spp. and pseudoconidia.

	*Strongyloides* spp.	Non-*Strongyloides* spp.
Pseudoconidia	5	22
Non-pseudoconidia	24	194
Odds ratio	95% confidence interval	Significance level P
1.837	0.6366, 5.302	0.3373, not significant

**Table A277:** Odds ratio for the association of *Taenia* spp. and *Hymenolepis* spp.

	*Taenia* spp.	Non-*Taenia* spp.
*Hymenolepis* spp.	9	117
Non-*Hymenolepis* spp.	24	623
Odds ratio	95% confidence interval	Significance level P
1.997	0.9050, 4.406	0.0915, not significant

**Table A278:** Odds ratio for the association of *Taenia* spp. and *Trichuris* spp.

	*Taenia* spp.	Non-*Taenia* spp.
*Trichuris* spp.	14	144
Non-*Trichuris* spp.	19	596
Odds ratio	95% confidence interval	Significance level P
3.050	1.493, 6.229	0.0032, significant

**Table A279:** Odds ratio for the association of *Taenia* spp. and *Schistosoma* spp.

	*Taenia* spp.	Non-*Taenia* spp.
*Schistosoma* spp.	1	3
Non-*Schistosoma* spp.	32	695
Odds ratio	95% confidence interval	Significance level P
7.240	0.7322, 71.584	0.1690, not significant

**Table A280:** Odds ratio for the association of *Taenia* spp. and *Enterobius vermicularis*.

	*Taenia* spp.	Non-*Taenia* spp.
*Enterobius vermicularis*	3	28
Non-*Enterobius vermicularis*	30	712
Odds ratio	95% confidence interval	Significance level P
2.543	0.7316, 8.838	0.1413, not significant

**Table A281:** Odds ratio for the association of *Taenia* spp. and *Tropheryma whipplei*.

	*Taenia* spp.	Non-*Taenia* spp.
*Tropheryma whipplei*	0	28
Non-*Tropheryma whipplei*	14	347
Odds ratio	95% confidence interval	Significance level P
0.4204	0.02443, 7.237	0.6115, not significant

**Table A282:** Odds ratio for the association of *Taenia* spp. and enteropathogenic *Escherichia coli*.

	*Taenia* spp.	Non-*Taenia* spp.
Enteropathogenic *Escherichia coli*	14	196
Non-enteropathogenic *Escherichia coli*	17	338
Odds ratio	95% confidence interval	Significance level P
1.420	0.6850, 2.944	0.3458, not significant

**Table A283:** Odds ratio for the association of *Taenia* spp. and enterotoxigenic *Escherichia coli*.

	*Taenia* spp.	Non-*Taenia* spp.
Enterotoxigenic *Escherichia coli*	10	127
Non-enterotoxigenic *Escherichia coli*	21	407
Odds ratio	95% confidence interval	Significance level P
1.526	0.7002, 3.326	0.2853, not significant

**Table A284:** Odds ratio for the association of *Taenia* spp. and enteroaggregative *Escherichia coli*.

	*Taenia* spp.	Non-*Taenia* spp.
Enteroaggregative *Escherichia coli*	8	169
Non-enteroaggregative *Escherichia coli*	23	365
Odds ratio	95% confidence interval	Significance level P
0.7512	0.3292, 1.714	0.5562, not significant

**Table A285:** Odds ratio for the association of *Taenia* spp. and Shiga toxin-producing *Escherichia coli*.

	*Taenia* spp.	Non-*Taenia* spp.
Shiga toxin-producing *Escherichia coli*	1	19
Non-Shiga toxin-producing *Escherichia coli*	6	102
Odds ratio	95% confidence interval	Significance level P
0.8947	0.1018, 7.863	1.0000, not significant

**Table A286:** Odds ratio for the association of *Taenia* spp. and *Dientamoeba fragilis*.

	*Taenia* spp.	Non-*Taenia* spp.
*Dientamoeba fragilis*	7	68
Non-*Dientamoeba fragilis*	26	672
Odds ratio	95% confidence interval	Significance level P
2.661	1.113, 6.359	0.0329, significant

**Table A287:** Odds ratio for the association of *Taenia* spp. and *Blastocystis* spp.

	*Taenia* spp.	Non-*Taenia* spp.
*Blastocystis* spp.	16	375
Non *Blastocystis* spp.	15	367
Odds ratio	95% confidence interval	Significance level P
1.044	0.5086, 2.143	1.0000, not significant

**Table A288:** Odds ratio for the association of *Taenia* spp. and *Endolimax nana*.

	*Taenia* spp.	Non-*Taenia* spp.
*Endolimax nana*	13	203
Non-*Endolimax nana*	20	494
Odds ratio	95% confidence interval	Significance level P
1.582	0.7720, 3.241	0.2411, not significant

**Table A289:** Odds ratio for the association of *Taenia* spp. and *Entamoeba coli*.

	*Taenia* spp.	Non-*Taenia* spp.
*Entamoeba coli*	12	125
Non-*Entamoeba coli*	21	571
Odds ratio	95% confidence interval	Significance level P
2.610	1.251, 5.446	0.0195, significant

**Table A290:** Odds ratio for the association of *Taenia* spp. and *Entamoeba dispar*.

	*Taenia* spp.	Non-*Taenia* spp.
*Entamoeba dispar*	7	111
Non-*Entamoeba dispar*	26	586
Odds ratio	95% confidence interval	Significance level P
1.421	0.6020, 3.356	0.4652, not significant

**Table A291:** Odds ratio for the association of *Taenia* spp. and *Iodamoeba buetschlii*.

	*Taenia* spp.	Non-*Taenia* spp.
*Iodamoeba buetschlii*	1	54
Non-*Iodamoeba buetschlii*	32	643
Odds ratio	95% confidence interval	Significance level P
0.3721	0–04985, 2.778	0.5032, not significant

**Table A292:** Odds ratio for the association of *Taenia* spp. and *Pentatrichomonas hominis*.

	*Taenia* spp.	Non-*Taenia* spp.
*Pentatrichomonas hominis*	0	3
Non-*Pentatrichomonas hominis*	33	693
Odds ratio	95% confidence interval	Significance level P
2.957	0.1496, 58.465	1.0000, not significant

**Table A293:** Odds ratio for the association of *Taenia* spp. and *Encephalocytozoon* spp.

	*Taenia* spp.	Non-*Taenia* spp.
*Encephalocytozoon* spp.	0	7
Non-*Encephalocytozoon* spp.	19	261
Odds ratio	95% confidence interval	Significance level P
0.8940	0.04918, 16.251	1.0000, not significant

**Table A294:** Odds ratio for the association of *Taenia* spp. and *Aeromonas* spp.

	*Taenia* spp.	Non-*Taenia* spp.
*Aeromonas* spp.	0	5
Non-*Aeromonas* spp.	19	263
Odds ratio	95% confidence interval	Significance level P
1.228	0.06546, 23.054	1.0000, not significant

**Table A295:** Odds ratio for the association of *Taenia* spp. and conidia.

	*Taenia* spp.	Non-*Taenia* spp.
Conidia	4	37
Non-conidia	15	189
Odds ratio	95% confidence interval	Significance level P
1.362	0.4278, 4.337	0.5340, not significant

**Table A296:** Odds ratio for the association of *Taenia* spp. and pseudoconidia.

	*Taenia* spp.	Non-*Taenia* spp.
Pseudoconidia	5	22
Non-pseudoconidia	14	204
Odds ratio	95% confidence interval	Significance level P
3.312	1.089, 10.070	0.0434, significant

**Table A297:** Odds ratio for the association of *Hymenolepis* spp. and *Trichuris* spp.

	*Hymenolepis* spp.	Non-*Hymenolepis* spp.
*Trichuris* spp.	36	122
Non-*Trichuris* spp.	90	525
Odds ratio	95% confidence interval	Significance level P
1.721	1.115, 2.656	0.0158, significant

**Table A298:** Odds ratio for the association of *Hymenolepis* spp. and *Schistosoma* spp.

	*Hymenolepis* spp.	Non-*Hymenolepis* spp.
*Schistosoma* spp.	1	3
Non-*Schistosoma* spp.	122	605
Odds ratio	95% confidence interval	Significance level P
1.653	0.1704, 16.033	0.5222, not significant

**Table A299:** Odds ratio for the association of *Hymenolepis* spp. and *Enterobius vermicularis*.

	*Hymenolepis* spp.	Non-*Hymenolepis* spp.
*Enterobius vermicularis*	7	24
Non-*Enterobius vermicularis*	119	623
Odds ratio	95% confidence interval	Significance level P
1.527	0.6431, 3.625	0.3232, not significant

**Table A300:** Odds ratio for the association of *Hymenolepis* spp. and *Tropheryma whipplei*.

	*Hymenolepis* spp.	Non-*Hymenolepis* spp.
*Tropheryma whipplei*	5	23
Non-*Tropheryma whipplei*	76	285
Odds ratio	95% confidence interval	Significance level P
0.8152	0.2999, 2.216	0.8125, not significant

**Table A301:** Odds ratio for the association of *Hymenolepis* spp. and enteropathogenic *Escherichia coli*.

	*Hymenolepis* spp.	Non-*Hymenolepis* spp.
Enteropathogenic *Escherichia coli*	44	166
Non-enteropathogenic *Escherichia coli*	69	286
Odds ratio	95% confidence interval	Significance level P
1.099	0.7191, 1.679	0.6648, not significant

**Table A302:** Odds ratio for the association of *Hymenolepis* spp. and enterotoxigenic *Escherichia coli*.

	*Hymenolepis* spp.	Non-*Hymenolepis* spp.
Enterotoxigenic *Escherichia coli*	33	104
Non-enterotoxigenic *Escherichia coli*	80	348
Odds ratio	95% confidence interval	Significance level P
1.380	0.8705, 2.189	0.1781, not significant

**Table A303:** Odds ratio for the association of *Hymenolepis* spp. and enteroaggregative *Escherichia coli*.

	*Hymenolepis* spp.	Non-*Hymenolepis* spp.
Enteroaggregative *Escherichia coli*	32	145
Non-enteroaggregative *Escherichia coli*	81	307
Odds ratio	95% confidence interval	Significance level P
0.8364	0.5309, 1.318	0.4968, not significant

**Table A304:** Odds ratio for the association of *Hymenolepis* spp. and Shiga toxin-producing *Escherichia coli*.

	*Hymenolepis* spp.	Non-*Hymenolepis* spp.
Shiga toxin-producing *Escherichia coli*	6	14
Non-Shiga toxin-producing *Escherichia coli*	27	81
Odds ratio	95% confidence interval	Significance level P
1.286	0.4494, 3.678	0.7811, not significant

**Table A305:** Odds ratio for the association of *Hymenolepis* spp. and *Dientamoeba fragilis*.

	*Hymenolepis* spp.	Non-*Hymenolepis* spp.
*Dientamoeba fragilis*	9	66
Non-*Dientamoeba fragilis*	107	591
Odds ratio	95% confidence interval	Significance level P
0.7532	0.3643, 1.557	0.5010, not significant

**Table A306:** Odds ratio for the association of *Hymenolepis* spp. and *Blastocystis* spp.

	*Hymenolepis* spp.	Non-*Hymenolepis* spp.
*Blastocystis* spp.	44	347
Non-*Blastocystis* spp.	62	320
Odds ratio	95% confidence interval	Significance level P
0.6545	0.4321, 0.9913	0.0473, significant

**Table A307:** Odds ratio for the association of *Hymenolepis* spp. and *Endolimax nana*.

	*Hymenolepis* spp.	Non-*Hymenolepis* spp.
*Endolimax nana*	31	185
Non-*Endolimax nana*	92	422
Odds ratio	95% confidence interval	Significance level P
0.7686	0.4939, 1.196	0.2789, not significant

**Table A308:** Odds ratio for the association of *Hymenolepis* spp. and *Entamoeba coli*.

	*Hymenolepis* spp.	Non-*Hymenolepis* spp.
*Entamoeba coli*	34	193
Non-*Entamoeba coli*	89	413
Odds ratio	95% confidence interval	Significance level P
0.8175	0.5314, 1.257	0.3989, not significant

**Table A309:** Odds ratio for the association of *Hymenolepis* spp. and *Entamoeba dispar*.

	*Hymenolepis* spp.	Non-*Hymenolepis* spp.
*Entamoeba dispar*	20	98
Non-*Entamoeba dispar*	103	509
Odds ratio	95% confidence interval	Significance level P
1.009	0.5962, 1.706	1.0000, not significant

**Table A310:** Odds ratio for the association of *Hymenolepis* spp. and *Iodamoeba buetschlii*.

	*Hymenolepis* spp.	Non-*Hymenolepis* spp.
*Iodamoeba buetschlii*	4	51
Non-*Iodamoeba buetschlii*	119	556
Odds ratio	95% confidence interval	Significance level P
0.3665	0.1299, 1.034	0.0590, not significant

**Table A311:** Odds ratio for the association of *Hymenolepis* spp. and *Pentatrichomonas hominis*.

	*Hymenolepis* spp.	Non-*Hymenolepis* spp.
*Pentatrichomonas hominis*	1	2
Non-*Pentatrichomonas hominis*	122	604
Odds ratio	95% confidence interval	Significance level P
2.475	0.2226, 27.531	0.4261, not significant

**Table A312:** Odds ratio for the association of *Hymenolepis* spp. and *Encephalocytozoon* spp.

	*Hymenolepis* spp.	Non-*Hymenolepis* spp.
*Encephalocytozoon* spp.	2	5
Non-*Encephalocytozoon* spp.	43	237
Odds ratio	95% confidence interval	Significance level P
2.205	0.4141, 11.736	0.3021, not significant

**Table A313:** Odds ratio for the association of *Hymenolepis* spp. and *Aeromonas* spp.

	*Hymenolepis* spp.	Non-*Hymenolepis* spp.
*Aeromonas* spp.	0	5
Non-*Aeromonas* spp.	45	237
Odds ratio	95% confidence interval	Significance level P
0.4745	0.025577, 8.738	1.0000, not significant

**Table A314:** Odds ratio for the association of *Hymenolepis* spp. and conidia.

	*Hymenolepis* spp.	Non-*Hymenolepis* spp.
Conidia	10	31
Non-conidia	32	172
Odds ratio	95% confidence interval	Significance level P
1.734	0.7739, 3.885	0.1790, not significant

**Table A315:** Odds ratio for the association of *Hymenolepis* spp. and pseudoconidia.

	*Hymenolepis* spp.	Non-*Hymenolepis* spp.
Pseudoconidia	6	21
Non-pseudoconidia	36	182
Odds ratio	95% confidence interval	Significance level P
1.444	0.5446, 3.831	0.4262, not significant

**Table A316:** Odds ratio for the association of *Trichuris* spp. and *Schistosoma* spp.

	*Trichuris* spp.	Non-*Trichuris* spp.
*Schistosoma* spp.	1	3
Non-*Schistosoma* spp.	154	573
Odds ratio	95% confidence interval	Significance level P
1.240	0.1280, 12.014	1.0000, not significant

**Table A317:** Odds ratio for the association of *Trichuris* spp. and *Enterobius vermicularis*.

	*Trichuris* spp.	Non-*Trichuris* spp.
*Enterobius vermicularis*	14	17
Non-*Enterobius vermicularis*	144	598
Odds ratio	95% confidence interval	Significance level P
3.420	1.647, 7.101	0.0020, significant

**Table A318:** Odds ratio for the association of *Trichuris* spp. and *Tropheryma whipplei*.

	*Trichuris* spp.	Non-*Trichuris* spp.
*Tropheryma whipplei*	4	24
Non-*Tropheryma whipplei*	54	307
Odds ratio	95% confidence interval	Significance level P
0.9475	0.3162, 2.840	1.0000, not significant

**Table A319:** Odds ratio for the association of *Trichuris* spp. and enteropathogenic *Escherichia coli*.

	*Trichuris* spp.	Non-*Trichuris* spp.
Enteropathogenic *Escherichia coli*	51	159
Non-enteropathogenic *Escherichia coli*	101	254
Odds ratio	95% confidence interval	Significance level P
0.8067	0.5457, 1.192	0.3263, not significant

**Table A320:** Odds ratio for the association of *Trichuris* spp. and enterotoxigenic *Escherichia coli*.

	*Trichuris* spp.	Non-*Trichuris* spp.
Enterotoxigenic *Escherichia coli*	47	90
Non-enterotoxigenic *Escherichia coli*	105	323
Odds ratio	95% confidence interval	Significance level P
1.606	1.060, 2.435	0.0272, significant

**Table A321:** Odds ratio for the association of *Trichuris* spp. and enteroaggregative *Escherichia coli*.

	*Trichuris* spp.	Non-*Trichuris* spp.
Enteroaggregative *Escherichia coli*	55	122
Non-enteroaggregative *Escherichia coli*	97	291
Odds ratio	95% confidence interval	Significance level P
1.352	0.9132, 2.003	0.1521, not significant

**Table A322:** Odds ratio for the association of *Trichuris* spp. and Shiga toxin-producing *Escherichia coli*.

	*Trichuris* spp.	Non-*Trichuris* spp.
Shiga toxin-producing *Escherichia coli*	5	15
Non-Shiga toxin-producing *Escherichia coli*	37	71
Odds ratio	95% confidence interval	Significance level P
0.6396	0.2156, 1.898	0.6048, not significant

**Table A323:** Odds ratio for the association of *Trichuris* spp. and *Dientamoeba fragilis*.

	*Trichuris* spp.	Non-*Trichuris* spp.
*Dientamoeba fragilis*	39	36
Non-*Dientamoeba fragilis*	119	579
Odds ratio	95% confidence interval	Significance level P
5.271	3.216, 8.640	<0.0001, significant

**Table A324:** Odds ratio for the association of *Trichuris* spp. and *Blastocystis* spp.

	*Trichuris* spp.	Non-*Trichuris* spp.
*Blastocystis* spp.	97	294
Non-*Blastocystis* spp.	61	321
Odds ratio	95% confidence interval	Significance level P
1.736	1.215, 2.482	0.0024, significant

**Table A325:** Odds ratio for the association of *Trichuris* spp. and *Endolimax nana*.

	*Trichuris* spp.	Non-*Trichuris* spp.
*Endolimax nana*	51	165
Non-*Endolimax nana*	103	411
Odds ratio	95% confidence interval	Significance level P
1.233	0.8424, 1.806	0.2765, not significant

**Table A326:** Odds ratio for the association of *Trichuris* spp. and *Entamoeba coli*.

	*Trichuris* spp.	Non-*Trichuris* spp.
*Entamoeba coli*	44	183
Non-*Entamoeba coli*	110	392
Odds ratio	95% confidence interval	Significance level P
0.8568	0.5794, 1.267	0.4930, not significant

**Table A327:** Odds ratio for the association of *Trichuris* spp. and *Entamoeba dispar*.

	*Trichuris* spp.	Non-*Trichuris* spp.
*Entamoeba dispar*	39	79
Non-*Entamoeba dispar*	101	511
Odds ratio	95% confidence interval	Significance level P
2.498	1.610, 3.875	0.0001, significant

**Table A328:** Odds ratio for the association of *Trichuris* spp. and *Iodamoeba buetschlii*.

	*Trichuris* spp.	Non-*Trichuris* spp.
*Iodamoeba buetschlii*	8	47
Non-*Iodamoeba buetschlii*	146	529
Odds ratio	95% confidence interval	Significance level P
0.6167	0.2850, 1.334	0.3012, not significant

**Table A329:** Odds ratio for the association of *Trichuris* spp. and *Pentatrichomonas hominis*.

	*Trichuris* spp.	Non-*Trichuris* spp.
*Pentatrichomonas hominis*	0	3
Non-*Pentatrichomonas hominis*	154	572
Odds ratio	95% confidence interval	Significance level P
0.5294	0.02718, 10.310	1.0000, not significant

**Table A330:** Odds ratio for the association of *Trichuris* spp. and *Encephalocytozoon* spp.

	*Trichuris* spp.	Non-*Trichuris* spp.
*Encephalocytozoon* spp.	0	7
Non-*Encephalocytozoon* spp.	97	183
Odds ratio	95% confidence interval	Significance level P
0.1255	0.007086, 2.222	0.0996, not significant

**Table A331:** Odds ratio for the association of *Trichuris* spp. and *Aeromonas* spp.

	*Trichuris* spp.	Non-*Trichuris* spp.
*Aeromonas* spp.	3	2
Non-*Aeromonas* spp.	94	188
Odds ratio	95% confidence interval	Significance level P
3.000	0.4926, 18.271	0.3400, not significant

**Table A332:** Odds ratio for the association of *Trichuris* spp. and conidia.

	*Trichuris* spp.	Non-*Trichuris* spp.
Conidia	16	25
Non-conidia	78	126
Odds ratio	95% confidence interval	Significance level P
1.034	0.5194, 2.058	1.0000, not significant

**Table A333:** Odds ratio for the association of *Trichuris* spp. and pseudoconidia.

	*Trichuris* spp.	Non-*Trichuris* spp.
Pseudoconidia	11	16
Non-pseudoconidia	83	135
Odds ratio	95% confidence interval	Significance level P
1.118	0.4949, 2.527	0.8352, not significant

**Table A334:** Odds ratio for the association of *Schistosoma* spp. and *Enterobius vermicularis*.

	*Schistosoma* spp.	Non-*Schistosoma* spp.
*Enterobius vermicularis*	0	4
Non-*Enterobius vermicularis*	21	706
Odds ratio	95% confidence interval	Significance level P
3.651	0.1904, 70.025	1.0000, not significant

**Table A335:** Odds ratio for the association of *Schistosoma* spp. and *Tropheryma whipplei*.

	*Schistosoma* spp.	Non-*Schistosoma* spp.
*Tropheryma whipplei*	0	28
Non-*Tropheryma whipplei*	3	358
Odds ratio	95% confidence interval	Significance level P
1.797	0.09051, 35.676	1.0000, not significant

**Table A336:** Odds ratio for the association of *Schistosoma* spp. and enteropathogenic *Escherichia coli*.

	*Schistosoma* spp.	Non-*Schistosoma* spp.
Enteropathogenic *Escherichia coli*	3	207
Non-enteropathogenic *Escherichia coli*	1	354
Odds ratio	95% confidence interval	Significance level P
5.130	0.5299, 49.671	0.1473, not significant

**Table A337:** Odds ratio for the association of *Schistosoma* spp. and enterotoxigenic *Escherichia coli*.

	*Schistosoma* spp.	Non-*Schistosoma* spp.
Enterotoxigenic *Escherichia coli*	1	136
Non-enterotoxigenic *Escherichia coli*	3	425
Odds ratio	95% confidence interval	Significance level P
1.042	0.1074, 10.102	1.0000, not significant

**Table A338:** Odds ratio for the association of *Schistosoma* spp. and enteroaggregative *Escherichia coli*.

	*Schistosoma* spp.	Non-*Schistosoma* spp.
Enteroaggregative *Escherichia coli*	1	179
Non-enteroaggregative *Escherichia coli*	3	382
Odds ratio	95% confidence interval	Significance level P
0.7114	0.07344, 6.890	1.0000, not significant

**Table A339:** Odds ratio for the association of *Schistosoma* spp. and Shiga toxin-producing *Escherichia coli*.

	*Schistosoma* spp.	Non-*Schistosoma* spp.
Shiga toxin-producing *Escherichia coli*	0	20
Non-Shiga toxin-producing *Escherichia coli*	1	107
Odds ratio	95% confidence interval	Significance level P
1.748	0.06873, 44.457	1.0000, not significant

**Table A340:** Odds ratio for the association of *Schistosoma* spp. and *Dientamoeba fragilis*.

	*Schistosoma* spp.	Non-*Schistosoma* spp.
*Dientamoeba fragilis*	0	72
Non-*Dientamoeba fragilis*	4	655
Odds ratio	95% confidence interval	Significance level P
1.005	0.05351, 18.861	1.0000, not significant

**Table A341:** Odds ratio for the association of *Schistosoma* spp. and *Blastocystis* spp.

	*Schistosoma* spp.	Non-*Schistosoma* spp.
*Blastocystis* spp.	2	348
Non-*Blastocystis* spp.	2	379
Odds ratio	95% confidence interval	Significance level P
1.089	0.1525, 7.777	1.0000, not significant

**Table A342:** Odds ratio for the association of *Schistosoma* spp. and *Endolimax nana*.

	*Schistosoma* spp.	Non-*Schistosoma* spp.
*Endolimax nana*	0	216
Non-*Endolimax nana*	4	510
Odds ratio	95% confidence interval	Significance level P
0.2620	0.01403, 4.891	0.3250, not significant

**Table A343:** Odds ratio for the association of *Schistosoma* spp. and *Entamoeba coli*.

	*Schistosoma* spp.	Non-*Schistosoma* spp.
*Entamoeba coli*	0	227
Non-*Entamoeba coli*	4	498
Odds ratio	95% confidence interval	Significance level P
0.2435	0.01304, 4.544	0.3160, not significant

**Table A344:** Odds ratio for the association of *Schistosoma* spp. and *Entamoeba dispar*.

	*Schistosoma* spp.	Non-*Schistosoma* spp.
*Entamoeba dispar*	1	117
Non-*Entamoeba dispar*	3	609
Odds ratio	95% confidence interval	Significance level P
1.735	0.1788, 16.834	0.5068, not significant

**Table A345:** Odds ratio for the association of *Schistosoma* spp. and *Iodamoeba buetschlii*.

	*Schistosoma* spp.	Non-*Schistosoma* spp.
*Iodamoeba buetschlii*	1	54
Non-*Iodamoeba buetschlii*	3	672
Odds ratio	95% confidence interval	Significance level P
4.148	0.4240, 40.582	0.2695, not significant

**Table A346:** Odds ratio for the association of *Schistosoma* spp. and *Pentatrichomonas hominis*.

	*Schistosoma* spp.	Non-*Schistosoma* spp.
*Pentatrichomonas hominis*	0	3
Non-*Pentatrichomonas hominis*	4	722
Odds ratio	95% confidence interval	Significance level P
22.937	1.028, 511.96	1.0000, not significant

**Table A347:** Odds ratio for the association of *Schistosoma* spp. and *Encephalocytozoon* spp.

	*Schistosoma* spp.	Non-*Schistosoma* spp.
*Encephalocytozoon* spp.	0	7
Non-*Encephalocytozoon* spp.	1	237
Odds ratio	95% confidence interval	Significance level P
10.556	0.3960, 281.34	1.0000, not significant

**Table A348:** Odds ratio for the association of *Schistosoma* spp. and *Aeromonas* spp.

	*Schistosoma* spp.	Non-*Schistosoma* spp.
*Aeromonas* spp.	0	5
Non-*Aeromonas* spp.	1	239
Odds ratio	95% confidence interval	Significance level P
14.515	0.5294, 397.96	1.0000, not significant

**Table A349:** Odds ratio for the association of *Schistosoma* spp. and conidia.

	*Schistosoma* spp.	Non-*Schistosoma* spp.
Conidia	0	41
Non-conidia	1	203
Odds ratio	95% confidence interval	Significance level P
1.635	0.06539, 40.859	1.0000, not significant

**Table A350:** Odds ratio for the association of *Schistosoma* spp. and pseudoconidia.

	*Schistosoma* spp.	Non-*Schistosoma* spp.
Pseudoconidia	1	26
Non-pseudoconidia	0	218
Odds ratio	95% confidence interval	Significance level P
24.736	0.9817, 623.25	0.1102, not significant

**Table A351:** Odds ratio for the association of *Enterobius vermicularis* and *Tropheryma whipplei*.

	*Enterobius vermicularis*	Non-*Enterobius vermicularis*
*Tropheryma whipplei*	1	27
Non-*Tropheryma whipplei*	20	341
Odds ratio	95% confidence interval	Significance level P
0.6315	0.08156, 4.889	1.0000, not significant

**Table A352:** Odds ratio for the association of *Enterobius vermicularis* and enteropathogenic *Escherichia coli*.

	*Enterobius vermicularis*	Non-*Enterobius vermicularis*
Enteropathogenic *Escherichia coli*	15	195
Non-enteropathogenic *Escherichia coli*	15	340
Odds ratio	95% confidence interval	Significance level P
1.744	0.8342, 3.644	0.1733, not significant

**Table A353:** Odds ratio for the association of *Enterobius vermicularis* and enterotoxigenic *Escherichia coli*.

	*Enterobius vermicularis*	Non-*Enterobius vermicularis*
Enterotoxigenic *Escherichia coli*	9	128
Non-enterotoxigenic *Escherichia coli*	21	407
Odds ratio	95% confidence interval	Significance level P
1.363	0.6087, 3.051	0.5108, not significant

**Table A354:** Odds ratio for the association of *Enterobius vermicularis* and enteroaggregative *Escherichia coli*.

	*Enterobius vermicularis*	Non-*Enterobius vermicularis*
Enteroaggregative *Escherichia coli*	11	166
Non-enteroaggregative *Escherichia coli*	19	369
Odds ratio	95% confidence interval	Significance level P
1.287	0.5988, 2.766	0.5461, not significant

**Table A355:** Odds ratio for the association of *Enterobius vermicularis* and Shiga toxin-producing *Escherichia coli*.

	*Enterobius vermicularis*	Non-*Enterobius vermicularis*
Shiga toxin-producing *Escherichia coli*	2	18
Non-Shiga toxin-producing *Escherichia coli*	3	105
Odds ratio	95% confidence interval	Significance level P
3.889	0.6065, 24.936	0.1735, not significant

**Table A356:** Odds ratio for the association of *Enterobius vermicularis* and *Dientamoeba fragilis*.

	*Enterobius vermicularis*	Non-*Enterobius vermicularis*
*Dientamoeba fragilis*	5	70
Non-*Dientamoeba fragilis*	26	672
Odds ratio	95% confidence interval	Significance level P
1.846	0.6870, 4.961	0.2128, not significant

**Table A357:** Odds ratio for the association of *Enterobius vermicularis* and *Blastocystis* spp.

	*Enterobius vermicularis*	Non-*Enterobius vermicularis*
*Blastocystis* spp.	15	376
Non-*Blastocystis* spp.	16	366
Odds ratio	95% confidence interval	Significance level P
0.9126	0.4446, 1.873	0.8558, not significant

**Table A358:** Odds ratio for the association of *Enterobius vermicularis* and *Endolimax nana*.

	*Enterobius vermicularis*	Non-*Enterobius vermicularis*
*Endolimax nana*	9	230
Non-*Endolimax nana*	22	469
Odds ratio	95% confidence interval	Significance level P
0.8342	0.3780, 1.841	0.8452, not significant

**Table A359:** Odds ratio for the association of *Enterobius vermicularis* and *Entamoeba coli*.

	*Enterobius vermicularis*	Non-*Enterobius vermicularis*
*Entamoeba coli*	10	217
Non-*Entamoeba coli*	21	481
Odds ratio	95% confidence interval	Significance level P
1.056	0.4887, 2.280	0.8458, not significant

**Table A360:** Odds ratio for the association of *Enterobius vermicularis* and *Entamoeba dispar*.

	*Enterobius vermicularis*	Non-*Enterobius vermicularis*
*Entamoeba dispar*	5	113
Non-*Entamoeba dispar*	26	586
Odds ratio	95% confidence interval	Significance level P
0.9973	0.3749, 2.653	1.0000, not significant

**Table A361:** Odds ratio for the association of *Enterobius vermicularis* and *Iodamoeba buetschlii*.

	*Enterobius vermicularis*	Non-*Enterobius vermicularis*
*Iodamoeba buetschlii*	3	52
Non-*Iodamoeba buetschlii*	28	647
Odds ratio	95% confidence interval	Significance level P
1.333	0.3920, 4.534	0.5019, not significant

**Table A362:** Odds ratio for the association of *Enterobius vermicularis* and *Pentatrichomonas hominis*.

	*Enterobius vermicularis*	Non-*Enterobius vermicularis*
*Pentatrichomonas hominis*	0	3
Non-*Pentatrichomonas hominis*	31	695
Odds ratio	95% confidence interval	Significance level P
3.154	0.1594, 62.433	1.0000, not significant

**Table A363:** Odds ratio for the association of *Enterobius vermicularis* and *Encephalocytozoon* spp.

	*Enterobius vermicularis*	Non-*Enterobius vermicularis*
*Encephalocytozoon* spp.	0	7
Non-*Encephalocytozoon* spp.	9	271
Odds ratio	95% confidence interval	Significance level P
1.905	0.1012, 35.882	1.0000, not significant

**Table A364:** Odds ratio for the association of *Enterobius vermicularis* and *Aeromonas* spp.

	*Enterobius vermicularis*	Non-*Enterobius vermicularis*
*Aeromonas* spp.	0	5
Non-*Aeromonas* spp.	9	273
Odds ratio	95% confidence interval	Significance level P
2.617	0.1347, 50.871	1.0000, not significant

**Table A365:** Odds ratio for the association of *Enterobius vermicularis* and conidia.

	*Enterobius vermicularis*	Non-*Enterobius vermicularis*
Conidia	4	37
Non-conidia.	5	199
Odds ratio	95% confidence interval	Significance level P
4.303	1.103, 16.783	0.0454, significant

**Table A366:** Odds ratio for the association of *Enterobius vermicularis* and pseudoconidia.

	*Enterobius vermicularis*	Non-*Enterobius vermicularis*
Pseudoconidia	1	26
Non-pseudoconidia.	8	210
Odds ratio	95% confidence interval	Significance level P
1.010	0.1213, 8.402	1.0000, not significant

**Table A367:** Odds ratio for the association of *Tropheryma whipplei* and enteropathogenic *Escherichia coli*.

	*Tropheryma whipplei*	Non-*Tropheryma whipplei*
Enteropathogenic *Escherichia coli*	14	140
Non-enteropathogenic *Escherichia coli*	14	221
Odds ratio	95% confidence interval	Significance level P
1.579	0.7304, 3.412	0.3158, not significant

**Table A368:** Odds ratio for the association of *Tropheryma whipplei* and enterotoxigenic *Escherichia coli*.

	*Tropheryma whipplei*	Non-*Tropheryma whipplei*
Enterotoxigenic *Escherichia coli*	4	80
Non-enterotoxigenic *Escherichia coli*	24	281
Odds ratio	95% confidence interval	Significance level P
0.5854	0.1973, 1.737	0.4745, not significant

**Table A369:** Odds ratio for the association of *Tropheryma whipplei* and enteroaggregative *Escherichia coli*.

	*Tropheryma whipplei*	Non-*Tropheryma whipplei*
Enteroaggregative *Escherichia coli*	8	130
Non-enteroaggregative *Escherichia coli*	20	231
Odds ratio	95% confidence interval	Significance level P
0.7108	0.3045, 1.659	0.5400, not significant

**Table A370:** Odds ratio for the association of *Tropheryma whipplei* and Shiga toxin-producing *Escherichia coli*.

	*Tropheryma whipplei*	Non-*Tropheryma whipplei*
Shiga toxin-producing *Escherichia coli*	1	19
Non-Shiga toxin-producing *Escherichia coli*	9	99
Odds ratio	95% confidence interval	Significance level P
0.5789	0.06921, 4.843	1.0000, not significant

**Table A371:** Odds ratio for the association of *Tropheryma whipplei* and *Blastocystis* spp.

	*Tropheryma whipplei*	Non-*Tropheryma whipplei*
*Blastocystis* spp.	3	100
Non-*Blastocystis* spp.	25	261
Odds ratio	95% confidence interval	Significance level P
0.3132	0.09248, 1.061	0.0724, not significant

**Table A372:** Odds ratio for the association of *Tropheryma whipplei* and *Endolimax nana*.

	*Tropheryma whipplei*	Non-*Tropheryma whipplei*
*Endolimax nana*	2	72
Non-*Endolimax nana*	26	289
Odds ratio	95% confidence interval	Significance level P
0.3088	0.07159, 1.332	0.1320, not significant

**Table A373:** Odds ratio for the association of *Tropheryma whipplei* and *Entamoeba coli*.

	*Tropheryma whipplei*	Non-*Tropheryma whipplei*
*Entamoeba coli*	8	83
Non-*Entamoeba coli*	20	278
Odds ratio	95% confidence interval	Significance level P
1.340	0.5692, 3.153	0.4918, not significant

**Table A374:** Odds ratio for the association of *Tropheryma whipplei* and *Entamoeba dispar*.

	*Tropheryma whipplei*	Non-*Tropheryma whipplei*
*Entamoeba dispar*	1	22
Non-*Entamoeba dispar*	27	339
Odds ratio	95% confidence interval	Significance level P
0.5707	0.07402, 4.400	1.0000, not significant

**Table A375:** Odds ratio for the association of *Tropheryma whipplei* and *Iodamoeba buetschlii*.

	*Tropheryma whipplei*	Non-*Tropheryma whipplei*
*Iodamoeba buetschlii*	3	27
Non-*Iodamoeba buetschlii*	25	334
Odds ratio	95% confidence interval	Significance level P
1.484	0.4209, 5.236	0.4658, not significant

**Table A376:** Odds ratio for the association of *Tropheryma whipplei* and *Pentatrichomonas hominis*.

	*Tropheryma whipplei*	Non-*Tropheryma whipplei*
*Pentatrichomonas hominis*	0	1
Non-*Pentatrichomonas hominis*	28	360
Odds ratio	95% confidence interval	Significance level P
4.216	0.1678, 105.96	1.0000, not significant

**Table A377:** Odds ratio for the association of enteropathogenic *Escherichia coli* and enterotoxigenic *Escherichia coli*.

	Enteropathogenic *Escherichia coli*	Non-Enteropathogenic *Escherichia coli*
Enterotoxigenic *Escherichia coli*	85	52
Non-enterotoxigenic *Escherichia coli*	125	303
Odds ratio	95% confidence interval	Significance level P
3.962	2.648, 5.930	<0.0001, significant

**Table A378:** Odds ratio for the association of enteropathogenic *Escherichia coli* and enteroaggregative *Escherichia coli*.

	Enteropathogenic *Escherichia coli*	Non-Enteropathogenic *Escherichia coli*
Enteroaggregative *Escherichia coli*	94	83
Non-enteroaggregative *Escherichia coli*	116	272
Odds ratio	95% confidence interval	Significance level P
2.656	1.840, 3.832	<0.0001, significant

**Table A379:** Odds ratio for the association of enteropathogenic *Escherichia coli* and Shiga toxin-producing *Escherichia coli*.

	Enteropathogenic *Escherichia coli*	Non-Enteropathogenic *Escherichia coli*
Shiga toxin-producing *Escherichia coli*	13	7
Non-Shiga toxin-producing *Escherichia coli*	41	67
Odds ratio	95% confidence interval	Significance level P
3.035	1.119, 8.232	0.0288, significant

**Table A380:** Odds ratio for the association of enteropathogenic *Escherichia coli* and *Dientamoeba fragilis*.

	Enteropathogenic *Escherichia coli*	Non-Enteropathogenic *Escherichia coli*
*Dientamoeba fragilis*	22	43
Non-*Dientamoeba fragilis*	188	312
Odds ratio	95% confidence interval	Significance level P
8.868	4.404, 17.857	<0.0001, significant

**Table A381:** Odds ratio for the association of enteropathogenic *Escherichia coli* and *Blastocystis* spp.

	Enteropathogenic *Escherichia coli*	Non-Enteropathogenic *Escherichia coli*
*Blastocystis* spp.	86	174
Non-*Blastocystis* spp.	124	181
Odds ratio	95% confidence interval	Significance level P
0.7214	0.5110, 1.019	0.0671, not significant

**Table A382:** Odds ratio for the association of enteropathogenic *Escherichia coli* and *Endolimax nana*.

	Enteropathogenic *Escherichia coli*	Non-Enteropathogenic *Escherichia coli*
*Endolimax nana*	60	101
Non-*Endolimax nana*	149	254
Odds ratio	95% confidence interval	Significance level P
1.013	0.6938, 1.478	1.0000, not significant

**Table A383:** Odds ratio for the association of enteropathogenic *Escherichia coli* and *Entamoeba coli*.

	Enteropathogenic *Escherichia coli*	Non-Enteropathogenic *Escherichia coli*
*Entamoeba coli*	60	111
Non-*Entamoeba coli*	149	243
Odds ratio	95% confidence interval	Significance level P
0.8816	0.6062, 1.282	0.5694, not significant

**Table A384:** Odds ratio for the association of enteropathogenic *Escherichia coli* and *Entamoeba dispar*.

	Enteropathogenic *Escherichia coli*	Non-Enteropathogenic *Escherichia coli*
*Entamoeba dispar*	30	58
Non-*Entamoeba dispar*	179	297
Odds ratio	95% confidence interval	Significance level P
0.8582	0.5319, 1.385	0.5507, not significant

**Table A385:** Odds ratio for the association of enteropathogenic *Escherichia coli* and *Iodamoeba buetschlii*.

	Enteropathogenic *Escherichia coli*	Non-Enteropathogenic *Escherichia coli*
*Iodamoeba buetschlii*	17	28
Non-*Iodamoeba buetschlii*	192	327
Odds ratio	95% confidence interval	Significance level P
1.034	0.5515, 1.939	1.0000, not significant

**Table A386:** Odds ratio for the association of enteropathogenic *Escherichia coli* and *Pentatrichomonas hominis*.

	Enteropathogenic *Escherichia coli*	Non-Enteropathogenic *Escherichia coli*
*Pentatrichomonas hominis*	1	1
Non-*Pentatrichomonas hominis*.	208	353
Odds ratio	95% confidence interval	Significance level P
1.697	0.1055, 27.295	1.0000, not significant

**Table A387:** Odds ratio for the association of enteropathogenic *Escherichia coli* and *Encephalocytozoon* spp.

	Enteropathogenic *Escherichia coli*	Non-Enteropathogenic *Escherichia coli*
*Encephalocytozoon* spp.	1	0
Non-*Encephalocytozoon* spp.	54	120
Odds ratio	95% confidence interval	Significance level P
6.633	0.2657, 165.57	0.3143, not significant

**Table A388:** Odds ratio for the association of enteropathogenic *Escherichia coli* and *Aeromonas* spp.

	Enteropathogenic *Escherichia coli*	Non-Enteropathogenic *Escherichia coli*
*Aeromonas* spp.	2	2
Non-*Aeromonas* spp.	53	118
Odds ratio	95% confidence interval	Significance level P
2.226	0.3052, 16.240	0.5909, not significant

**Table A389:** Odds ratio for the association of enteropathogenic *Escherichia coli* and conidia.

	Enteropathogenic *Escherichia coli*	Non-Enteropathogenic *Escherichia coli*
Conidia	12	19
Non-conidia.	43	101
Odds ratio	95% confidence interval	Significance level P
1.483	0.6624, 3.322	0.3945, not significant

**Table A390:** Odds ratio for the association of enteropathogenic *Escherichia coli* and pseudoconidia.

	Enteropathogenic *Escherichia coli*	Non-Enteropathogenic *Escherichia coli*
Pseudoconidia	8	15
Non-pseudoconidia.	47	105
Odds ratio	95% confidence interval	Significance level P
1.191	0.4726, 3.004	0.8101, not significant

**Table A391:** Odds ratio for the association of enterotoxigenic *Escherichia coli* and enteroaggregative *Escherichia coli*.

	Enterotoxigenic *Escherichia coli*	Non-enterotoxigenic *Escherichia coli*
Enteroaggregative *Escherichia coli*	78	99
Non-enteroaggregative *Escherichia coli*	59	329
Odds ratio	95% confidence interval	Significance level P
4.393	2.927, 6.594	<0.0001, significant

**Table A392:** Odds ratio for the association of enterotoxigenic *Escherichia coli* and Shiga toxin-producing *Escherichia coli*.

	Enterotoxigenic *Escherichia coli*	Non-enterotoxigenic *Escherichia coli*
Shiga toxin-producing *Escherichia coli*	12	8
Non-Shiga toxin-producing *Escherichia coli*	40	68
Odds ratio	95% confidence interval	Significance level P
2.550	0.9605, 6.770	0.0813, not significant

**Table A393:** Odds ratio for the association of enterotoxigenic *Escherichia coli* and *Dientamoeba fragilis*.

	Enterotoxigenic *Escherichia coli*	Non-enterotoxigenic *Escherichia coli*
*Dientamoeba fragilis*	18	47
Non-*Dientamoeba fragilis*	119	381
Odds ratio	95% confidence interval	Significance level P
1.226	0.6858, 2.192	0.5383, not significant

**Table A394:** Odds ratio for the association of enterotoxigenic *Escherichia coli* and *Blastocystis* spp.

	Enterotoxigenic *Escherichia coli*	Non-enterotoxigenic *Escherichia coli*
*Blastocystis* spp.	61	199
Non-*Blastocystis* spp.	76	229
Odds ratio	95% confidence interval	Significance level P
0.9236	0.6273, 1.360	0.6950, not significant

**Table A395:** Odds ratio for the association of enterotoxigenic *Escherichia coli* and *Endolimax nana*.

	Enterotoxigenic *Escherichia coli*	Non-enterotoxigenic *Escherichia coli*
*Endolimax nana*	33	128
Non-*Endolimax nana*	104	299
Odds ratio	95% confidence interval	Significance level P
0.7412	0.4759, 1.154	0.1939, not significant

**Table A396:** Odds ratio for the association of enterotoxigenic *Escherichia coli* and *Entamoeba coli*.

	Enterotoxigenic *Escherichia coli*	Non-enterotoxigenic *Escherichia coli*
*Entamoeba coli*	35	136
Non-*Entamoeba coli*	102	290
Odds ratio	95% confidence interval	Significance level P
0.7317	0.4737, 1.130	0.1665, not significant

**Table A397:** Odds ratio for the association of enterotoxigenic *Escherichia coli* and *Entamoeba dispar*.

	Enterotoxigenic *Escherichia coli*	Non-enterotoxigenic *Escherichia coli*
*Entamoeba dispar*	15	73
Non-*Entamoeba dispar*	122	354
Odds ratio	95% confidence interval	Significance level P
0.5962	0.3296, 1.078	0.1039, not significant

**Table A398:** Odds ratio for the association of enterotoxigenic *Escherichia coli* and *Iodamoeba buetschlii*.

	Enterotoxigenic *Escherichia coli*	Non-enterotoxigenic *Escherichia coli*
*Iodamoeba buetschlii*	7	38
Non-*Iodamoeba buetschlii*	130	389
Odds ratio	95% confidence interval	Significance level P
0.5512	0.2403, 1.265	0.2039, not significant

**Table A399:** Odds ratio for the association of enterotoxigenic *Escherichia coli* and *Pentatrichomonas hominis*.

	Enterotoxigenic *Escherichia coli*	Non-enterotoxigenic *Escherichia coli*
*Pentatrichomonas hominis*	1	1
Non-*Pentatrichomonas hominis*	135	426
Odds ratio	95% confidence interval	Significance level P
3.156	0.1959, 50.826	0.4251, not significant

**Table A400:** Odds ratio for the association of enterotoxigenic *Escherichia coli* and *Encephalocytozoon* spp.

	Enterotoxigenic *Escherichia coli*	Non-enterotoxigenic *Escherichia coli*
*Encephalocytozoon* spp.	1	0
Non-*Encephalocytozoon* spp.	52	122
Odds ratio	95% confidence interval	Significance level P
7.000	0.2803, 174.79	0.3029, not significant

**Table A401:** Odds ratio for the association of enterotoxigenic *Escherichia coli* and *Aeromonas* spp.

	Enterotoxigenic *Escherichia coli*	Non-enterotoxigenic *Escherichia coli*
*Aeromonas* spp.	3	1
Non-*Aeromonas* spp.	50	121
Odds ratio	95% confidence interval	Significance level P
7.260	0.7370, 71.520	0.0835, not significant

**Table A402:** Odds ratio for the association of enterotoxigenic *Escherichia coli* and conidia.

	Enterotoxigenic *Escherichia coli*	Non-enterotoxigenic *Escherichia coli*
Conidia	8	23
Non-conidia	45	99
Odds ratio	95% confidence interval	Significance level P
0.7652	0.3179, 1.842	0.6684, not significant

**Table A403:** Odds ratio for the association of enterotoxigenic *Escherichia coli* and pseudoconidia.

	Enterotoxigenic *Escherichia coli*	Non-enterotoxigenic *Escherichia coli*
Pseudoconidia	9	14
Non-pseudoconidia	44	108
Odds ratio	95% confidence interval	Significance level P
1.578	0.6364, 3.912	0.3369, not significant

**Table A404:** Odds ratio for the association of enteroaggregative *Escherichia coli* and Shiga toxin-producing *Escherichia coli*.

	Enteroaggregative *Escherichia coli*	Non-enteroaggregative *Escherichia coli*
Shiga toxin-producing *Escherichia coli*	17	3
Non-Shiga toxin-producing *Escherichia coli*	69	39
Odds ratio	95% confidence interval	Significance level P
3.203	0.8826, 11.623	0.0740, not significant

**Table A405:** Odds ratio for the association of enteroaggregative *Escherichia coli* and *Dientamoeba fragilis*.

	Enteroaggregative *Escherichia coli*	Non-enteroaggregative *Escherichia coli*
*Dientamoeba fragilis*	13	52
Non-*Dientamoeba fragilis*	164	336
Odds ratio	95% confidence interval	Significance level P
0.5122	0.2712, 0.9674	0.0457, significant

**Table A406:** Odds ratio for the association of enteroaggregative *Escherichia coli* and *Blastocystis* spp.

	Enteroaggregative *Escherichia coli*	Non-enteroaggregative *Escherichia coli*
*Blastocystis* spp.	52	208
Non-*Blastocystis* spp.	125	180
Odds ratio	95% confidence interval	Significance level P
0.3600	0.2462, 0.5265	<0.0001, significant

**Table A407:** Odds ratio for the association of enteroaggregative *Escherichia coli* and *Endolimax nana*.

	Enteroaggregative *Escherichia coli*	Non-enteroaggregative *Escherichia coli*
*Endolimax nana*	31	130
Non-*Endolimax nana*	145	258
Odds ratio	95% confidence interval	Significance level P
0.4243	0.2728, 0.6598	<0.0001, significant

**Table A408:** Odds ratio for the association of enteroaggregative *Escherichia coli* and *Entamoeba coli*.

	Enteroaggregative *Escherichia coli*	Non-enteroaggregative *Escherichia coli*
*Entamoeba coli*	31	140
Non-*Entamoeba coli*	145	247
Odds ratio	95% confidence interval	Significance level P
0.3772	0.2430, 0.5856	<0.0001, significant

**Table A409:** Odds ratio for the association of enteroaggregative *Escherichia coli* and *Entamoeba dispar*.

	Enteroaggregative *Escherichia coli*	Non-enteroaggregative *Escherichia coli*
*Entamoeba dispar*	16	72
Non-*Entamoeba dispar*	160	316
Odds ratio	95% confidence interval	Significance level P
0.4389	0.2471, 0.7794	0.0038, significant

**Table A410:** Odds ratio for the association of enteroaggregative *Escherichia coli* and *Iodamoeba buetschlii*.

	Enteroaggregative *Escherichia coli*	Non-enteroaggregative *Escherichia coli*
*Iodamoeba buetschlii*	6	39
Non-*Iodamoeba buetschlii*	170	349
Odds ratio	95% confidence interval	Significance level P
0.3158	0.1311, 0.7607	0.0067, significant

**Table A411:** Odds ratio for the association of enteroaggregative *Escherichia coli* and *Pentatrichomonas hominis*.

	Enteroaggregative *Escherichia coli*	Non-enteroaggregative *Escherichia coli*
*Pentatrichomonas hominis*	0	2
Non-*Pentatrichomonas hominis*	176	385
Odds ratio	95% confidence interval	Significance level P
0.4368	0.02085, 9.153	1.0000, not significant

**Table A412:** Odds ratio for the association of enteroaggregative *Escherichia coli* and *Encephalocytozoon* spp.

	Enteroaggregative *Escherichia coli*	Non-enteroaggregative *Escherichia coli*
*Encephalocytozoon* spp.	1	0
Non-*Encephalocytozoon* spp.	37	137
Odds ratio	95% confidence interval	Significance level P
11.000	0.4388, 275.78	0.2171, not significant

**Table A413:** Odds ratio for the association of enteroaggregative *Escherichia coli* and *Aeromonas* spp.

	Enteroaggregative *Escherichia coli*	Non-enteroaggregative *Escherichia coli*
*Aeromonas* spp.	2	2
Non-*Aeromonas* spp.	37	134
Odds ratio	95% confidence interval	Significance level P
3.622	0.4931, 26.599	0.2153, not significant

**Table A414:** Odds ratio for the association of enteroaggregative *Escherichia coli* and conidia.

	Enteroaggregative *Escherichia coli*	Non-enteroaggregative *Escherichia coli*
Conidia	8	23
Non-conidia	30	114
Odds ratio	95% confidence interval	Significance level P
1.322	0.5376, 3.250	0.6311, not significant

**Table A415:** Odds ratio for the association of enteroaggregative *Escherichia coli* and pseudoconidia.

	Enteroaggregative *Escherichia coli*	Non-enteroaggregative *Escherichia coli*
Pseudoconidia	6	17
Non-pseudoconidia	32	120
Odds ratio	95% confidence interval	Significance level P
1.324	0.4824, 3.631	0.5917, not significant

**Table A416:** Odds ratio for the association of *Dientamoeba fragilis* and *Blastocystis* spp.

	*Dientamoeba fragilis*	Non-*Dientamoeba fragilis*
*Blastocystis* spp.	70	5
Non-*Blastocystis* spp.	321	377
Odds ratio	95% confidence interval	Significance level P
16.442	6.555, 41.244	<0.0001, significant

**Table A417:** Odds ratio for the association of *Dientamoeba fragilis* and *Endolimax nana*.

	*Dientamoeba fragilis*	Non-*Dientamoeba fragilis*
*Endolimax nana*	36	180
Non-*Endolimax nana*	36	478
Odds ratio	95% confidence interval	Significance level P
2.656	1.622, 4.347	0.0001, significant

**Table A418:** Odds ratio for the association of *Dientamoeba fragilis* and *Entamoeba coli*.

	*Dientamoeba fragilis*	Non-*Dientamoeba fragilis*
*Entamoeba coli*	42	185
Non-*Entamoeba coli*	30	445
Odds ratio	95% confidence interval	Significance level P
3.368	2.045, 5.547	<0.0001, significant

**Table A419:** Odds ratio for the association of *Dientamoeba fragilis* and *Entamoeba dispar*.

	*Dientamoeba fragilis*	Non-*Dientamoeba fragilis*
*Entamoeba dispar*	30	88
Non-*Entamoeba dispar*	42	570
Odds ratio	95% confidence interval	Significance level P
4.627	2.752, 7.779	<0.0001, significant

**Table A420:** Odds ratio for the association of *Dientamoeba fragilis* and *Iodamoeba buetschlii*.

	*Dientamoeba fragilis*	Non-*Dientamoeba fragilis*
*Iodamoeba buetschlii*	5	50
Non-*Iodamoeba buetschlii*	67	608
Odds ratio	95% confidence interval	Significance level P
0.9075	0.3497, 2.355	1.0000, not significant

**Table A421:** Odds ratio for the association of *Dientamoeba fragilis* and *Pentatrichomonas hominis*.

	*Dientamoeba fragilis*	Non-*Dientamoeba fragilis*
*Pentatrichomonas hominis*	0	3
Non-*Pentatrichomonas hominis*	71	655
Odds ratio	95% confidence interval	Significance level P
1.310	0.06693, 25.630	1.0000, not significant

**Table A422:** Odds ratio for the association of *Dientamoeba fragilis* and *Encephalocytozoon* spp.

	*Dientamoeba fragilis*	Non-*Dientamoeba fragilis*
*Encephalocytozoon* spp.	0	7
Non-*Encephalocytozoon* spp.	75	205
Odds ratio	95% confidence interval	Significance level P
0.1815	0.1023, 3.218	0.1958, not significant

**Table A423:** Odds ratio for the association of *Dientamoeba fragilis* and *Aeromonas* spp.

	*Dientamoeba fragilis*	Non-*Dientamoeba fragilis*
*Aeromonas* spp.	1	4
Non-*Aeromonas* spp.	74	208
Odds ratio	95% confidence interval	Significance level P
0.7027	0.07725, 6.392	1.0000, not significant

**Table A424:** Odds ratio for the association of *Dientamoeba fragilis* and conidia.

	*Dientamoeba fragilis*	Non-*Dientamoeba fragilis*
Conidia	9	32
Non-conidia	63	141
Odds ratio	95% confidence interval	Significance level P
0.6295	0.2837, 1.397	0.3473, not significant

**Table A425:** Odds ratio for the association of *Dientamoeba fragilis* and pseudoconidia.

	*Dientamoeba fragilis*	Non-*Dientamoeba fragilis*
*Shigella* spp.	6	21
Non-*Shigella* spp.	66	152
Odds ratio	95% confidence interval	Significance level P
0.6580	0.2539, 1.706	0.5034, not significant

**Table A426:** Odds ratio for the association of *Blastocystis* spp. and *Endolimax nana*.

	*Blastocystis* spp.	Non-*Blastocystis* spp.
*Endolimax nana*	149	67
Non-*Endolimax nana*	201	313
Odds ratio	95% confidence interval	Significance level P
3.463	2.469, 4.858	<0.0001, significant

**Table A427:** Odds ratio for the association of *Blastocystis* spp. and *Entamoeba coli*.

	*Blastocystis* spp.	Non-*Blastocystis* spp.
*Entamoeba coli*	158	69
Non-*Entamoeba coli*	192	310
Odds ratio	95% confidence interval	Significance level P
3.697	2.644, 5.170	<0.0001, significant

**Table A428:** Odds ratio for the association of *Blastocystis* spp. and *Entamoeba dispar*.

	*Blastocystis* spp.	Non-*Blastocystis* spp.
*Entamoeba dispar*	96	22
Non-*Entamoeba dispar*	254	358
Odds ratio	95% confidence interval	Significance level P
6.150	3.766, 10.044	<0.0001, significant

**Table A429:** Odds ratio for the association of *Blastocystis* spp. and *Iodamoeba buetschlii*.

	*Blastocystis* spp.	Non-*Blastocystis* spp.
*Iodamoeba buetschlii*	41	14
Non-*Iodamoeba buetschlii*	309	366
Odds ratio	95% confidence interval	Significance level P
3.469	1.856, 6.484	<0.0001, significant

**Table A430:** Odds ratio for the association of *Blastocystis* spp. and *Pentatrichomonas hominis*.

	*Blastocystis* spp.	Non-*Blastocystis* spp.
*Pentatrichomonas hominis*	2	1
Non-*Pentatrichomonas hominis*	347	379
Odds ratio	95% confidence interval	Significance level P
2.184	0.1971, 24.211	0.6092, not significant

**Table A431:** Odds ratio for the association of *Blastocystis* spp. and *Encephalocytozoon* spp.

	*Blastocystis* spp.	Non-*Blastocystis* spp.
*Encephalocytozoon* spp.	7	0
Non-*Encephalocytozoon* spp.	258	22
Odds ratio	95% confidence interval	Significance level P
1.306	0.7215, 23.626	1.0000, not significant

**Table A432:** Odds ratio for the association of *Blastocystis* spp. and *Aeromonas* spp.

	*Blastocystis* spp.	Non-*Blastocystis* spp.
*Aeromonas* spp..	4	1
Non-*Aeromonas* spp.	261	21
Odds ratio	95% confidence interval	Significance level P
0.3218	0.03438, 3.013	0.3308, not significant

**Table A433:** Odds ratio for the association of *Blastocystis* spp. and conidia.

	*Blastocystis* spp.	Non-*Blastocystis* spp.
Conidia	34	7
Non-conidia	194	10
Odds ratio	95% confidence interval	Significance level P
0.2504	0.8915, 0.7031	0.0118, significant

**Table A434:** Odds ratio for the association of *Blastocystis* spp. and pseudoconidia.

	*Blastocystis* spp.	Non-*Blastocystis* spp.
Pseudoconidia	24	3
Non-pseudoconidia	205	13
Odds ratio	95% confidence interval	Significance level P
0.5073	0.1348, 1.909	0.3969, not significant

**Table A435:** Odds ratio for the association of *Endolimax nana* and *Entamoeba coli*.

	*Endolimax nana*	Non-*Endolimax nana*
*Entamoeba coli*	113	114
Non-*Entamoeba coli*	103	399
Odds ratio	95% confidence interval	Significance level P
3.840	2.737, 5.387	<0.0001, significant

**Table A436:** Odds ratio for the association of *Endolimax nana* and *Entamoeba dispar*.

	*Endolimax nana*	Non-*Endolimax nana*
*Entamoeba dispar*	61	57
Non-*Entamoeba dispar*	155	457
Odds ratio	95% confidence interval	Significance level P
3.155	2.106, 4.728	<0.0001, significant

**Table A437:** Odds ratio for the association of *Endolimax nana* and *Iodamoeba buetschlii*.

	*Endolimax nana*	Non-*Endolimax nana*
*Iodamoeba buetschlii*	38	17
Non-*Iodamoeba buetschlii*	178	497
Odds ratio	95% confidence interval	Significance level P
6.241	3.435, 11.340	<0.0001, significant

**Table A438:** Odds ratio for the association of *Endolimax nana* and *Pentatrichomonas hominis*.

	*Endolimax nana*	Non-*Endolimax nana*
*Pentatrichomonas hominis*	1	2
Non-*Pentatrichomonas hominis*	215	511
Odds ratio	95% confidence interval	Significance level P
1.188	0.1071, 13.183	1.0000, not significant

**Table A439:** Odds ratio for the association of *Endolimax nana* and *Encephalocytozoon* spp.

	*Endolimax nana*	Non-*Endolimax nana*
*Encephalocytozoon* spp.	5	2
Non-*Encephalocytozoon* spp.	115	123
Odds ratio	95% confidence interval	Significance level P
2.674	0.5085, 14.060	0.2733, not significant

**Table A440:** Odds ratio for the association of *Endolimax nana* and *Aeromonas* spp.

	*Endolimax nana*	Non-*Endolimax nana*
*Aeromonas* spp.	3	2
Non-*Aeromonas* spp.	117	123
Odds ratio	95% confidence interval	Significance level P
1.577	0.2587, 9.611	0.6787, not significant

**Table A441:** Odds ratio for the association of *Endolimax nana* and conidia.

	*Endolimax nana*	Non-*Endolimax nana*
Conidia	18	23
Non-conidia	102	102
Odds ratio	95% confidence interval	Significance level P
0.7826	0.3984, 1.537	0.4984, not significant

**Table A442:** Odds ratio for the association of *Endolimax nana* and pseudoconidia.

	*Endolimax nana*	Non-*Endolimax nana*
Pseudoconidia	13	14
Non-pseudoconidia	107	111
Odds ratio	95% confidence interval	Significance level P
0.9633	0.4326, 2.145	1.0000, not significant

**Table A443:** Odds ratio for the association of *Entamoeba coli* and *Entamoeba dispar*.

	*Entamoeba coli*	Non-*Entamoeba coli*
*Entamoeba dispar*.	69	49
Non-*Entamoeba dispar*	158	453
Odds ratio	95% confidence interval	Significance level P
4.037	2.683, 6.075	<0.0001, significant

**Table A444:** Odds ratio for the association of *Entamoeba coli* and *Iodamoeba buetschlii*.

	*Entamoeba coli*	Non-*Entamoeba coli*
*Iodamoeba buetschlii*	36	19
Non-*Iodamoeba buetschlii*	191	483
Odds ratio	95% confidence interval	Significance level P
4.791	2.681, 8.563	<0.0001, significant

**Table A445:** Odds ratio for the association of *Entamoeba coli* and *Pentatrichomonas hominis*.

	*Entamoeba coli*	Non-*Entamoeba coli*
*Pentatrichomonas hominis*	1	2
Non-*Pentatrichomonas hominis*	226	499
Odds ratio	95% confidence interval	Significance level P
1.104	0.09953, 12.245	1.0000, not significant

**Table A446:** Odds ratio for the association of *Entamoeba coli* and *Encephalocytozoon* spp.

	*Entamoeba coli*	Non-*Entamoeba coli*
*Encephalocytozoon* spp.	2	5
Non-*Encephalocytozoon* spp.	115	122
Odds ratio	95% confidence interval	Significance level P
0.4243	0.08069, 2.232	0.4491, not significant

**Table A447:** Odds ratio for the association of *Entamoeba coli* and *Aeromonas* spp.

	*Entamoeba coli*	Non-*Entamoeba coli*
*Aeromonas* spp.	4	1
Non-*Aeromonas* spp.	113	126
Odds ratio	95% confidence interval	Significance level P
4.460	0.4910, 40.516	0.1971, not significant

**Table A448:** Odds ratio for the association of *Entamoeba coli* and conidia.

	*Entamoeba coli*	Non-*Entamoeba coli*
Conidia	14	27
Non-conidia	103	100
Odds ratio	95% confidence interval	Significance level P
0.5034	0.2495, 1.016	0.0602, not significant

**Table A449:** Odds ratio for the association of *Entamoeba coli* and pseudoconidia.

	*Entamoeba coli*	Non-*Entamoeba coli*
Pseudoconidia	11	16
Non-pseudoconidia	106	111
Odds ratio	95% confidence interval	Significance level P
0.7199	0.3194, 1.623	0.5409, not significant

**Table A450:** Odds ratio for the association of *Entamoeba dispar* and *Iodamoeba buetschlii*.

	*Entamoeba dispar*	Non-*Entamoeba dispar*
*Iodamoeba buetschlii*	15	40
Non-*Iodamoeba buetschlii*	103	572
Odds ratio	95% confidence interval	Significance level P
2.083	1.110, 3.908	0.0377, significant

**Table A451:** Odds ratio for the association of *Entamoeba dispar* and *Pentatrichomonas hominis*.

	*Entamoeba dispar*	Non-*Entamoeba dispar*
*Pentatrichomonas hominis*	2	1
Non-*Pentatrichomonas hominis*	116	610
Odds ratio	95% confidence interval	Significance level P
10.517	0.9453, 117.01	0.0697, not significant

**Table A452:** Odds ratio for the association of *Entamoeba dispar* and *Encephalocytozoon* spp.

	*Entamoeba dispar*	Non-*Entamoeba dispar*
*Encephalocytozoon* spp.	2	5
Non-*Encephalocytozoon* spp.	87	151
Odds ratio	95% confidence interval	Significance level P
0.6943	0.1318, 3.656	1.0000, not significant

**Table A453:** Odds ratio for the association of *Entamoeba dispar* and *Aeromonas* spp.

	*Entamoeba dispar*	Non-*Entamoeba dispar*
*Aeromonas* spp.	1	4
Non-*Aeromonas* spp.	88	152
Odds ratio	95% confidence interval	Significance level P
0.4318	0.04749, 3.927	0.6558, not significant

**Table A454:** Odds ratio for the association of *Entamoeba dispar* and conidia.

	*Entamoeba dispar*	Non-*Entamoeba dispar*
Conidia	11	36
Non-conidia	78	120
Odds ratio	95% confidence interval	Significance level P
0.4701	0.2258, 0.9785	0.0439, significant

**Table A455:** Odds ratio for the association of *Entamoeba dispar* and pseudoconidia.

	*Entamoeba dispar*	Non-*Entamoeba dispar*
Pseudoconidia	11	16
Non-pseudoconidia	78	140
Odds ratio	95% confidence interval	Significance level P
1.234	0.5455, 2.791	0.6731, not significant

**Table A456:** Odds ratio for the association of *Iodamoeba buetschlii* and *Pentatrichomonas hominis*.

	*Iodamoeba buetschlii*	Non-*Iodamoeba buetschlii*
*Pentatrichomonas hominis*	0	3
Non-*Pentatrichomonas hominis*	55	671
Odds ratio	95% confidence interval	Significance level P
1.728	0.08810, 33.912	1.0000, not significant

**Table A457:** Odds ratio for the association of *Iodamoeba buetschlii* and *Encephalocytozoon* spp.

	*Iodamoeba buetschlii*	Non-*Iodamoeba buetschlii*
*Encephalocytozoon* spp.	0	7
Non-*Encephalocytozoon* spp.	18	220
Odds ratio	95% confidence interval	Significance level P
0.7946	0.04361, 14.477	1.0000, not significant

**Table A458:** Odds ratio for the association of *Iodamoeba buetschlii* and *Aeromonas* spp.

	*Iodamoeba buetschlii*	Non-*Iodamoeba buetschlii*
*Aeromonas* spp.	0	5
Non-*Aeromonas* spp.	18	222
Odds ratio	95% confidence interval	Significance level P
1.093	0.05813, 20.566	1.0000, not significant

**Table A459:** Odds ratio for the association of *Iodamoeba buetschlii* and conidia.

	*Iodamoeba buetschlii*	Non-*Iodamoeba buetschlii*
Conidia	4	37
Non-conidia	14	190
Odds ratio	95% confidence interval	Significance level P
1.467	0.4572, 4.708	0.5140, not significant

**Table A460:** Odds ratio for the association of *Iodamoeba buetschlii* and pseudoconidia.

	*Iodamoeba buetschlii*	Non-*Iodamoeba buetschlii*
Pseudoconidia	3	24
Non-pseudoconidia	15	203
Odds ratio	95% confidence interval	Significance level P
1.692	0.4564, 6.270	0.4295, not significant

**Table A461:** Odds ratio for the association of *Pentatrichomonas hominis* and *Encephalocytozoon* spp.

	*Pentatrichomonas hominis*	Non-*Pentatrichomonas hominis*
*Encephalocytozoon* spp.	0	7
Non-*Encephalocytozoon* spp.	2	235
Odds ratio	95% confidence interval	Significance level P
6.280	0.2765, 142.64	1.0000, not significant

**Table A462:** Odds ratio for the association of *Pentatrichomonas hominis* and *Aeromonas* spp.

	*Pentatrichomonas hominis*	Non-*Pentatrichomonas hominis*
*Aeromonas* spp.	0	5
Non-*Aeromonas* spp.	2	237
Odds ratio	95% confidence interval	Significance level P
8.636	0.3691, 202.07	1.0000, not significant

**Table A463:** Odds ratio for the association of *Pentatrichomonas hominis* and conidia.

	*Pentatrichomonas hominis*	Non-*Pentatrichomonas hominis*
Conidia	1	46
Non-conidia	1	196
Odds ratio	95% confidence interval	Significance level P
4.261	0.2614, 69.443	0.3488, not significant

**Table A464:** Odds ratio for the association of *Pentatrichomonas hominis* and pseudoconidia.

	*Pentatrichomonas hominis*	Non-*Pentatrichomonas hominis*
Pseudoconidia	0	27
Non-pseudoconidia	2	215
Odds ratio	95% confidence interval	Significance level P
1.567	0.07327, 33.525	1.0000, not significant

**Table A465:** Odds ratio for the association of *Encephalocytozoon* spp. and *Aeromonas* spp.

	*Encephalocytozoon* spp.	Non-*Encephalocytozoon* spp.
*Aeromonas* spp.	0	5
Non-*Aeromonas* spp.	7	275
Odds ratio	95% confidence interval	Significance level P
3.339	0.1687, 66.094	1.0000, not significant

**Table A466:** Odds ratio for the association of *Encephalocytozoon* spp. and conidia.

	*Encephalocytozoon* spp.	Non-*Encephalocytozoon* spp.
Conidia	0	41
Non-conidia	7	197
Odds ratio	95% confidence interval	Significance level P
0.3173	0.01776, 5.668	0.6048, not significant

**Table A467:** Odds ratio for the association of *Encephalocytozoon* spp. and pseudoconidia.

	*Encephalocytozoon* spp.	Non-*Encephalocytozoon* spp.
Pseudoconidia	0	27
Non-pseudoconidia	7	211
Odds ratio	95% confidence interval	Significance level P
0.5127	0.02847, 9.234	1.0000, not significant

**Table A468:** Odds ratio for the association of *Aeromonas* spp. and conidia.

	*Aeromonas* spp.	Non-*Aeromonas* spp.
Conidia	2	39
Non-conidia	3	201
Odds ratio	95% confidence interval	Significance level P
3.436	0.5555, 21.253	0.1962, not significant

**Table A469:** Odds ratio for the association of *Aeromonas* spp. and pseudoconidia.

	*Aeromonas* spp.	Non-*Aeromonas* spp.
Pseudoconidia	1	26
Non-pseudoconidia	4	214
Odds ratio	95% confidence interval	Significance level P
2.058	0.2214, 19.124	0.4451, not significant

**Table A470:** Odds ratio for the association of conidia and pseudoconidia.

	Conidia	Non-conidia
Pseudoconidia	13	14
Non-pseudoconidia	28	190
Odds ratio	95% confidence interval	Significance level P
6.301	2.685, 14.787	<0.0001, significant
